# A general synthesis of cyclic bottlebrush polymers with enhanced mechanical properties *via* graft-through ring expansion metathesis polymerization†

**DOI:** 10.1039/d4sc06050d

**Published:** 2024-09-30

**Authors:** Matthew J. Elardo, Adelaide M. Levenson, Ana Paula Kitos Vasconcelos, Meredith N. Pomfret, Matthew R. Golder

**Affiliations:** a Department of Chemistry, Molecular Engineering & Science Institute, University of Washington 36 Bagley Hall Seattle WA 98195 USA goldermr@uw.edu

## Abstract

Bottlebrush polymers represent an important class of macromolecular architectures, with applications ranging from drug delivery to organic electronics. While there is an abundance of literature describing the synthesis, structure, and applications of linear bottlebrush polymers using ring-opening metathesis polymerization (ROMP), there are comparatively less reports on their cyclic counterparts. This lack of research is primarily due to the difficulty in synthesizing cyclic bottlebrush polymers, as extensions of typical routes towards linear bottlebrush polymers (*i.e.*, “grafting-through” polymerizations of macromonomers with ROMP) produce only ultrahigh molar mass cyclic bottlebrush polymers with poor molar mass control. Herein, we report a ring-expansion metathesis polymerization (REMP) approach to cyclic bottlebrush polymers *via* a “grafting-through” approach utilizing the active pyr-CB6 initiator developed in our lab. The resulting polymers, characterized *via* GPC-MALS-IV, are shown to have superior molar mass control across a range of target backbone lengths. The cyclic materials are also found to have superior mechanical properties when compared to their linear counterparts, as assessed by ball-mill grinding and compression testing experiments.

## Abstract 2

Cyclic bottlebrush polymers combine the promise of densely-grafted macromolecular architectures with the mechanical advantages of cyclic polymers for diverse applications across materials science.

## Graphical abstract

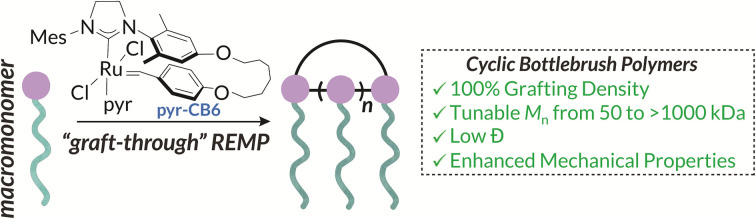

## Introduction

**Fig. 1:**
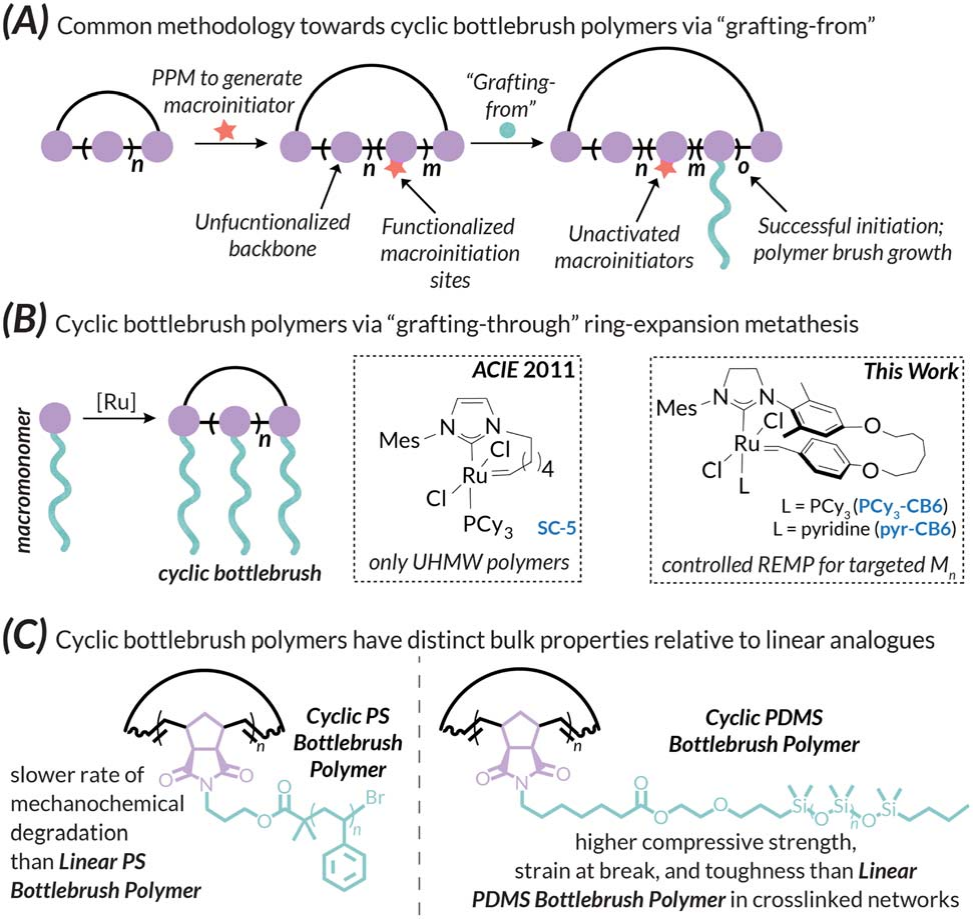
(A) General paradigm for cyclic bottlebrush polymer synthesis *via* “grafting-from”; (B) approaches towards cyclic bottlebrush polymers using Ru-mediated REMP. (C) Summary of bulk property enhancements for cyclic PS-BBP and cyclic PDMS-BBP with respect to comparable linear analogues.

The manipulation of polymer topology is a major area of interest in synthetic macromolecular chemistry due to the impact of architecture on material proprieties. Recent advances in this area have led to the development of a wide variety of intricate synthetic polymer architectures with defined chain ends, such as star polymers,^1^ dendrimers,^2^ and bottlebrush polymers.^3–6^ Of particular interest, however, are variants without chain ends, namely cyclic polymers, as this specific topology has markedly different properties when compared to linear counterparts, such as higher glass transition temperatures, higher decomposition temperatures, and lower intrinsic viscosities.^7–9^ Acyclic bottlebrush polymers^3,6^ are linear polymers with densely grafted macromolecular sidechains; the chemical makeup of the backbone and sidechains intimately control rich solution-state and bulk phenomena including self-assembly^10–12^ and stimuli-responsiveness in thin films.^13^ As such, these polymer bottlebrush architectures have found diverse applications from drug delivery^14–17^ to organic photonics.^18–22^ There has therefore been intense interest in the development of new linear bottlebrush materials using well-established synthetic paradigms. These strategies can be broken into three general categories: “grafting-to”,^23^ “grafting-from”,^24^ and “grafting-through”.^25^ Of these three distinct synthetic classes, the “grafting-through” method is most desirable because of precise control over features such as grafting density (where high densities up to 100% are easily achievable), brush length, and brush dispersity, but is synthetically challenging due to the steric demands of the polymerizable “macromonomer”.^26^ For ROMP-derived materials, this problem has been largely solved by the development of advanced olefin metathesis initiators,^27,28^ which produce bottlebrush polymers with narrow dispersities and good control over molar mass, even when using large and/or branched macromonomers.^12,25,29^ While examples of linear bottlebrush polymers (BBPs) abound in the literature,^6,12–16,18^ instances of their cyclic analogues are comparatively sparse. This difference is due to the various challenges associated with cyclic polymer synthesis. One major synthetic approach towards cyclic BBPs, linear ring closure, involves synthesizing a telechelic linear pre-polymer; functional end groups react with each other to form the desired macrocycle. However, this approach inevitably results in linear contaminants from acyclic couplings, requires dilute reaction conditions, and is not suitable for the generation of high molar mass materials.^30,31^ Nevertheless, this approach allowed access to multiblock cyclic polymers and provided early evidence for unique self-assembly profiles.^31^ The second major synthetic approach is ring expansion polymerization, of which ring expansion metathesis polymerization (REMP, the cyclic analogue to ROMP) is a common variant.^32,33^ Early examples of REMP initiators suffer from poor molar mass control and polydisperse products, especially when performing “graft-through” reactions with large macromonomers.^34^ For instance, Grubbs used this approach to prepare cyclic bottlebrush polymers with macrocyclic alkylidene REMP initiators (*e.g.*, SC-5); however, only ultra-high molar mass materials were reported.^34^ Due to the inherent difficulty in controlled cyclic bottlebrush polymer growth using “grafting-through”, cyclic BBPs are often prepared *via* “grafting-from” (Fig. 1A).^35–40^

Despite their challenging syntheses, cyclic bottlebrush polymers hold promise in enhanced capacity for drug delivery^41,42^ and self-assembly into a variety of nanostructures, (*e.g.*, supramolecular tubes, rods, plates, spheres, and worm architectures^31,43,44^). Hence, there is great potential for the development of functional materials using cyclic bottlebrush polymers made with a “grafting-through” approach (Fig. 1B). In this work, we report a controlled synthetic route toward cyclic bottlebrush polymers utilizing the novel REMP initiator pyr-CB6 developed within our research group (Fig. 1B).^32,45^ We demonstrate excellent control over molar mass with narrow dispersity utilizing macromonomers with disparate thermomechanical properties – “hard” PS-MM (*i.e.*, high *T*_g_, rigid, brittle; PS = polystyrene, Fig. S22–S25†) and “soft” PDMS-MM (*i.e.*, low *T*_g_, flexible, elastomeric; PDMS = polydimethylsiloxane, Fig. S19 and S20†) macromonomers. The resultant polymers are characterized, and their cyclic topologies confirmed, *via* gel permeation chromatography coupled with multi-angle light scattering, differential viscometry, and refractive index detectors (GPC-MALS-IV-RI; see Fig. S26–S33† for representative DRI traces and Fig. S45–S50† for representative Mark–Houwink–Sakurada intrinsic viscosity plots). The thermal, mechanochemical, and mechanical properties of the bulk materials and crosslinked networks thereof were probed *via* a combination of differential scanning calorimetry (DSC), thermogravimetric analysis (TGA), ball-mill grinding mechanochemistry (BMG), and compression testing (Fig. 1C). Overall, the efforts described herein encompass a straightforward approach towards cyclic bottlebrush polymers and currently represent the most well-controlled “grafting-through” methodology to access them to date. In other words, this methodology allows for the preparation of cyclic bottlebrush polymers with the same ease as their acyclic counterparts.

## Synthesis and solution-state analysis

We began our investigations by probing the activity of less active PCy_3_-CB6 for PS-MM and PDMS-MM REMP reactions. Interestingly, even when targeting a relatively short backbone degree of polymerization (backbone DP = 25), PDMS-MM afforded material with much higher-than-expected *M*_*n*_ (by nearly two orders of magnitude) and a broad GPC-RI trace with a sizeable low molar mass shoulder; PS-MM afforded material that was too viscous for subsequent analysis (Table 1 and Fig. S40†). Despite PCy_3_-CB6 providing superior molar mass control relative to those of cyclic alkylidene initiators (*e.g.*, UC-5, UC-6) in our prior REMP studies with norbornene monomers,^32^ the steric hindrance of macromonomers provide additional kinetic challenges. Fortunately, initial REMP experiments employing the more active pyr-CB6 initiator indicated rapid and complete macromonomer consumption (<1 h) in DCE at 55 °C (Fig. S36 and S37†). Notably, we do not observe any molar mass evolution at extended reaction times following macromonomer conversion (Fig. S36 and S37†), a feature we reported previously which distinguishes pyr-CB6 mechanistically from PCy_3_-CB6.^45^ Furthermore, we observe a significant improvement in molar mass control, dispersity, and peak structure when utilizing pyr-CB6 in place of PCy_3_-CB6 for “grafting-through” REMP towards well-defined cyclic bottlebrush macromolecules. We believe that an increased initiator efficiency combined with relatively slow secondary metathesis (*i.e.*, intermolecular chain transfer) leads to the large disparity in initiator-dependent experimental *M*_*n*_ (Table 1).

**Table 1:** GPC characterization of PDMS-MM^a^ REMP initiated by CB6 (with and without pyridine)

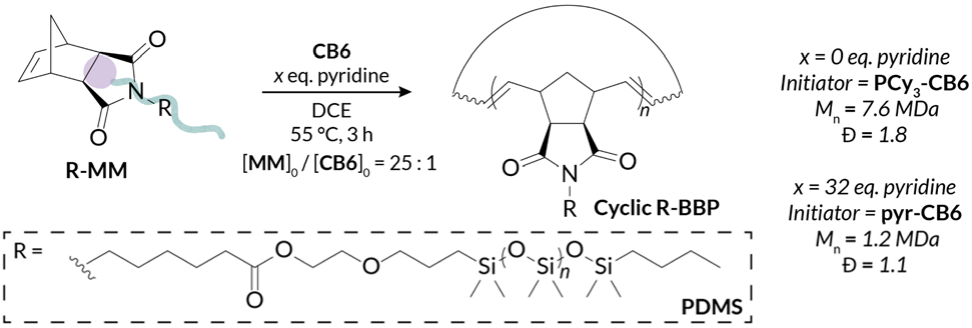					
Pyridine eq.	Theoretical DP^b^	Experimental DP^c^	Theoretical *M*_*n*_^b^ (kDa)	Experimental *M*_*n*_^c^ (kDa)	*Đ* c
0	25	1090	180	7600	1.8
32	25	171	180	1200	1.1

a PDMS-MM*M*_*n*_ = 7.0 kDa.

b Calculated by [MM]_0_/[*I*]_0_, where [MM] is the molar amount of PDMS-MM and [*I*] is the molar amount of CB6.

c Determined by GPC-MALS.

**Fig. 2:**
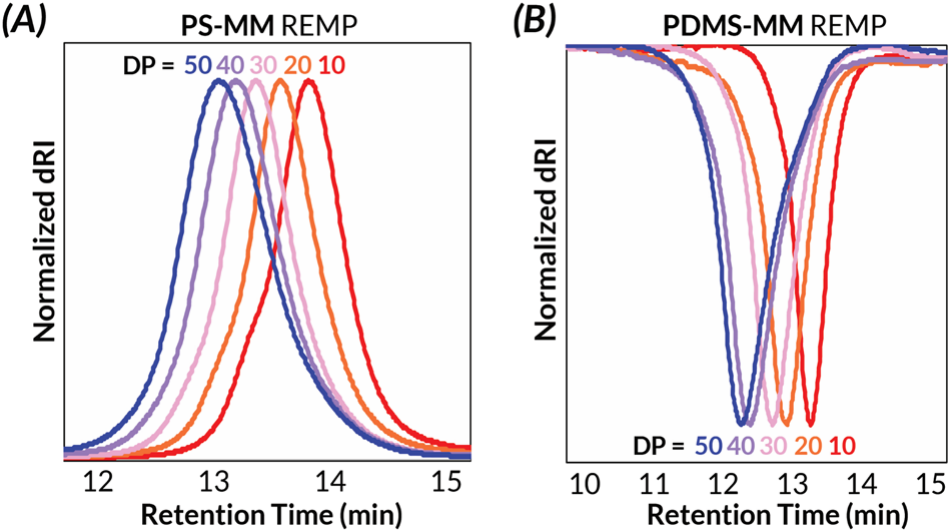
Solution-state characterization of (A) PS-MM and (B) PDMS-MM REMP by GPC-MALS-RI for target DP = 10–50.

We next investigated the ability of our pyr-CB6 initiator to prepare cyclic bottlebrush polymers at a variety of target molar masses (*i.e.*, a range of target backbone DP). A major challenge encountered with cyclic Ru alkylidene initiators utilized by Grubbs is their inability to prepare low DP polymers due to poor initiation efficiency; this shortcoming is especially true when targeting bottlebrush polymers *via* “grafting-through” of macromonomers.^34^ We observe good molar mass control over a wide range of target DPs (DP = 10–50) (Table 2) while maintaining low dispersities for both PS-MM (Fig. 2A) and PDMS-MM (Fig. 2B). The result of the improved initiation efficiency is cyclic bottlebrush polymers with *M*_*n*_ < 50 kDa, a significant improvement from previous systems which were limited to ultra-high molar mass polymers in the MDa regime. Hence, our methodology showcases the most powerful examples to date of controlling cyclic bottlebrush backbone DP with “grafting-through” technology.

**Table 2:** GPC characterization of cyclic REMP BBPs at varying backbone target degrees of polymerization (DP)

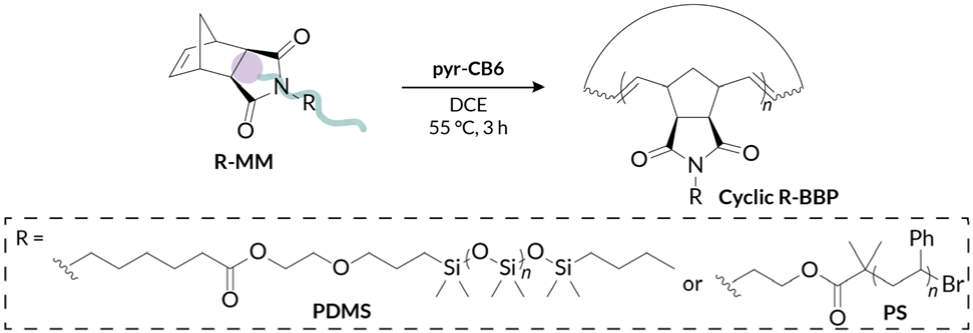					
Monomer^a^	Theoretical DP^b^	Measured DP^c^	Theoretical *M*_*n*_^b^ (kDa)	Measured *M*_*n*_^c^ (kDa)	*Đ* c
PS-MM	10	18	46.0	83.0	1.1
PS-MM	20	28	90.0	130	1.1
PS-MM	30	37	140	170	1.2
PS-MM	40	43	180	200	1.2
PS-MM	50	52	230	240	1.3
PDMS-MM	10	41	63.0	260	1.1
PDMS-MM	20	81	130	510	1.1
PDMS-MM	30	114	190	720	1.1
PDMS-MM	40	151	250	950	1.2
PDMS-MM	50	175	320	1100	1.2

a PS-MM*M*_*n*_ = 4.6 kDa; PDMS-MM*M*_*n*_ = 6.3 kDa.

b Calculated by [MM]_0_/[*I*]_0_, where [MM] is the molar amount of macromonomer and [*I*] is the molar amount of CB6 (plus 32 eq. pyridine).

c Determined by GPC-MALS.

**Fig. 3:**
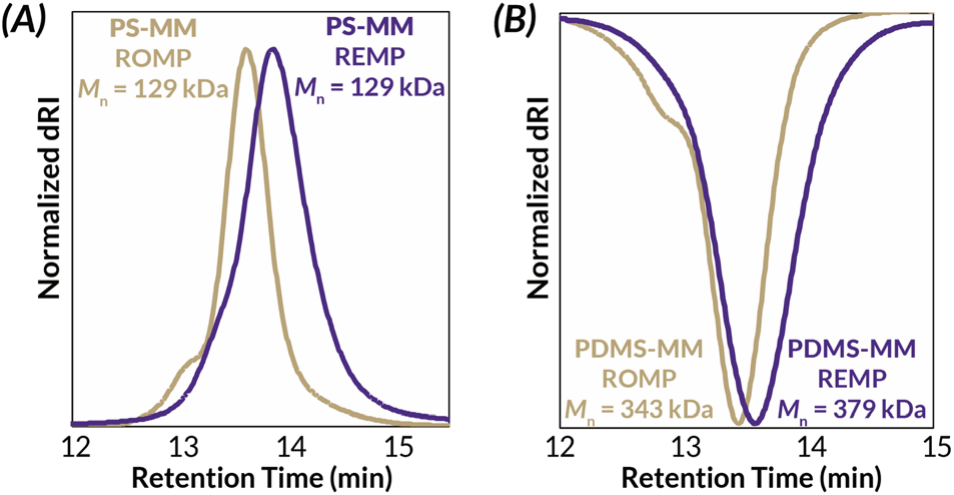
Solution-state characterization of cyclic and linear (A) PS-BBP and (B) PDMS-BBP by GPC-MALS-RI.

Another challenge with preparing cyclic bottlebrush polymers *via* “grafting-through” REMP is that the polymerizations do not work well even with modest sized macromonomers. For instance, in the seminal report of this methodology from Grubbs using SC-5 and SC-6, they observe only 65% conversion of PS-MM (*M*_*n*_ = 6.6 kDa).^34^ Here, we demonstrate that analogous REMP utilizing pyr-CB6 works well for even larger macromonomers; increasing the brush length (PS-MM, *M*_*n*_ = 8.3 kDa) does not have a detrimental impact on molar mass control and only modestly increases the dispersity of the resulting cyclic bottlebrush polymer (Fig. S38 and Table S3†). Although more dilute conditions are required to maintain molar mass control (*ca.* 40 mg mL^−1^, *versus ca.* 90 mg mL^−1^ for the lower molar mass PS-MM), the reaction is nearly quantitative with >90% monomer conversion after 3.5 hours as assessed by GPC-RI (Fig. S39†). We next probed the topology of the putative cyclic bottlebrush polymers in dilute solution *via* GPC with in-line multi-angle light scattering, differential viscometry, and refractive index detectors (GPC-MALS-IV-RI). To determine absolute molar masses, we directly measured the specific refractive index increment (*i.e.*, dn/dc) for representative ROMP and REMP bottlebrush polymers (Fig. S51–S58 and Table S4†). Interestingly, cyclic bottlebrush polymers derived from both PS-MM and PDMS-MM had lower magnitude dn/dc values relative to linear analogs in CHCl_3_; topology dependent refractive indices have been observed in other organic polymer scaffolds as well.^46–48^ In general, we observe longer retention times in the GPC analysis of REMP bottlebrush polymers when compared to ROMP bottlebrush polymers of similar molar masses prepared using Grubbs 3 initiator (see ESI† for Experimental details) for both PS-MM (Fig. 3A) and PDMS-MM (Fig. 3B) REMP reactions. Likewise, plots of molar mass *vs.* GPC retention time indicate higher molar masses for the REMP polymers across all elution volumes (Fig. S43 and S44†). These results collectively indicate that the REMP polymers are more compact in solution than their ROMP counterparts of similar molar mass, a feature that is characteristic of cyclic polymers due to their lack of chain ends.^7–9^

**Fig. 4:**
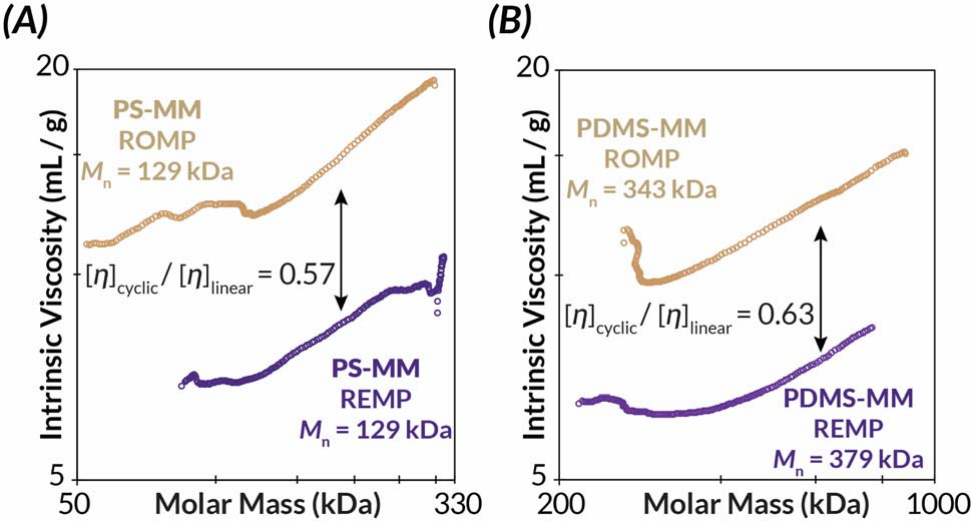
Mark–Houwink–Sakurada plots of cyclic and linear (A) PS-BPP and (B) PDMS-BBP as measured by GPC-MALS-IV-RI.

Similarly, Mark–Houwink–Sakurada (MHS) analyses of the REMP BBPs reveal significantly lower intrinsic viscosities than for ROMP BBPs of similar molar mass ([*η*]_cyclic_/[*η*]_linear_ = *ca.* 0.57–0.63) for both PS (Fig. 4A) and PDMS (Fig. 4B).^7–9^ As the bottlebrush polymers presented in this study have comparatively short backbone DPs relative to prior “grafting-through” REMP studies,^34^ dilute solution-state behavior as assessed by intrinsic viscosity measurements suggests deviation from Flory–Fox behavior (*i.e.*, [*η*] ∼ *M*^0.7^).^49^ Specifically, only a small increase in intrinsic viscosity with increasing molar mass is observed with a Mark–Houwink parameter, *α*, between *ca.* 0.3–0.4 (Fig. S45–S50†). These data suggest that the bottlebrush polymers in this study, all with relatively short backbone DPs, behave more like star-polymers in solution.^49^ Nonetheless, importantly both ROMP and REMP polymers have similar Mark–Houwink parameters and therefore differences in the observed intrinsic viscosities are due to molecular architecture (*i.e.*, linear *versus* cyclic) rather than differences in backbone structure and/or conformation.^33^

We also used the collective analytical approaches described above to qualitatively probe the efficiency of polymer backbiting by comparing the GPC-RI traces and MHS plots of quenched (*i.e.*, ethyl vinyl ether, EVE, was added at the end of the polymerization reaction) and unquenched aliquots from the same samples (Fig. S34, S35, S47, S50 and Tables S1, S2†). If backbiting is incomplete and Ru remains in the cyclic polymer backbone, quenching with EVE produces linear polymers following macrocycle opening *via* cross-metathesis. This process would result in a shift to shorter retention times and higher intrinsic viscosities in the GPC-RI traces and MHS plots, respectively. We see no significant difference in either of these parameters between quenched and unquenched aliquots from our reaction mixtures, indicating that backbiting is operative insofar as we can measure.

## Thermal and mechanical properties

**Fig. 5:**
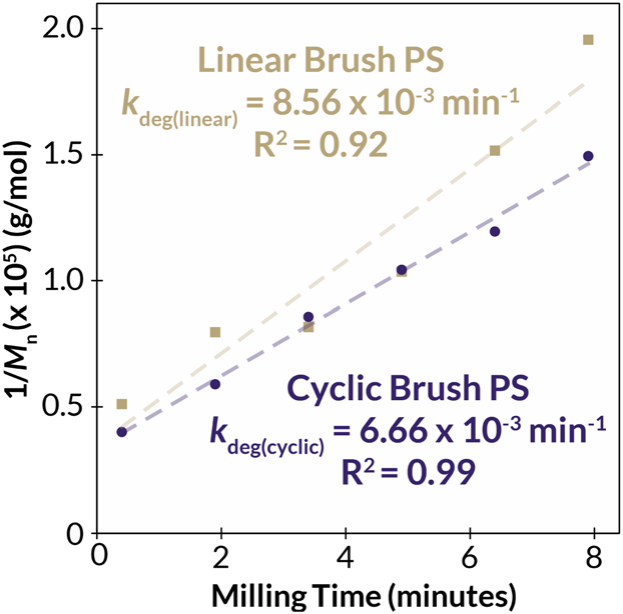
Degradation kinetics for cyclic (purple) and linear (gold) PS-BBPs under ball-mill grinding conditions. The inverse of the number-average molar mass was determined for each polymer at each timepoint, and linear regressions were performed to determine the line of best fit (dashed lines). Rate constants were determined by multiplying the slope of each trendline by the number average molar mass of PS-MM. See ESI Section 8† for details.

With two classes of cyclic bottlebrush polymers in hand, we next turned our attention to evaluating the impact of topology on bulk properties of our bottlebrush polymers. We began by probing the thermal properties of linear and cyclic PS bottlebrush polymers (*M*_*n*_ = 129 kDa), by thermal gravimetric analysis (TGA) and differential scanning calorimetry. Interestingly, we found no significant differences in the thermal stability *via* TGA (*T*_d_ = 374 °C and 368 °C for linear and cyclic PS-BBPs, respectively) or thermal transition temperature *via* DSC (*T*_g_ = 100 °C and 95 °C for linear and cyclic PS-BBPs, respectively) between the linear and cyclic samples (Fig. S59–S62†). While cyclic polymers are known to exhibit higher decomposition temperatures (*T*_d_) and glass transition (*T*_g_) temperatures, we surmise that the high mass percentage of polymeric brush in each sample obfuscates subtle topology-dependent thermal differences. Because of the similar thermal properties of our PS bottlebrush polymers, we next decided to probe the mechanical stability of the bulk PS brushes by subjecting them to ball-mill grinding (BMG) conditions. While separate studies have been conducted by Peterson, Kim, Hwang, and Choi probing the relative independent stability of cyclic^50^ and bottlebrush^51^ polymers to linear analogues under ball-milling conditions, no work has been done exploring the relative stability of cyclic bottlebrush polymers under these conditions. Since it has been previously demonstrated that the degradation rates of linear polymers under BMG conditions scale linearly with increasing *T*_g_,^52^ a feature not observed in either cyclic^50^ or bottlebrush systems,^51^ and that cyclic polymers tend to degrade slower than linear polymers with of comparable *M*_*n*_ under these conditions,^50^ we hypothesized that the cyclic brush polymers may have unique stability to mechanochemical degradation in the solid state. Indeed, we observed that a linear PS-BBP (*M*_*n*_ = 200 kDa) degraded approximately 30% faster than a cyclic PS-BBP (*M*_*n*_ = 250 kDa) (Fig. 5, S41 and S42; see ESI Section 8† for Experimental details and Tables S5 and S6† for raw data). Thus, we have demonstrated the ability to create materials with enhanced mechanical stability without perturbing thermal properties. This finding, in conjunction with previous findings demonstrating the improved wear resistance and shear stability of cyclic polymer brushes,^53^ may be desirable for applications in polymer coatings such as antifouling materials, where abrasion and impact damage shorten the material's lifespan.^54^

**Fig. 6:**
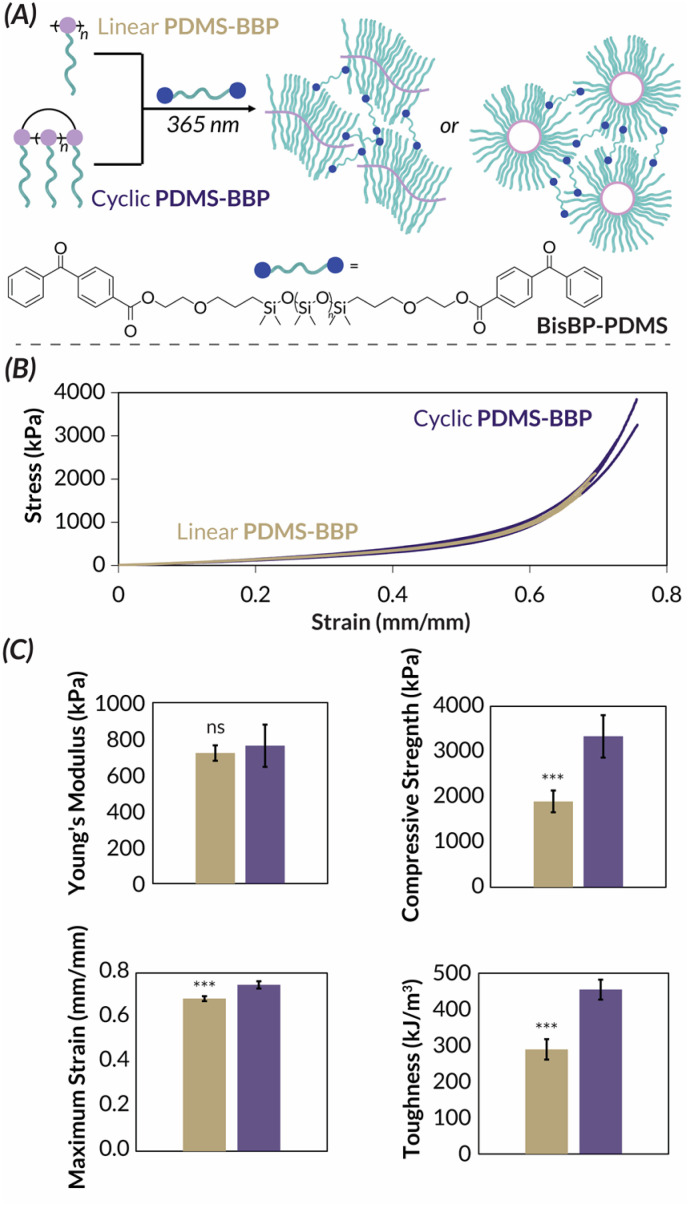
(A) Cartoon demonstrating cross-linking chemistry. (B) Stress–strain curve for linear (gold) and cyclic (purple) PDSM-BBP networks in compression tests. (C) Selected mechanical properties of linear (gold) and cyclic (purple) PDMS-BBP networks. *** *p* < 0.01 for *n* = 3. ns: no significance (*p* > 0.05) for *n* = 3.

We next turned our attention to the PDMS-BBP samples for bulk characterization. These studies were inspired by the universal bottlebrush polymer crosslinking methodology developed by Bates^55^ previously used to prepare supersoft elastomers from PDMS bottlebrush polymers.^56^ In this work, bifunctional benzophenone BisBP-PDMS initiator facilitated indiscriminate brush C–H abstraction and subsequent curing under photochemical irradiation (Fig. 6A). We reasoned that these mild crosslinking conditions would allow us to explore the impact of bottlebrush polymer topology on the resulting materials' mechanical properties. While we are the first to explore the mechanical properties of crosslinked cyclic bottlebrush networks, previous independent work from Tew, Sun, and Veige and Sumerlin have found that cyclic polymer networks have greater compressibility^57^ and stretchability,^58^ greater swelling ratios in organic solvent,^57,59^ and increased toughness^58^ when compared to networks prepared from linear analogues. Therefore, we reasoned that such phenomena would extend to our cyclic PDMS-BBP networks and hypothesized that they should have superior mechanical properties compared to networks made from linear PDMS-BBP at the same crosslinking density. We accordingly synthesized crosslinked networks from high molar mass linear (ROMP, *M*_*n*_ = 1.73 MDa) and cyclic (REMP, *M*_*n*_ = 3.49 MDa) PDMS-BBP samples with a crosslinking density (*χ*) of 1 mol BisBP-PDMS per individual brush in the bottlebrush polymer (*i.e.*, for *χ* = 1.00, *n*_crosslinker_ = *n*_bottlebrush_ × DP_bottlebrush_). It should be noted that while there is a slight mismatch between bottlebrush polymer absolute molar masses, because cyclic polymers are more compact than their linear counterparts, the PDMS-BBP precursors to these network samples are actually quite comparable due to similar radii of gyration (*R*_g_ = *ca.* 20–25 nm). Furthermore, in their work with cyclic polymer gels, Tew found no change in mechanical properties upon doubling the molar mass of the linear polymer precursor,^57^ and Sumerlin and Veige reported mechanical data for cyclic polymers *ca.* 1.5 times larger than their linear analogues.^59^ We thus reasoned that the slightly higher molar mass of our cyclic BBPs was unlikely to obfuscate any difference in mechanical properties between the samples. Consistent with reports on the aforementioned cyclic polymer networks (*vide supra*), our cyclic PDMS-BBP demonstrated significantly (*p* < 0.001) higher swelling ratios (191%) in ethyl acetate than their linear analogues (148%) (Table S7†). Upon compression testing of freshly cured cylindrical specimens (Fig. 6B), indeed while both networks had similar Young's moduli (762 and 721 kPa, respectively), networks prepared from cyclic PDMS-BBP demonstrated statistically significant increases in compressive strength, strain at break, and toughness (*p* < 0.01 for all parameters, *n* = 3) compared to those prepared from linear PDMS-BBP at *χ* = 1.00 (Fig. 6C; see Fig. S63–S67† for statistical analysis results and Table S7† for numerical values).

With such broad utility for PDMS-based bottlebrush networks across applications in soft robotics,^60^ sensors,^56,61^ and electronics,^62^ it is significant that polymer topology alone can enhance mechanical properties. More generally, we anticipate these collective findings to advance the molecular engineering of mechanically robust bottlebrush polymers networks^63^ where backbone topology, rather than chemical composition, controls bulk behavior.

## Conclusions

In conclusion, we demonstrate in this report an efficient and general methodology for the synthesis of macrocyclic bottlebrush polymers *via* “grafting-through”. Our approach, which takes advantage of the superior initiation efficiency of our cyclic benzylidene REMP initiator pyr-CB6, produces densely grafted macrocyclic bottlebrush polymers with good control over molar mass and dispersity. We report a significant improvement over previous cyclic Ru alkylidene initiators to this end, especially with respect to preparing lower molar mass (<100 kDa) backbones and polymers with long brush lengths (>5 kDa). We demonstrate that the methodology is general, with the ability to tune the parent macromonomer composition and resultant backbone length easily and independently. The cyclic topology of these bottlebrush polymers was interrogated using solution-state analyses (GPC-MALS-IV-RI). Moreover, the bulk thermal and mechanical properties of the BBPs were probed. It was found that despite similar thermal properties, the cyclic PS-BBP materials are more stable to mechanochemical degradation than their linear counterparts under ball-mill grinding conditions. Furthermore, we found significant enhancements to the mechanical properties of crosslinked PDMS-BBP elastomers with cyclic topologies. Compared to networks prepared from linear PDMS-BBP, our elastomers prepared from cyclic PDMS-BBP had statistically significant higher compressive strength, strain at break, toughness, and swelling ratio. We envision this methodology finding broad appeal in the development of well-defined macrocyclic bottlebrush polymers for applications spanning porous materials, mechanically resilient coatings/lubricants, tougher soft robotics, and self-assembled nanoarchitectures.

## Author contributions

M. J. E., A. M. L., and M. R. G. conceived of the idea. M. J. E., A. M. L., and M. N. P. conducted synthetic experiments and analyzed physical properties. M. J. E. conducted ball-milling experiments. A. P. K. V. performed mechanical testing experiments. M. J. E. and M. R. G. wrote the manuscript; all authors discussed and edited the manuscript.

## Conflicts of interest

There are no conflicts to declare.

## Supplementary Material

SC-015-D4SC06050D-s001

## Data Availability

The data supporting this article have been included as part of the ESI.†
