# Supplementary material for: A general synthesis of cyclic bottlebrush polymers with enhanced mechanical properties via graft-through ring expansion metathesis polymerization
Source: Chem Sci. 2024 Sep 30;15(41):17193–9. doi: 10.1039/d4sc06050d (PMC11440813; doi:10.1039/d4sc06050d)
Supplement: SC-015-D4SC06050D-s001 [file SC-015-D4SC06050D-s001.pdf]

Supplementary Information (SI) for Chemical Science.
This journal is © The Royal Society of Chemistry 2024

Supporting Information For:

A General Synthesis of Cyclic Bottlebrush Polymers with Enhanced Mechanical Properties via Graft-Through Ring Expansion Metathesis Polymerization

Matthew J. Elardo, Adelaide M. Levenson, Ana Paula Kitos Vasconcelos, Meredith N. Pomfret, and Matthew R. Golder*

Department of Chemistry and Molecular Engineering and Science Institute
University of Washington, Seattle, WA 98195, United States

Corresponding author: goldermr@uw.edu

Table of Contents:

1. General Considerations....2
2. Synthetic and Experimental Procedures....3
    a. Synthesis of PDMS Macromonomer & Crosslinker....3
    b. Synthesis of PS Macromonomer....5
    c. Synthesis of Metathesis Catalysts....7
    d. Synthesis of Bottlebrush Polymers....13
3. NMR Spectroscopy Data....17
4. GPC-MALS-RI Data....34
5. GPC-MALS-IV Data....51
6. Batch Injection Procedures and Data....55
7. Thermal Characterization....61
8. Ball-Mill Grinding Kinetics Experimental Procedures and Data....64
9. Preparation of PDMS Networks and Compression Testing. 66
10. References....69

1: GENERAL CONSIDERATIONS:

Materials

All reagents were purchased from commercial suppliers and used as received unless otherwise noted. Glassware was flame dried or dried in an oven overnight at 120 °C before use. Degassed and anhydrous tetrahydrofuran (THF) was obtained from a JC Meyer solvent purification system. Dimethylformamide (DMF) was dried over 3Å molecular sieves for at least 3 days before use. 1,2-dichloroethane (DCE) was fractionally distilled and stored over 3Å molecular sieves before use. Pyridine (anhydrous) was sparged for 1 h with nitrogen and stored over 3Å molecular sieves in the glovebox before use. Triethyl orthoformate was dried over sodium sulfate and distilled from potassium hydroxide, degassed, and stored over 3Å molecular sieves before use. All moisture and air-sensitive reactions were performed under inert atmosphere (nitrogen) using standard Schlenk technique or, when noted, in a Vacuum Atmosphere OMNI glovebox or Innovative Technologies glovebox. SiliaFlash F60 (40-63 μm, 230-400 mesh) silica gel was used for column chromatography. Automated flash chromatography was performed using a Yamazen Smart Flash AKROS system. Preparative-scale gel permeation chromatography (prep-GPC) was performed using a Japan Analytical Industries LaboACE recycling preparative HPLC system equipped with JAIGEL-2.5HR and JAIGEL-3HR columns in series using chloroform (stabilized with 0.5% - 1.0% ethanol) as the mobile phase. Ball-milling experiments were performed using a Retsch Mixer Mill (MM 400) instrument in 5 mL stainless steel screw-top milling jars with two 8 mm stainless steel grinding balls (MSE Supplies LLC).

Characterization

^{1}H nuclear magnetic resonance (^{1}H NMR) spectra were taken on a Bruker AVANCE-NEO at 500 MHz. ^{13}C 1D nuclear magnetic resonance (^{13}C NMR) spectra were recorded on a Bruker AVANCE-NEO at 125 MHz. ^{1}H NMR spectra were taken in chloroform-*d* with TMS ($CDCl_3$, referenced to residual protio-solvent, δ 7.16 ppm), benzene-d_6 (C_6D_6, referenced to residual protio-solvent, δ 7.16 ppm), methanol-d_4 (CD_3OD, referenced to residual protio-solvent, δ 3.31 ppm), or acetone-d_6 ($CO(CD_3)_2$), referenced to residual protio-solvent, δ 2.05 ppm). ^{13}C NMR spectra were taken in chloroform-*d* ($CDCl_3$, referenced to solvent, δ 77.16 ppm), benzene-d_6 (C_6D_6, referenced to solvent, δ 128.06 ppm), or methanol-d_4 (CD_3OD, referenced to solvent, δ 49.00 ppm). Spectra were analyzed on MestreNova software. Chemical shifts are represented in parts per million (ppm); splitting patterns are assigned as s (singlet), d (doublet), t (triplet), q (quartet), m (multiplet), and br (broad); coupling constants, *J*, are reported in hertz (Hz).

Analytical gel permeation chromatography (GPC) data were collected on Agilent 1260 HPLC equipped with a Wyatt 8-angle DAWN NEON light-scattering detector, ViscoStar NEON viscometer, and Optilab NEON refractive index detector. GPC samples were analyzed at a flow rate of 1 mL/min in chloroform (stabilized with 0.5 – 1.0% ethanol) through two Agilent PLgel MIXED-C columns at 35 °C. *dn/dc* values were estimated by the 100% mass recovery method or measured directly by batch injection, as specified, using Wyatt ASTRA 8.2.2 software.

Thermogravimetric analysis (TGA) data were collected using a TA Discovery Q5000 thermogravimetric analyzer. Samples were heated in aluminum Tzero pans at a rate of 10 °C per minute from 23 °C to 500 °C under an N_2 atmosphere. The decomposition temperature was defined as the temperature required to reach 10% mass loss ($T_{d(10\%)}$). Differential scanning calorimetry (DSC) data were collected using a TA Discovery DSC 2500. Samples were placed in hermetically sealed aluminum Tzero pans and heated from 23 °C to 150 °C, cooled to –90 °C, and heated again to 150 °C. During the heating cycles, the temperature was increased at a ramp rate of 5 °C/minute. During the cooling cycles, the temperature was decreased at a ramp rate of –15 °C per minute. Thermal features were analyzed during the second heating cycle using TA Instruments Trios Software v5.0.0.44616.

UV curing was performed with a 365 nm UV light (Mightex, WheeLED, 200 mW/cm^2). Cylinders were prepared with a 3 mm biopsy punch (Royaltek). Compression tests were performed using a Universal Test Machine (Test Resources, 100-25-12) with a 43N load cell. SPSS software version 29.0.1.0 (171) was used to conduct statistical analyses and to calculate all *p*-values disclosed within. Means were compared via 2-tailed Independent Samples *t*-tests.

2: SYNTHETIC AND EXPERIMENTAL PROCEDURES:

Synthesis of PDMS Macromonomer & Crosslinker

Synthesis of *N*-(hexanoic acid)-cis-5-norbornene-exo-dicarboximide (Nb-COOH)

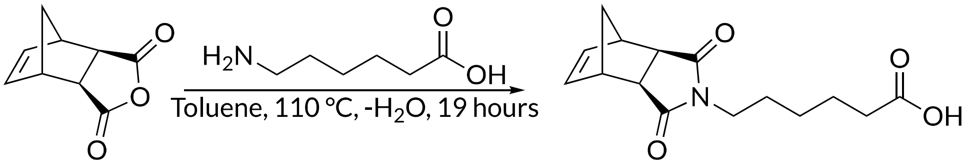

A round bottomed flask was charged with *cis*-norbornene-exo-2,3-dicarboxylic anhydride (1.00 g, 6.09 mmol, 1.00 eq.), 6-aminohexanoic acid (0.800 g, 6.09 mmol, 1.00 eq.), triethylamine (0.084 mL, 0.609 mmol, 0.100 eq.), and toluene (6.37 mL; 0.956 M in norbornene). The apparatus was fitted with a stirring bar, Dean Stark trap, and reflux condenser, and the solution was stirred at 110 °C for 19 hours. Upon completion of the reaction, the flask was cooled to room temperature and the solution was concentrated under vacuum. The crude material was dissolved in dichloromethane (10 mL) and washed with water (3 x 20 mL) and brine (3 x 20 ml). The organic layer was dried with sodium sulfate, filtered, and concentrated under vacuum to afford the product **Nb-COOH** as a colorless solid (1.59 g, 94%). NMR data is in agreement with reported literature values[1]. ^{1}H NMR (500 MHz, $CDCl_3$) δ 6.28 (t, *J* = 1.7 Hz, 2H), 3.47 (t, *J* = 7.6 Hz, 2H), 3.27 (t, *J* = 1.6 Hz, 2H), 2.35 (t, *J* = 7.3 Hz, 2H), 1.71 – 1.18 (m, 10H).

Synthesis of PDMS Macromonomer (PDMS-MM)

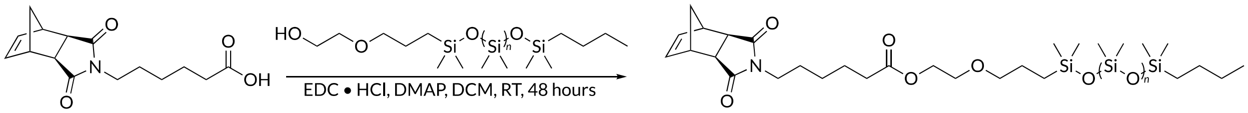

A 50 mL round-bottomed flask was charged with **Nb-COOG** (400 mg, 1.44 mmol, 1.00 eq.), EDC•HCl (387 mg, 2.02 mmol, 1.40 eq) and DMAP (35.2 mg, 0.288 mmol, 0.200 eq). A stirring bar was added to the mixture, the solids were dissolved in DCM (13 mL; 33.3 mL/g norbornene), and the solution was stirred for five minutes at room temperature. Subsequently, alcohol terminated PDMS with M_n = 5.0 kDa (6.06 g, 1.21 mmol, 0.840 eq.) was added and the reaction was stirred at room temperature for 48 hours. The solution was washed with 1M aqueous HCl (3 x 25 mL), water (3 x 25 mL) and brine (3 x 25 mL). The organic layer was dried with sodium sulfate, filtered, and concentrated under vacuum. The product was filtered through a plug of silica in DCM to give the product **PDMS Macromonomer** as a viscous, colorless oil (3.44 g, 54%). The dried material was redissolved in chloroform and filtered through a 0.2 μm syringe filter for GPC-MALS analysis: M_n = 7.0 kDa, $Đ$ = 1.05 *dn/dc* (estimated via 100% mass recovery) = –0.0232. NMR data is in agreement with the literature values[2]. ^{1}H NMR (500 MHz, $CDCl_3$) δ 6.28 (t, *J* = 1.9 Hz, 2H), 4.21 (t, *J* = 5.0 Hz, 2H), 3.44 (dt, *J* = 17.4, 7.3 Hz, 4H), 3.27 (t, *J* = 1.7 Hz, 2H), 2.67 (d, *J* = 1.4 Hz, 2H), 2.33 (t, *J* = 7.5 Hz, 2H), 1.69 – 1.55 (m, 6H), 1.53 (s, 10H), 1.37 – 1.19 (m, 8H), 0.88 (t, *J* = 6.9 Hz, 3H), 0.57 – 0.48 (m, 4H), 0.07 (s, 386H).

Synthesis of PDMS Crosslinker (BisBP-PDMS)

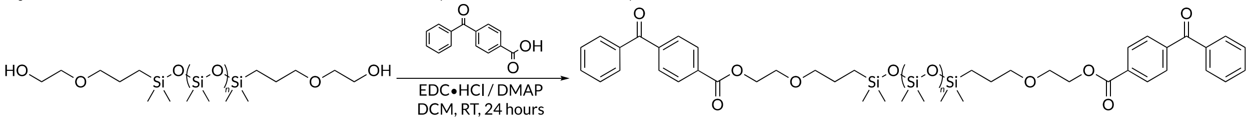

A flask was charged with telechelic hydroxyl terminated PDMS (Gelest DMS-C21; 10.3 g, 2.46 mmol ($M_{n(NMR)}$ = 4.2 kDa), 1.00 eq), 4-benzoylbenzoic acid (4-BBP; 1.40 g, 6.19 mmol, 2.52 eq.), 1-Ethyl-3-(3-dimethylaminopropyl)carbodiimide hydrochloride salt (EDC•HCl; 1.6 g, 8.25 mmol, 3.36 eq.), and 4-(Dimethylamino)pyridine (DMAP; 126 mg, 1.03 mmol, 0.420 eq.). The materials were dissolved in 41 mL DCM (0.2 M in EDC•HCl), and the opaque solution was stirred at RT for 24 hours. The crude solution was washed subsequently with 10% HCl (7 x 50 mL), saturated aqueous sodium bicarbonate (2 x 100 mL), and deionized water (1 x 100 mL). The combined aqueous phases were back extracted with DCM (1 x 75 mL), and the combined organic phase was washed with brine (1 x 150 mL) and dried over sodium sulfate. The suspension was filtered and the filtrate was reduced *in vacuo* to deliver a crude oil, which was passed over a plug of activated basic alumina. The plug was rinsed with 50 mL DCM and the solution was concentrated to deliver the product **BisBP-PDMS** as a colorless oil (6.0 g, 53%). ^{1}H NMR (500 MHz, Acetone) δ 8.19 (d, *J* = 6.5 Hz, 4H), 7.85 (dd, *J* = 17.2, 8.2 Hz, 8H), 7.70 (t, *J* = 7.4 Hz, 2H), 7.58 (t, *J* = 7.7 Hz, 4H), 4.50 (t, *J* = 4.8 Hz, 4H), 3.80 (t, *J* = 5.1 Hz, 4H), 3.51 (t, *J* = 6.7 Hz, 4H), 1.70 – 1.59 (m, 4H), 0.66 – 0.57 (m, 4H), 0.13 (s, 427H). $M_{n(NMR)}$ = 5.8 kDa. See **SI Figure 21** for GPC characterization. ^{1}H NMR taken in $CDCl_3$ also agrees with values reported in the literature.[5]

Synthesis of PS Macromonomer:

Synthesis of *N*-(ethanol)-cis-5-norbornene-exo-dicarboximide (Nb-OH)

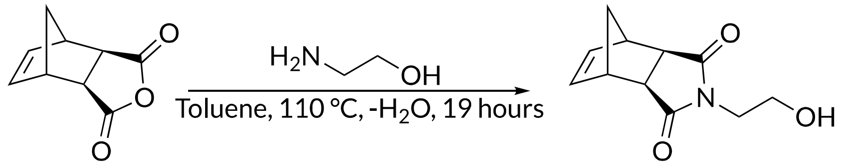

A 500 mL round bottomed flask was charged with *cis*-norbornene-exo-2,3-dicarboxylic anhydride (10.0 g, 60.9 mmol 1.00 eq.), and the apparatus was fitted with a stirring bar, Dean Stark trap, and reflux condenser. The solid was suspended in toluene (150 mL; 0.406 M in norbornene), and ethanolamine (3.87 mL, 3.91 g, 64.0 mmol, 1.05 eq.) was added. The suspension was heated to 110 °C, at which point it became a colorless solution. The solution was refluxed for 19 hours, and the toluene was removed under reduced pressure. The reside was redissolved in boiling ethyl acetate (250 mL), which was subsequently washed with 1 M aqueous HCl (2 x 250 mL) and brine (1 x 150 mL). The organic phase was dried over sodium sulfate, filtered, and concentrated under reduced pressure to afford the product **Nb-OH** as a colorless powder (6.10 g, 48%). NMR data is in agreement with the values reported in the literature[3]. ^{1}H NMR (500 MHz, $CDCl_3$) δ 6.29 (t, *J* = 1.7 Hz, 2H), 3.78 (q, *J* = 4.9 Hz, 2H), 3.72 – 3.68 (m, 2H), 3.28 (t, *J* = 1.6 Hz, 2H), 2.72 (d, *J* = 1.2 Hz, 2H), 1.52 (dt, *J* = 9.9, 1.4 Hz, 1H), 1.35 (dt, *J* = 10.0, 1.5 Hz, 1H).

Synthesis of Norbornene-anchored ATRP initiator (Nb-ATRP)

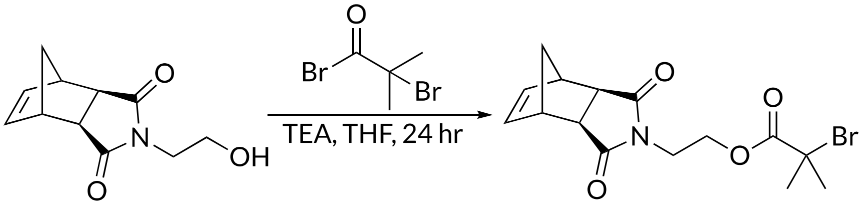

A 250 mL Schlenk flask was charged with a stirring bar and flame dried under vacuum. The flask backfilled with nitrogen, and *N*-(ethanol)-*cis*-5-norbornene-exo-dicarboximide (6.10 g, 29.4 mmol 1.00 eq.) was added to the flask under a constant stream of nitrogen. The flask was evacuated and backfilled again with nitrogen, and the solid was dissolved in anhydrous THF (183 mL; 0.161 M in norbornene). Triethylamine (6.15 mL, 4.47 g, 44.2 mmol, 1.50 eq.) was added and the solution cooled to 0 °C, at which point isobutryl bromide (4.73 mL, 8.80 g, 38.3 mmol, 1.30 eq.) was added dropwise. As the isobutryl bromide was added, a colorless precipitate formed. After the addition was completed, the suspension was stirred in the ice bath and allowed to slowly warm to room temperature overnight. After 24 hours, the suspension was filtered over Celite, and the residue rinsed with DCM (1 x 25 mL). The filtrate was washed with saturated sodium bicarbonate (1 x 150 mL), dried over magnesium sulfate, filtered, and concentrated under reduced pressure, delivering the crude product as an orange-brown oil. The crude mixture was purified via silica flash chromatography, eluting with 24% EtOAc in hexanes. The product elutes with R_f = 0.28 in 30% ethyl acetate/hexanes, affording the clean product **Nb-ATRP** as colorless crystals (6.07 g, 58%). The NMR data is in agreement with values reported in the literature[4]. ^{1}H NMR (500 MHz, $CDCl_3$) δ 6.29 (t, *J* = 1.7 Hz, 2H), 4.33 (t, *J* = 5.1 Hz, 2H), 3.81 (t, *J* = 5.6 Hz, 2H), 3.28 (t, *J* = 1.7 Hz, 2H), 2.70 (s, 2H), 1.89 (s, 6H), 1.53 (dt, *J* = 10.1, 1.3 Hz, 1H), 1.35 – 1.29 (m, 1H).

Representative Synthesis of Polystyrene Macromonomer (PS-MM) via ATRP

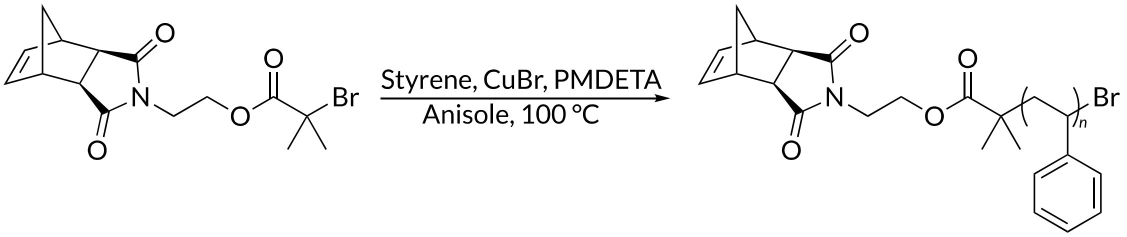

Note: The formula DP = [Styrene] / [Initiator] * Monomer Conversion (%) *was used to calculate theoretical DP for all ATRP reactions reported herein.*

Immediately before starting the reaction, styrene was filtered over a pad of activated basic alumina to remove inhibitor. A 250 mL Schlenk flask was charged with a stirring bar and was flame-dried under high vacuum. CuBr (469 mg, 3.27 mmol, 1.50 eq.) was added to the flask under a stream of nitrogen, and the flask was sealed, evacuated, and refilled with nitrogen 5 times. Norbornene-anchored ATRP initiator (776 mg, 2.18 mmol, 1.0 eq), styrene (27.7 g, 30.4 mL, 266 mmol, 122 eq.), and PMDETA (0.626 mL, 566 mg, 3.27 mmol, 1.50 eq) were added to a vial and dissolved in anisole (30.4 mL; approximately 1:1 volume ratio with respect to styrene). The solution was transferred to the Schlenk flask containing CuBr via syringe, and the green-blue suspension was immediately submerged in a liquid nitrogen bath. The flask was subjected to freeze-pump-thaw cycles until the solution no longer bubbled on thawing (3 cycles) before being backfilled with nitrogen and submerged in a pre-heated 100 °C oil bath. Timepoints were withdrawn immediately before subjecting to heat (t = 0) and hourly thereafter to monitor monomer consumption via ^{1}H NMR spectroscopy (by comparison of the olefinic styrene signal integration to that of the anisole methoxy protons), and the reaction was quenched by opening the suspension to air once the monomer conversion reached ~30% (ca. 2.25 h; target DP = 37). The suspension was filtered over a pad of neutral alumina, the plug was rinsed with DCM (200 mL), and the filtrate was concentrated under reduced pressure. The oily residue was dissolved in a minimal amount of THF (ca. 15 – 30 mL) and pipetted into methanol (350 mL) at –78 °C to precipitate the polymer. The precipitate was collected via suction filtration over a medium sintered glass funnel and redissolved in minimal THF. This process was repeated for a total of 4 precipitations, and the collected precipitate was dried under high vacuum overnight, delivering the **PS-MM** as a very fine colorless powder (2.62 g, 26%). The dried material was redissolved in chloroform and filtered through a 0.2 μm syringe filter for GPC-MALS analysis: M_n = 4.4 kDa, $Đ$ = 1.03, *dn/dc* (estimated via 100% mass recovery) = 0.1314. ^{1}H NMR (500 MHz, $CDCl_3$) δ 7.26 – 6.88 (br, 100H), 6.83 – 6.31 (br, 66H), 6.27 (s, 2H, norbornene double bond), 2.10 – 1.71 (br, 40H), 1.51 – 1.13 (br, 93H). NMR data is in agreement with the literature values[4].

Synthesis of Metathesis Initiators:

Synthesis of Grubbs' 3rd generation catalyst (G3):

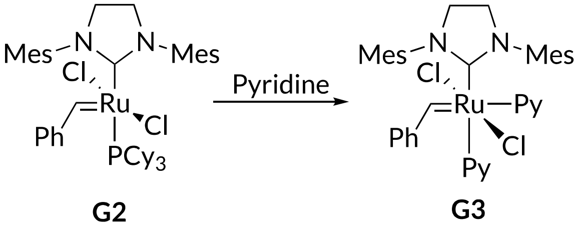

Grubbs' 2nd generation catalyst (**G2**; 51.3 mg, 0.06 mmol, 1.00 eq.) was weighed into an oven-dried vial containing an oven-dried stirring bar. The vial was cycled into a nitrogen filled glovebox, and the G2 was suspended in anhydrous pyridine (200 µL, 196 mg, 2.47 mmol, 40.9 eq.). The deep green suspension was stirred at RT for 15 minutes, and the vial was cycled out of the glovebox. Ice cold pentane (~1 mL) was added to the to the suspension, which was subsequently centrifuged at 3000 RPM at room temperature for 15 minutes. The pentane was removed via pipette, and the process of suspending in ice cold pentane, centrifuging, and decanting was repeated for a total of three cycles. The residue was dried under vacuum, delivering the product **G3** as a fine green powder (40.4 mg, 92%). NMR data is in accordance with the literature values[6]. ^{1}H NMR (500 MHz, C_6D_6) δ 19.62 (s, 1H), 8.58 (br. s, 2H), 8.32 (br. s, 2H), 8.01 (d, *J* = 6.7 Hz, 2H), 7.20 (t, *J* = 7.4 Hz, 1H), 6.96 – 6.05 (br., multiple peaks, 9H), 3.42 (br. d, 4H), 2.85 (br. s, 6H), 2.42 (br. s, 6H), 2.14 (br. s, 6H).

CB6 Overall Synthesis:

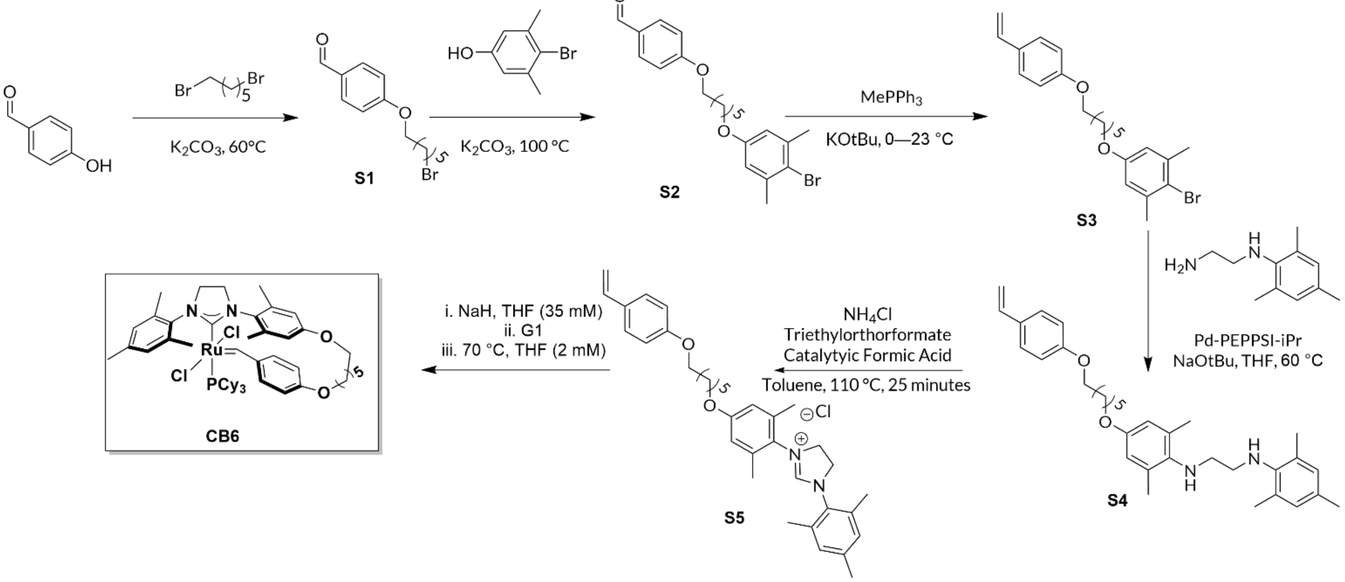

Scheme S1: Overall synthetic scheme for the preparation of **CB6**

Synthesis of 4-((6-bromohexyl)oxy)benzaldehyde (S1)

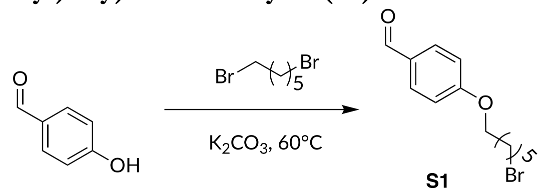

To a round bottom flask equipped with a stirring bar was added 4-hydroxybenzaldehyde (5.00 g, 40.9 mmol, 1.00 eq.), 1,6-dibromohexane (18.9 mL, 20.0 g, 81.9 mmol, 2.00 eq.), potassium carbonate (11.3 g, 81.9 mmol, 2.00 eq.), and acetone (100 mL; 0.409 M in 4-hydroxyaldehyde). The pink suspension was heated to 60 °C and refluxed for 2 d. After refluxing 2 d, the reaction was cooled to room temperature and the beige-pink suspension was filtered over celite. The residue was rinsed with ethyl acetate (50 mL), and the filtrate was partitioned into DI water (50 mL). The biphasic mixture was added to a separatory funnel, the organic phase separated, and the aqueous phase was extracted with ethyl acetate (3 x 50 mL). The combined organic phase was washed with water (3 x 50 mL) and brine (1 x 100 mL). The organic layer was then dried over sodium sulfate, filtered, and concentrated under vacuum. The crude yellow oil was purified by flash chromatography using 20% ethyl acetate in hexanes as the mobile phase. The product was eluted with an R_f of 0.34, delivering **S1** as a colorless solid (5.60 g, 48%). ^{1}H NMR (500 MHz, $CDCl_3$) δ 9.87 (s, 1H), 7.82 (d, *J* = 8.7 Hz, 2H), 6.98 (d, *J* = 8.7 Hz, 2H), 4.04 (t, *J* = 6.4 Hz, 2H), 3.42 (t, *J* = 6.6 Hz, 2H), 1.98 – 1.77 (m, 4H), 1.56 – 1.46 (m, 4H). ^{13}C NMR (126 MHz, $CDCl_3$) δ 190.91, 164.26, 132.11, 129.95, 114.85, 68.25, 33.84, 32.72, 29.01, 27.97.

Synthesis of 4-((6-(4-bromo-3,5-dimethylphenoxy)hexyl)oxy)benzaldehyde (S2)

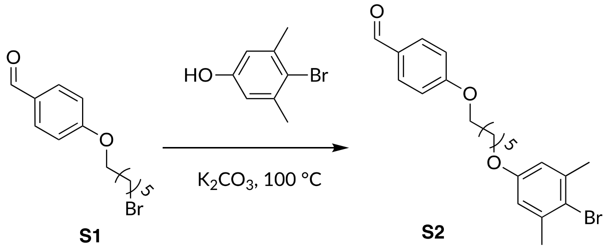

To a round bottom flask equipped with a stirring bar was added **S1** (3.74 g, 13.1 mmol, 1.00 eq.), 4-bromo-3,5-dimethylphenol (3.16 g, 15.7 mmol, 1.20 eq.), and potassium carbonate (3.08 g, 22.3 mmol, 1.70 eq.). The same flask was charged with DMF (120 mL; 0.109 M in **S1**) and the pink suspension was stirred at 100 °C for 24 h. The reaction was cooled to room temperature, filtered over celite, and the residue rinsed with ethyl acetate (50 mL). The filtrate was reduced i*n vacuo* at 60 °C., and the crude oil was redissolved in ethyl acetate (25 mL) and washed with 5% aqueous lithium chloride (5 x 50 mL), DI water (5 x 50 mL), and brine (2 x 50 mL). The organic layer was dried over sodium sulfate, filtered, and concentrated under vacuum. The dark red crude oil was purified via silica gel flash chromatography, eluting with 20% ethyl acetate in hexanes. The product **S2** was eluted as a colorless, slightly yellow solid with R_f = 0.36 (3.79 g. 71%). *Note: In some cases, there is a slightly lower spot which coelutes with the product in this solvent mixture (appearing as one spot on TLC). The material can be carried forward to the next step with this impurity present, or the impurity can be removed by silica gel flash chromatography eluting with 100% DCM, in which case the product* ***S2*** *elutes with* R_f *= 0.44 and the impurity elutes with* R_f *= 0.28.* ^{1}H NMR (500 MHz, $CDCl_3$) δ 9.88 (s, 1H), 7.81 (d, *J* = 8.7 Hz, 2H), 6.98 (d, *J* = 8.7 Hz, 2H), 6.64 (s, 2H), 4.05 (t, *J* = 6.6 Hz, 2H), 3.92 (t, *J* = 6.6 Hz, 2H), 2.37 (s, 6H), 1.90 – 1.76 (overlap, 4H), 1.59 – 1.50 (overlap, 4H). ^{13}C NMR (126 MHz, $CDCl_3$) δ 190.91, 164.30, 157.67, 139.17, 132.11, 129.94, 118.19, 114.86, 114.52, 68.35, 67.93, 29.29, 29.13, 25.94, 25.89, 24.17.

Synthesis of 2-bromo-1,3-dimethyl-5-((6-(4-vinylphenoxy)hexyl)oxy)benzene (S3)

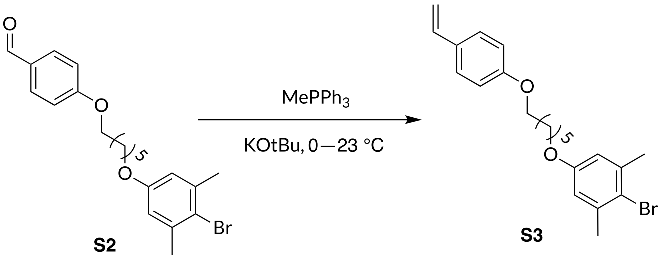

S2 **S3**

A Schlenk flask equipped with a stirring bar was flame dried and charged with methyltriphenylphosphonium bromide (3.51 g, 9.81 mmol, 1.20 eq.) and potassium *tert*-butoxide (1.85 g, 16.4 mmol, 2.00 eq.). The flask was evacuated and refilled with nitrogen three times, and the solids were suspended into dry THF (50 mL, 0.196 M in methyltriphenylphosphonium bromide). The resulting bright yellow suspension was cooled to 0 °C and stirred for 15 minutes. Simultaneously, **S2** (3.31 g, 8.18 mmol, 1.00 eq) was added to a flame-dried round bottomed flask and dissolved in 60 mL dry THF (0.136 M in **S2**). The **S2** solution was added to the stirring phosphorous ylide suspension at 0 °C via cannula transfer, and the resulting beige-orange suspension was stirred at 0 °C for 15 minutes then at RT for 24 hours. The resulting orange suspension was quenched with 60 mL methanol and filtered over celite, and the residue was rinsed with 150 mL DCM. The solvents were removed *in vacuo*, and the residue redissolved in 150 mL DCM. The organic phase was washed with water (3 x 75 mL) and brine (1 x 150 mL), dried over sodium sulfate, filtered, and concentrated to deliver the crude material as a beige oil. The crude oil was purified by flash chromatography using a hexanes-DCM gradient, where **S3** eluted as a colorless solid with R_f = 0.38 in 40% DCM/Hexanes (2.41 g, 73%). ^{1}H NMR (500 MHz, $CDCl_3$) δ 7.34 (d, *J* = 8.4 Hz, 2H), 6.86 (d, *J* = 8.4 Hz, 2H), 6.71 – 6.62 (m, 3H), 5.61 (d, *J* = 17.4 Hz, 1H), 5.13 (d, *J* = 10.8 Hz, 1H), 3.98 (t, *J* = 6.4 Hz, 2H), 3.92 (t, *J* = 6.4 Hz, 2H), 2.38 (s, 6H), 1.87 – 1.76 (overlap, 4H), 1.58 – 1.49 (overlap, 4H). ^{13}C NMR (126 MHz, $CDCl_3$) δ 159.02, 157.72, 139.17, 136.40, 130.44, 127.50, 118.17, 114.62, 114.56, 111.59, 68.01, 67.98, 29.33, 25.98, 24.18.

Synthesis of *N*-mesitylethane-1,2-diamine•HBr salt (Mes-diamine•HBr)

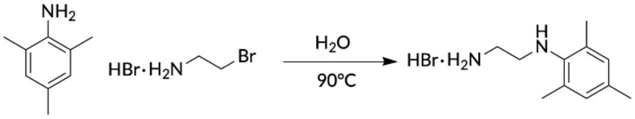

To a round bottom flask equipped with a stirring bar was added 2,4,6-trimethyl aniline (30.0 g, 222 mmol, 2.00 eq.) and 2-bromoethylamine hydrobromide (22.7 g, 111 mmol, 1.00 eq.). The solids were dissolved in H_2O (30 mL; 3.70 M in bromoethylamine hydrobromide). The flask was equipped with a condenser and the mixture was stirred at 90 °C for 12 hours. After stirring, the solution turned pinkish-purple and slowly turned dark red. After 12 hours, the solution was cooled to RT and washed with ethyl acetate (5 x 30 mL). The dark red aqueous layer was collected and concentrated under vacuum to deliver the crude product as a light brown solid. The residue was recrystallized from 1:1 EtOAc/methanol (required ca. 200 mL at reflux to dissolve all the crude). The resulting crystals were collected by vacuum filtration after one day in the freezer and washed with ice cold ethyl acetate (ca. 300 mL) until colorless to yield **Mes-diamine•HBr** as a colorless solid (22.0 g, 78%). NMR data is consistent with literature values[7]. ^{1}H NMR (500 MHz, MeOD) δ 7.06 (s, 2H), 3.65 – 3.58 (m, 2H), 3.48 – 3.41 (m, 2H), 2.47 (s, 6H), 2.30 (s, 3H). ^{13}C NMR (126 MHz, MeOD) δ 140.78, 132.66, 132.06, 47.93, 36.93, 20.77, 18.05

Synthesis of *N*-mesitylethane-1,2-diamine freebase (Mes-diamine)

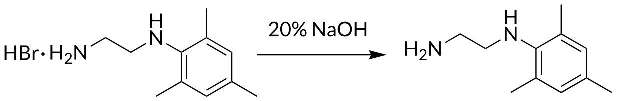

To a round bottom flask equipped with a stirring bar was added **Mes-diamine•HBr** (2.10 g, 8.10 mmol, 1.00 eq.) and 20% aq. NaOH (60 mL; 12.6 g NaOH, 315 mmol, 39.0 eq.). The solution was stirred for 30 min at room temperature. The solution was extracted with DCM (3 x 40 mL) and the combined organic phase was washed with water (3 x 25 mL) and brine (1 x 25 mL). The organic phase was dried over sodium sulfate and concentrated under vacuum for one hour to yield **Mes-diamine** as a brown-yellow oil (0.981 g, 68%). ^{1}H NMR (500 MHz, $CDCl_3$) δ 6.84 (s, 2H), 3.02 – 2.96 (m, 2H), 2.94 – 2.88 (m, 2H), 2.30 (s, 6H), 2.25 (s, 3H); ^{13}C NMR (126 MHz, $CDCl_3$) δ 143.59, 131.23, 129.78, 129.45, 51.34, 42.63, 20.58, 18.39.

Synthesis of *N*-(2,6-dimethyl-4-(4-(4-vinylphenoxy)butoxy)phenyl)-*N*-(3,4,5-trimethylphenyl)ethane-1,2-diamine (S4)

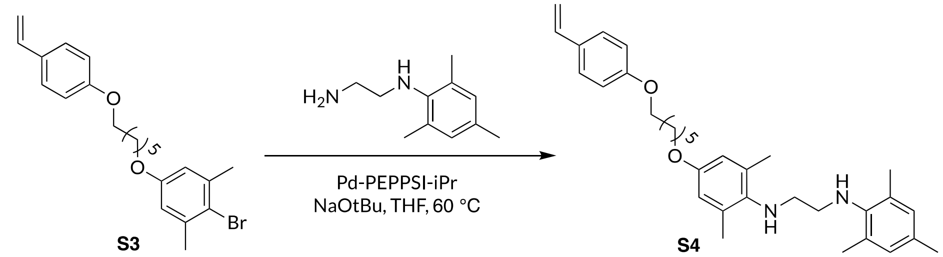

To a flame-dried Schlenk flask with stirring bar was added **S3** (5.159 g, 12.8 mmol, 1.00 eq), sodium *tert*-butoxide (1.844 g, 19.2 mmol, 1.50 eq), and Pd-PEPPSI-Ipr (1.043 g, 0.150 mmol, 12 mole%). The flask was evacuated and refilled with nitrogen 3 times, and the solids were suspended in 25 mL of anhydrous THF. **Mes-diamine** (3.192 g, 17.9 mmol,1.40 eq.) was dissolved in 25 mL of THF and added via syringe to the **S3** solution. The diamine flask was rinsed with 25 mL of anhydrous THF and added to the stirring **S3** solution, bringing the total volume to 75 mL THF (0.17 M in **S3**). The dark red solution was submerged into an oil bath which had been preheated to 60 °C and stirred at this temperature for 16 hours. After stirring overnight, the suspension became a very dark purple-ish red and 300 mg of SilaMet Triamine resin was added to the mixture. The suspension was stirred with the resin for 3 hours at RT, then was filtered over a pad of neutral alumina. The residue was rinsed with 100 mL DCM, and the crude suspension was concentrated under reduced pressure to deliver the crude product as a very dark red oil. This oily residue was purified via flash chromatography using a gradient from 0 – 40% ethyl acetate in hexanes. The product **S4** eluted off the column (R_f = 0.39 in 40% EtOAc/Hexanes) as a brown oil which slowly crystallized into a beige-yellow solid (4.229 g, 66%). ^{1}H NMR (500 MHz, $CDCl_3$) δ 7.34 (t, *J* = 4.3 Hz, 2H), 6.90 – 6.83 (m, overlapping peaks, 4H), 6.67 (dd, *J* = 17.4, 10.8 Hz, 1H), 6.60 (s, 2H), 5.62 (d, *J* = 17.6 Hz, 1H), 5.13 (d, *J* = 10.9 Hz, 1H), 3.98 (t, *J* = 7.0 Hz, 2H), 3.92 (t, *J* = 6.3 Hz, 2H), 3.22 (br, 2H), 3.19 – 3.07 (overlap, 4H), 2.30 (s, 12H), 2.25 (s, 3H), 1.87 – 1.77 (overlap, 4H), 1.58 – 1.51 (m, overlapping peaks, 4H). ^{13}C NMR (126 MHz, $CDCl_3$) δ 159.04, 154.54, 143.59, 139.17, 136.41, 132.09, 131.48, 130.40, 129.78, 129.63, 127.49, 114.76, 114.62, 111.55, 68.04, 68.02, 49.65, 49.33, 29.49, 29.35, 26.04, 26.04, 26.01, 20.67, 18.73, 18.60.

Synthesis of 3-(2,6-dimethyl-4-(4-(4-vinylphenoxy)butoxy)phenyl)-1-mesityl-4,5-dihydro-imidazol-3-ium salt (S5)

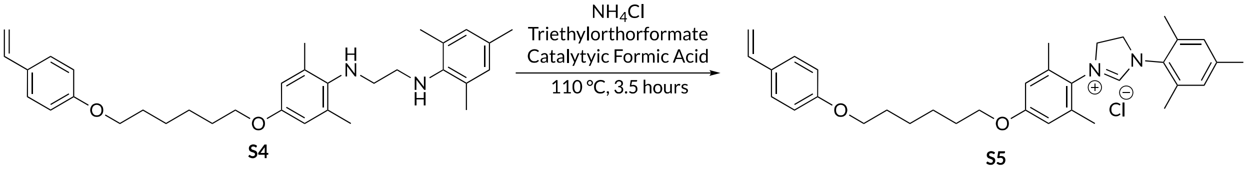

To a flame-dried 10-mL Schlenk flask with a stirring bar was added **S4** (0.310 g, 0.619 mmol, 1.00 eq.) and ammonium chloride (66.2 mg, 1.24 mmol, 2.00 eq.) under nitrogen. The flask was evacuated and backfilled with nitrogen 3 times, and 2 drops of formic acid (88% aqueous solution) and triethyl orthoformate (3.74 mL, 3.33 g, 22.5 mmol, 36.3 eq.) were added to the vessel. The solution was stirred at 110 °C for a further 3.5 hours, then was cooled to room temperature and concentrated under vacuum. The resulting pale yellow solid was loaded onto a silica column and the product was eluted with a gradient from 5% to 10% methanol in DCM. The product **S5** was collected as a beige solid, eluting with R_f = 0.37 in 10% methanol in DCM (293 mg, 82%). ^{1}H NMR (500 MHz, $CDCl_3$) δ 9.24 (s, 1H), 7.32 (d, *J* = 4.1 Hz, 2H), 6.94 (s, 2H), 6.83 (d, *J* = 4.6 Hz, 2H), 6.68 – 6.59 (overlap, 3H), 5.58 (d, *J* = 9.1 Hz, 1H), 5.10 (t, *J* = 4.5 Hz, 1H), 4.56 (s, 4H), 3.99 – 3.90 (overlap, 4H), 2.38 (overlap, 12H), 2.28 (s, 3H), 1.85 – 1.75 (overlap, 4H), 1.57 – 1.47 (m, overlapping peaks, 4H); ^{13}C NMR (126 MHz, $CDCl_3$) δ 160.26, 160.01, 159.00, 140.71, 136.87, 136.35, 135.09, 130.40, 130.32, 130.19, 127.47, 125.48, 115.05, 114.60, 111.58, 68.14, 67.95, 52.27, 52.09, 29.29, 29.16, 25.92, 25.90, 21.18, 18.53, 18.15.

Synthesis of CB6

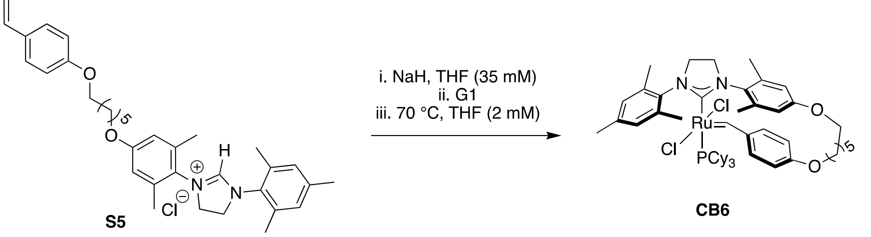

S5

CB6

To a dry 20 mL vial charged with a stirring bar in a nitrogen filled glovebox, **S5** (0.100 g, 0.183 mmol, 1.00 eq) and sodium hydride (0.110 g, 4.57 mmol, 25.0 eq.) were added. The solids were suspended in anhydrous THF (5.20 mL; 35.0 mM in **S5**), and the mixture was left to stir at room temperature overnight. The next day, Grubbs 1st generation catalyst (G1) (0.150 g, 0.183 mmol, 1.00 eq.) was added to the reaction mixture, which was stirred at room temperature for 90 min. After 90 min, the reaction mixture was cycled out of the box, transferred to a round-bottomed flask, and diluted with THF (91.4 mL; 2.00 mM in **S5**). The diluted reaction mixture was submerged in a pre-heated 70 °C oil bath and stirred for 3 h. The reaction was cooled to room temperature, filtered through celite, and concentrated under vacuum. The resulting crude mixture was triturated using a sonication bath with pentane. The suspension was then centrifuge at 10 °C at 3000 rpm for 10 min followed by decanting of the solvent. This process was repeated for a total of 3 triturations. The collected light pink solid was dried with a nitrogen flow to yield **CB6** mixed with dimer **bis-CB6** in an 8:1 ratio (0.141 g, 82%).

Optional additional purification by preparative gel permeation chromatography (prep-GPC)
The crude mixture (without trituration) can be directly purified via prep-GPC. The mixture was dissolved in HPLC grade chloroform (stabilized with 0.5-1% EtOH) to a concentration of ~100 mg/mL, filtered through an Aura MT 0.45 µm syringe filter, and purified via recycling prep-GPC (RI detection only). Removal of solvent under reduced pressure afforded analytically pure **CB6** and **bis-CB6**. Both fractions were further sonicated in diethyl ether followed by pentane to completely dry the material. See **Figure S1** below for representative prep-GPC chromatogram. All polymerizations reported herein were performed using prep-GPC purified **CB6**.

Characterization Data for pGPC isolated ***CB6***:
^{1}H NMR (500 MHz, C_6D_6): δ(ppm) 19.40 (s, 1H), 9.39 (s, 1H), 7.33 (s, 1H), 6.93 (m, 2H), 6.83 (s, 1H), 6.48 (m, 2H), 5.91 (s,1H), 3.99 (s, 1H), 3.66 (s, 1H), 3.42-3.17 (m, 6H), 2.96 (s, 3H), 2.79 (s, 3H), 2.63 (s, 3H), 2.55 (m, 4H), 2.43 (s, 3H), 2.21 (s, 3H), 1.78 (s, 3H), 1.69-1.55 (m, 16H) 1.39 (s, 3H), 1.25-1.05 (m, 19H); ^{13}C NMR (125 MHz, C_6D_6): δ(ppm) 222.32 (J_{CP} = 78.6 Hz), 158.81, 158.54, 147.53, 139.50, 138.26, 135.93, 135.38, 133.63, 132.72, 130.38, 130.19, 117.13, 114.72, 113.67, 109.46, 66.18, 65.03, 52.13, 51.61, 34.44, 32.16 (J_{CP} = 16.0 Hz), 29.89, 29.53, 28.24 (J_{CP} = 9.5 Hz), 27.91, 26.67, 24.26, 22.72, 22.42, 21.23, 20.57, 19.57, 19.13.

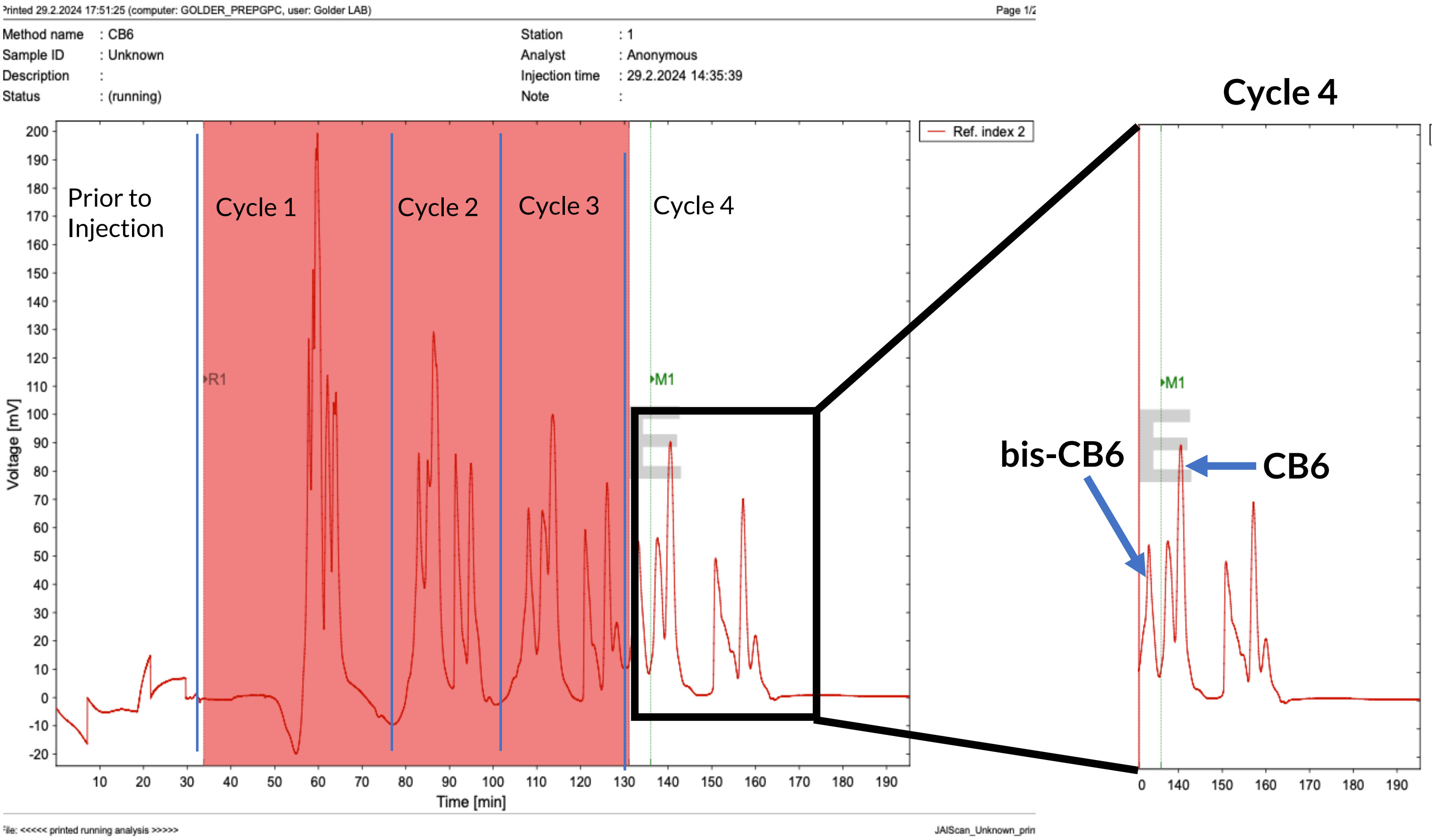

Figure S1. Representative differential refractive index trace of prep-GPC purification of **CB6** and **bis-CB6** from crude mixture. Separation was achieved on the fourth cycle through the size exclusion columns (represented in the zoomed in region). The dimer and CB6 peaks are indicated with arrows.

Synthesis of Bottlebrush Polymers:
Note: The formula DP = [Initiator]/[Monomer] *was used to calculate theoretical DP for all ROMP and REMP reactions reported herein. All REMP reactions were performed using* ***prep-GPC purified CB6.***

Synthesis of Linear PDMS Bottlebrush Polymer via ROMP (Theoretical DP = 30):

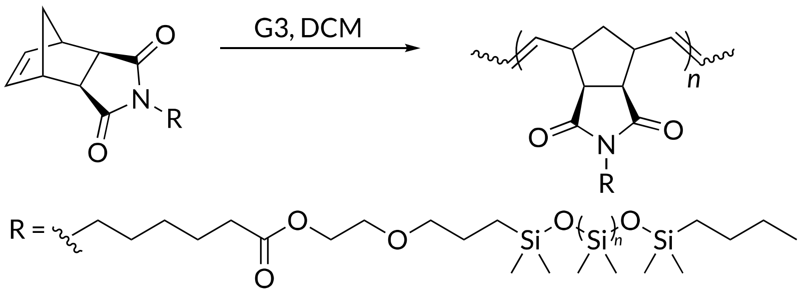

PDMS-MM (M_n = 6.3 kDa, 84.2 mg, 14.5 μmol, 30.0 eq. relative to G3) and G3 (0.80 mg, 1.10 μmol) were weighed into oven-dried 2 mL vials charged with oven-dried stirring bars. The vials were cycled into a nitrogen-filled glovebox, and **PDMS-MM** was taken up in 688 μL of anhydrous DCM. The G3 was taken up in 80.0 μL of anhydrous DCM (10.0 mg/mL, 13.8 mM), and the solutions were stirred at RT for 2 minutes until homogenous. The G3 stock solution (35.1 μL, 0.483 μmol G3, 1.00 eq.) was dispensed into the PDMS solution (bringing the total monomer solution to 20.0 mM = 116 mg **PDMS-MM**/mL), and the solution was stirred at RT for 2 hours. The slightly yellow solution was cycled out of the glovebox, diluted into 1 mL THF, and quenched with 5 drops of ethyl vinyl ether. A small amount (around a spatula tip) of SilaMetTAAcOH Resin was added to the vial. The suspension was shaken vigorously before being filtered over a short plug of celite. The residue was rinsed with 2 mL THF, and the filtrate concentrated *in vacuo*, delivering the product as a highly viscous, slightly beige oil (48.3 mg, 57%). The dried oil was redissolved in chloroform and filtered through a 0.2 μm syringe filter for GPC-MALS analysis: M_n = 350 kDa (DP = 56), $Đ$ =1.1, *dn*/*dc* (measured via batch injection) = –0.0177

Synthesis of Cyclic PDMS Bottlebrush Polymer via REMP (Theoretical DP = 30):

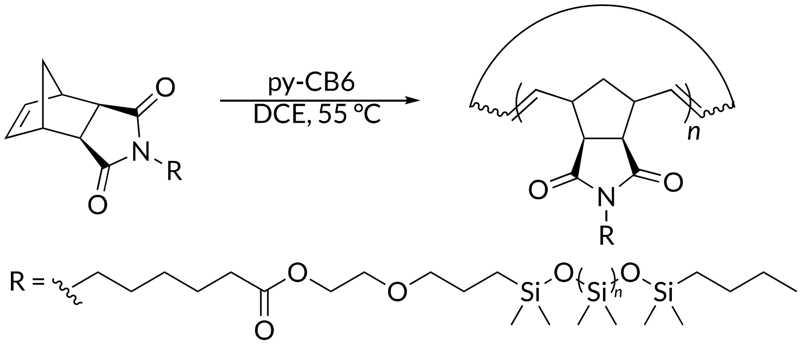

PDMS-MM (M_n = 6.3 kDa, 124 mg, 21.3 μmol, 30.0 eq relative to CB6) and CB6 (1.70 mg, 1.79 μmol) were weighed into oven-dried 2 mL vials charged with oven-dried stirring bars. The vials were cycled into a nitrogen-filled glovebox, and the PDMS macromonomer was taken up in 994 μL of anhydrous DCE. The CB6 was dissolved in 170 μL of anhydrous DCE, and 4.62 μL of anhydrous pyridine (4.50 mg, 57.4 μmol, 32.0 eq. relative to CB6) was added to the CB6 solution (bringing the total CB6 solution to 10.3 mM) and the solutions were stirred at RT for 2 minutes until homogenous. The pyr-CB6 stock solution (69.0 μL, 0.709 μmol CB6, 1.00 eq.) was added to the PDMS solution (bringing the total monomer solution to 20.0 mM = 116 mg **PDMS-MM**/mL), and the reaction mixture was added to a pie block which had been preheated to 55 °C. The reaction was stirred at this temperature for 2 hours before being cycled out of the glovebox, where it was diluted into 1mL THF. A small amount (around a spatula tip) of SilaMetTAAcOH Resin was added to the vial, and the suspension was shaken vigorously before being filtered over a short plug of celite. The residue was rinsed with 2 mL THF, and the filtrate was portioned roughly in half between two tared vials. To one of these vials was added 5 drops of ethyl vinyl ether, and both vials were dried under reduced pressure to deliver highly viscous, slightly beige oils (103 mg total between the quenched and unquenched vials, 83%). The dried oils were redissolved in chloroform and filtered through 0.2 μm syringe filters for GPC-MALS analysis: M_n = 720 kDa (DP = 114), $Đ$ = 1.1, *dn/dc* (measured via batch injection) = –0.0115

Synthesis of Linear PS Bottlebrush Polymer via ROMP (Theoretical DP = 50):

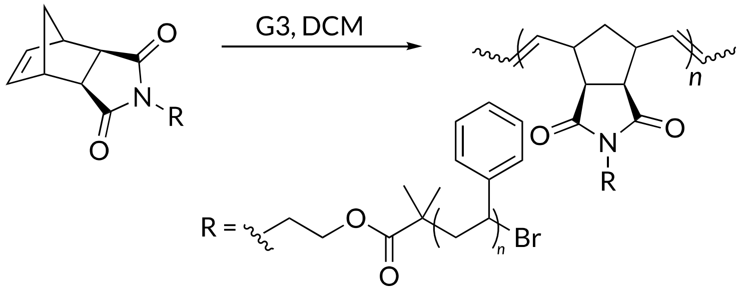

PS-MM (M_n = 4.6 kDa, 21.4 mg, 4.70 μmol, 50.0 eq. relative to G3) and G3 (1.20 mg, 1.65 μmol) were weighed into oven-dried 2 mL vials charged with oven-dried stirring bars. The vials were cycled into a nitrogen-filled glovebox, and the PS macromonomer was taken up in 226.40 μL of anhydrous DCM. The G3 was taken up in 120 μL of anhydrous DCM (10 mg/mL, 13.8 mM), and the solutions were stirred at RT for 2 minutes until homogenous. The G3 stock solution (6.55 μL, 0.090 μmol G3, 1.0 eq.) was dispensed to the PS solution (bringing the total monomer solution to 20.0 mM = 92 mg **PS-MM**/mL), and the solution was stirred at RT for 2 hours. The colorless solution was cycled out of the glovebox and diluted into 1 mL DCM, and 5 drops of ethyl vinyl ether was added to the solution. The solution was filtered over a short plug of neutral alumina, and the plug was rinsed with 2 mL DCM. The DCM was removed under reduced pressure, and ice-cold pentane was added to the resulting colorless oil to precipitate the product as a fine colorless powder. The pentane was removed under reduced pressure and the residue dried under vacuum to deliver the dried product as a fine, colorless powder (22.3 mg, 74%). The dried polymers were redissolved in chloroform and filtered through 0.2 μm syringe filters for GPC-MALS analyses: M_n = 110 k/Da (DP = 25), $Đ$ = 1.1, *dn/dc* (measured via batch injection) = 0.1706

NOTE: FOR LARGER PS BRUSHES (PS-MM > 8000 g/mol), MORE DILUTE CONDITIONS (40 mg/mL) WERE REQUIRED TO AFFORD CONTROLLED POLYMERIZATIONS IN BOTH ROMP AND REMP.

Synthesis of Cyclic PS Bottlebrush Polymer via REMP (Theoretical DP = 20):

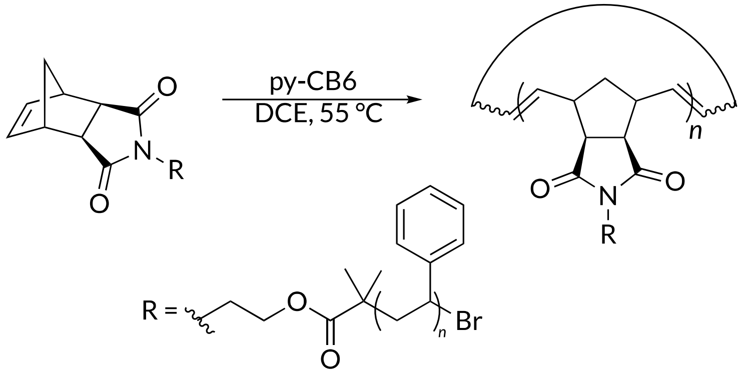

PS-MM (M_n = 4.6 kDa, 25.6 mg, 5.60 μmol, 20.0 eq. relative to CB6) and CB6 (1.90 mg, 2.00 μmol) were weighed into oven-dried 2 mL vials charged with oven-dried stirring bars. The vials were cycled into a nitrogen-filled glovebox, and the PS macromonomer was taken up in 252 μL of anhydrous DCE. The CB6 was dissolved in 190 μL of anhydrous DCE, and 5.17 μL of anhydrous pyridine (5.10 mg, 64.1 μmol, 32.0 eq. relative to CB6) was added to the CB6 solution (bringing the total CB6 solution to 10.3 mM) and the solutions were stirred at RT for 2 minutes until homogenous. The pyr-CB6 stock solution (27.2 μL, 0.279 μmol CB6, 1.00 eq.) was added to the PS solution (bringing the reaction mixture to a total concentration of 20.0 mM = 92.0 mg/mL with respect to the macromonomer), and the reaction mixture was added to a pie block which had been preheated to 55 °C. The reaction was stirred at this temperature for 3.5 hours before being cycled out of the glovebox, where it was diluted into 1 mL DCE. The solution was filtered over a short plug of neutral alumina, and the plug was rinsed with 2 mL DCE. The filtrate was portioned roughly in half between two tared vials. To one of these vials was added 5 drops of ethyl vinyl ether, and both vials were dried under reduced pressure. Ice cold pentane was added to the resulting colorless oil to precipitate the product as a fine colorless powder. The pentane was removed under reduced pressure and the residue dried under vacuum to deliver the dried product as a fine, colorless powder (22.3 mg total between the quenched and unquenched vials, 87%). The dried polymers were redissolved in chloroform and filtered through 0.2 μm syringe filters for GPC-MALS analyses: M_n = 130 kDa (DP = 27), $Đ$ = 1.1, *dn/dc* (measured via batch injection) = 0.1119

NOTE: FOR LARGER PS BRUSHES (PS-MM > 7500 g/mol), MORE DILUTE CONDITIONS (40 mg/mL) WERE REQUIRED TO AFFORD CONTROLLED POLYMERIZATIONS IN BOTH ROMP AND REMP.

3: NMR Spectroscopy Data:

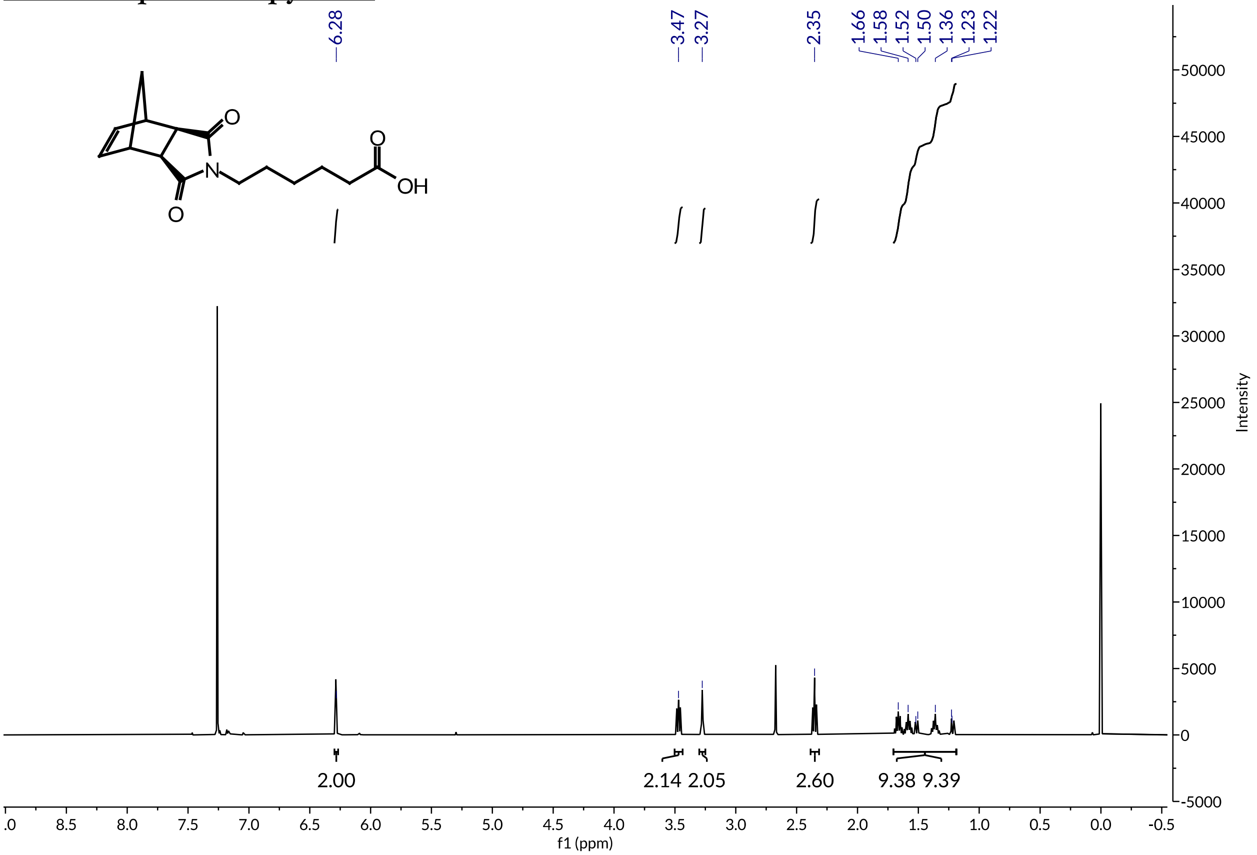

Figure S2: ^{1}H NMR ($CDCl_3$, 500 MHz) **of *N*-(hexanoic acid)-*cis*-5-norbornene-*exo*-dicarboximide (Nb-COOH)**

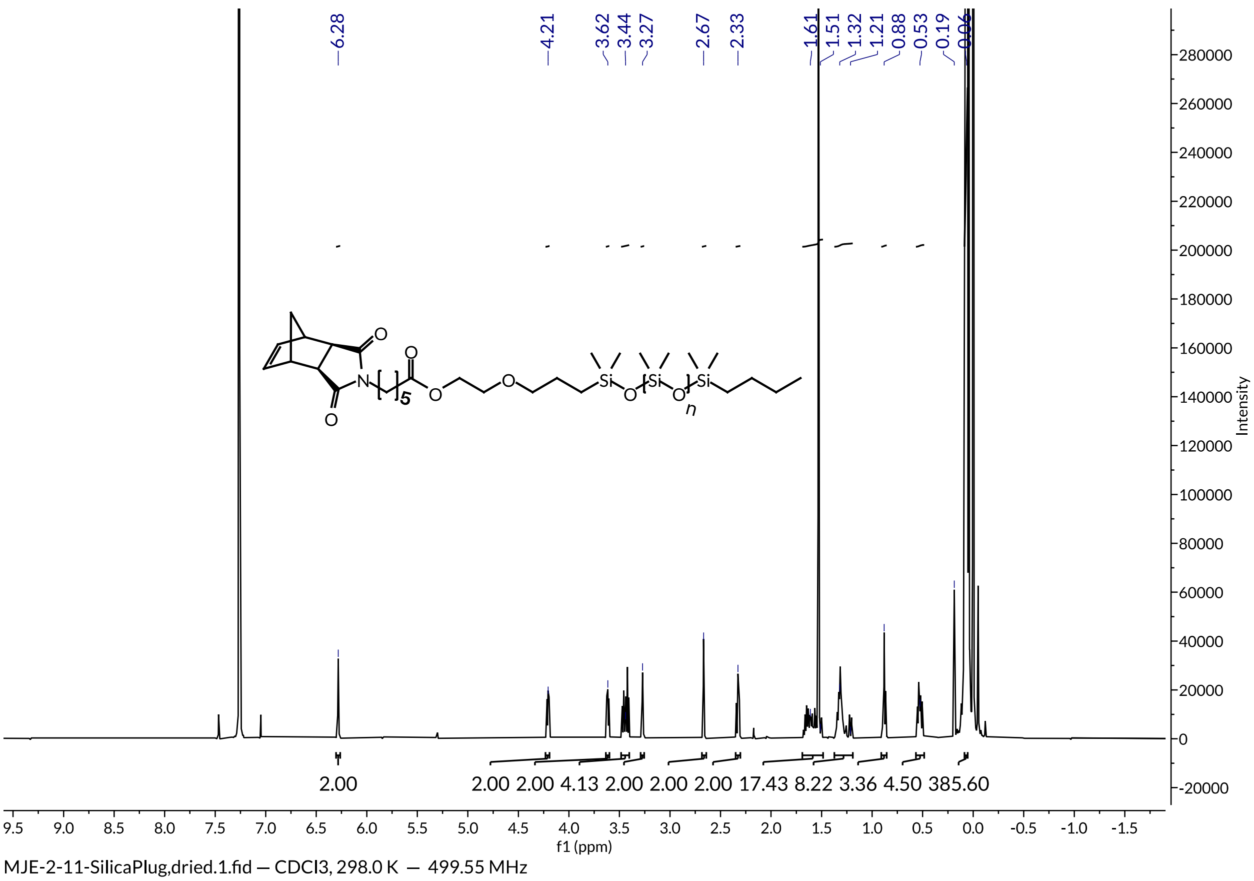

Figure S3: ^{1}H NMR ($CDCl_3$, 500 MHz) of **PDMS Macromonomer (PDMS-MM)**

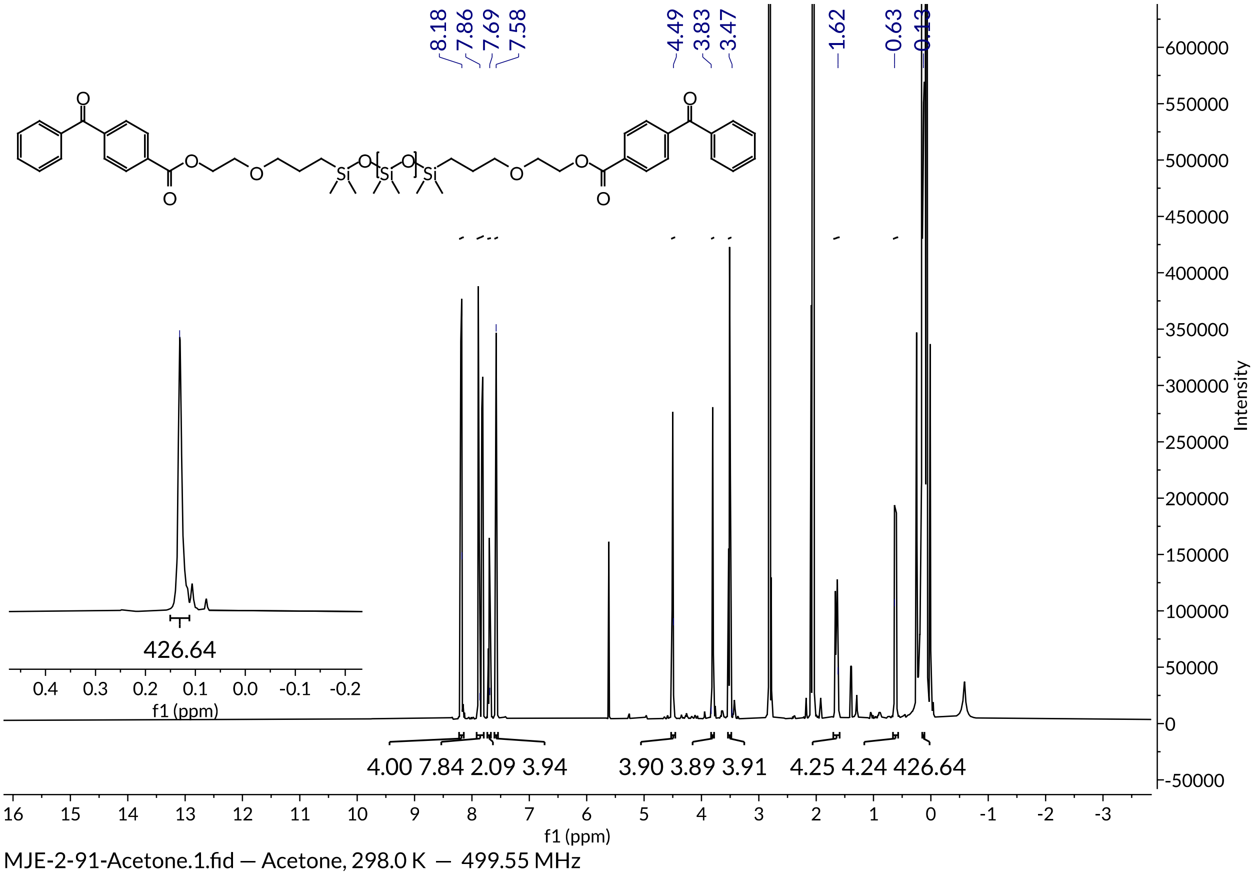

Figure S4: ^{1}H NMR (Acetone, 500 MHz) of **PDMS Crosslinker (BisBP-PDMS)**

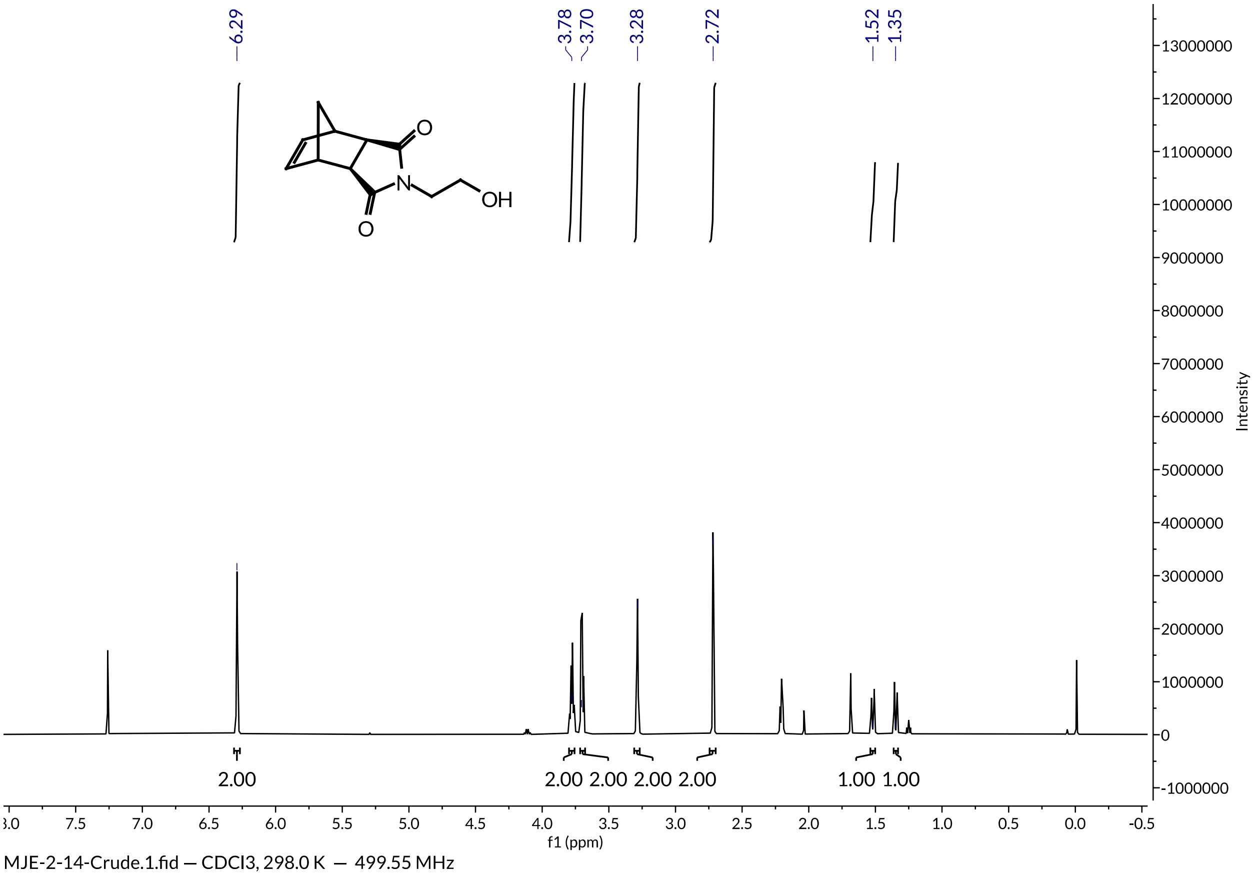

Figure S5: ^{1}H NMR ($CDCl_3$, 500 MHz) of ***N*-(ethanol)-*cis*-5-norbornene-*exo*-dicarboximide (Nb-OH)**

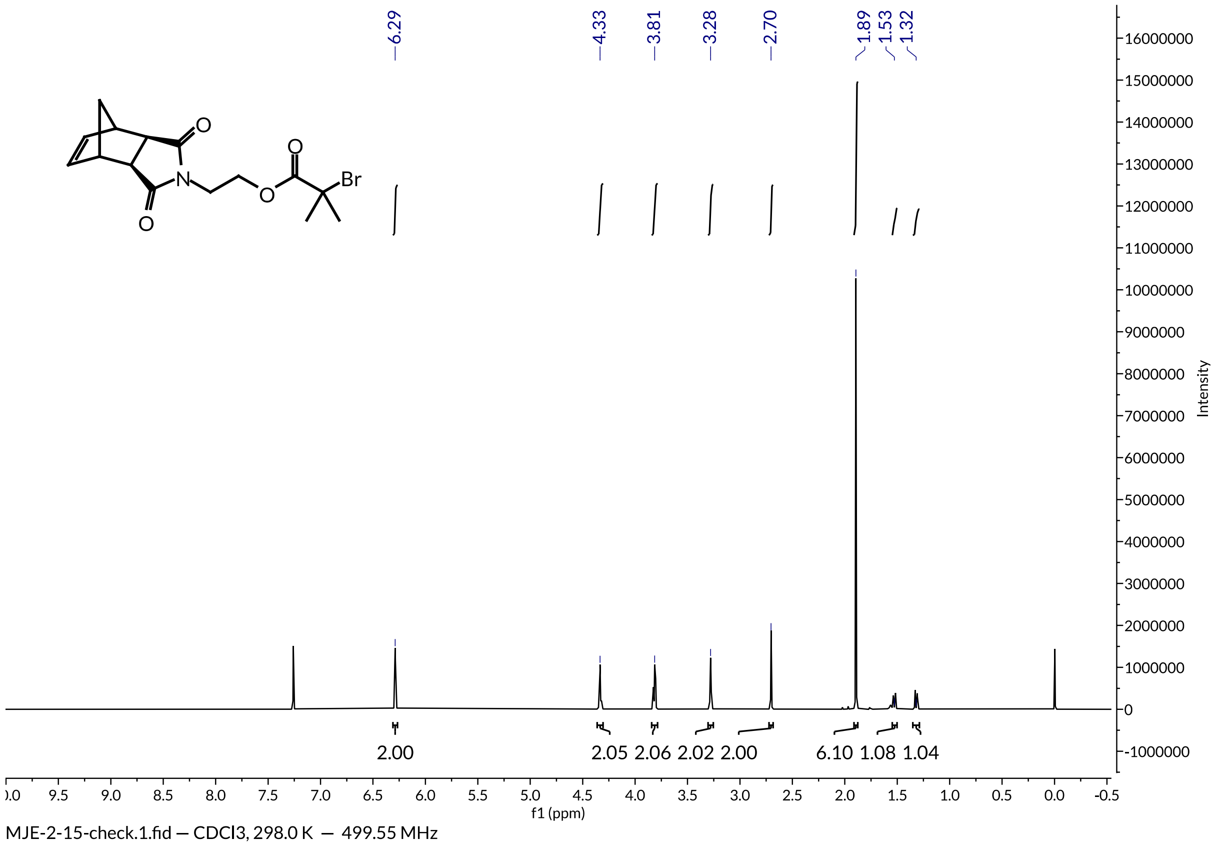

Figure S6: ^{1}H NMR ($CDCl_3$, 500 MHz) of **Norbornene-anchored ATRP initiator (Nb-ATRP)**

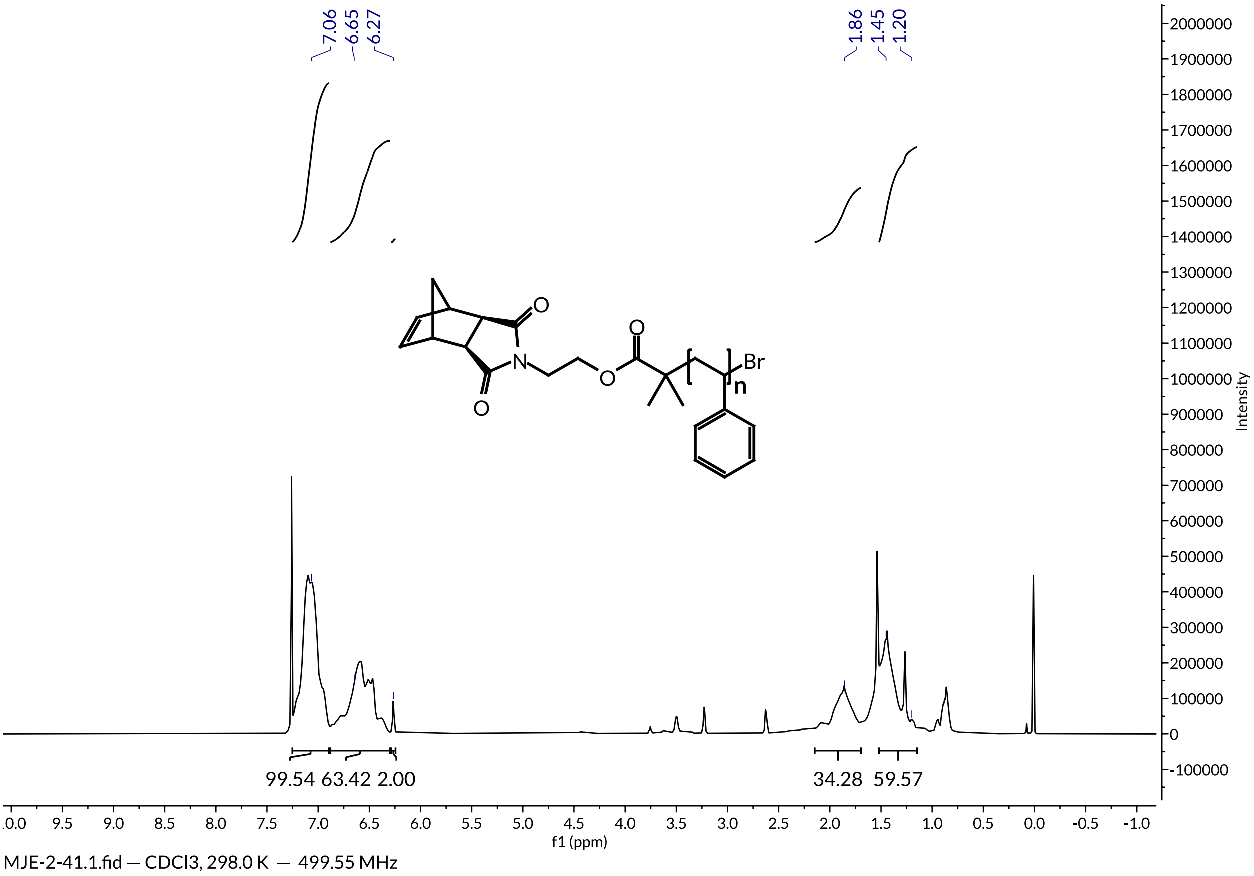

Figure S7: ^{1}H NMR ($CDCl_3$, 500 MHz) of **PS Macromonomer (PS-MM)**

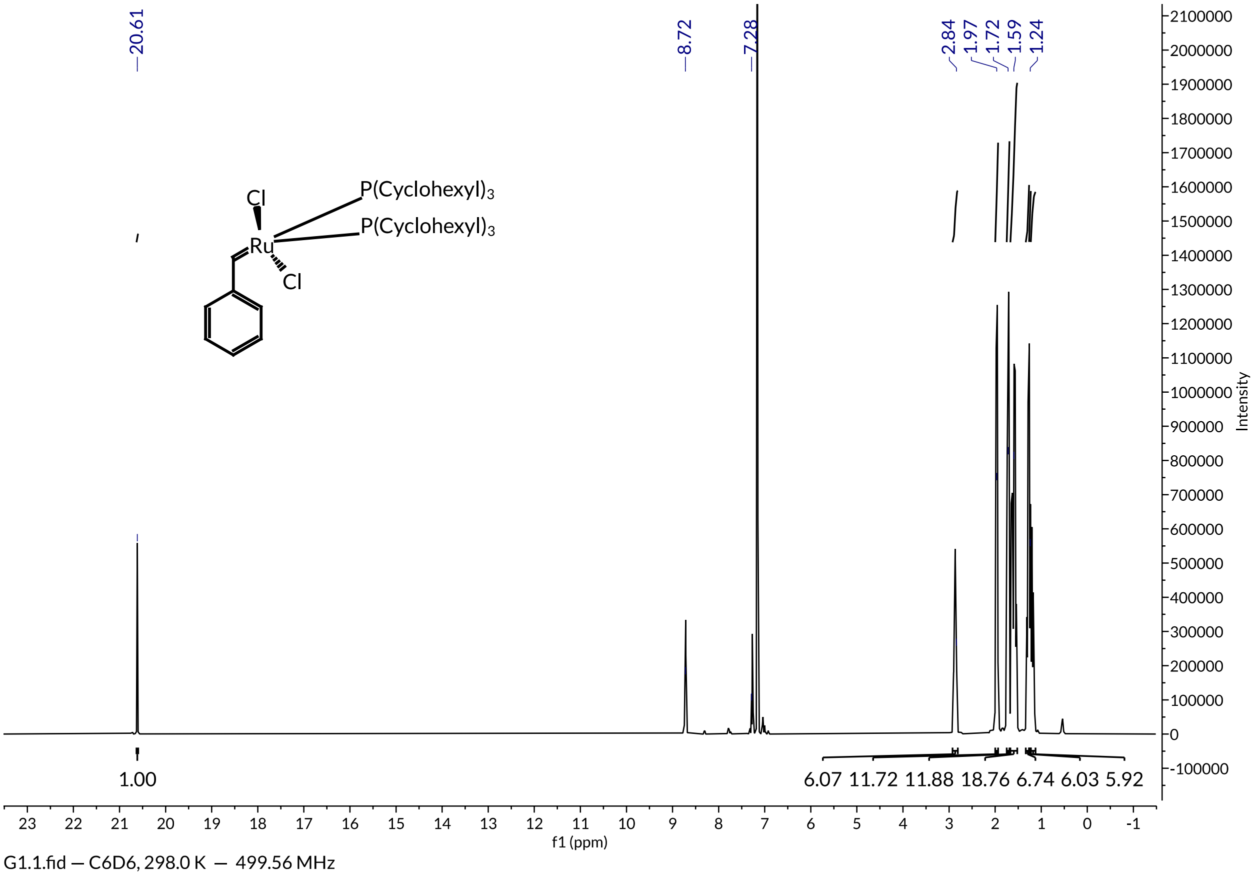

Figure S8: ^{1}H NMR (C_6D_6, 500 MHz) **of Grubbs' 1st Generation (G1)**

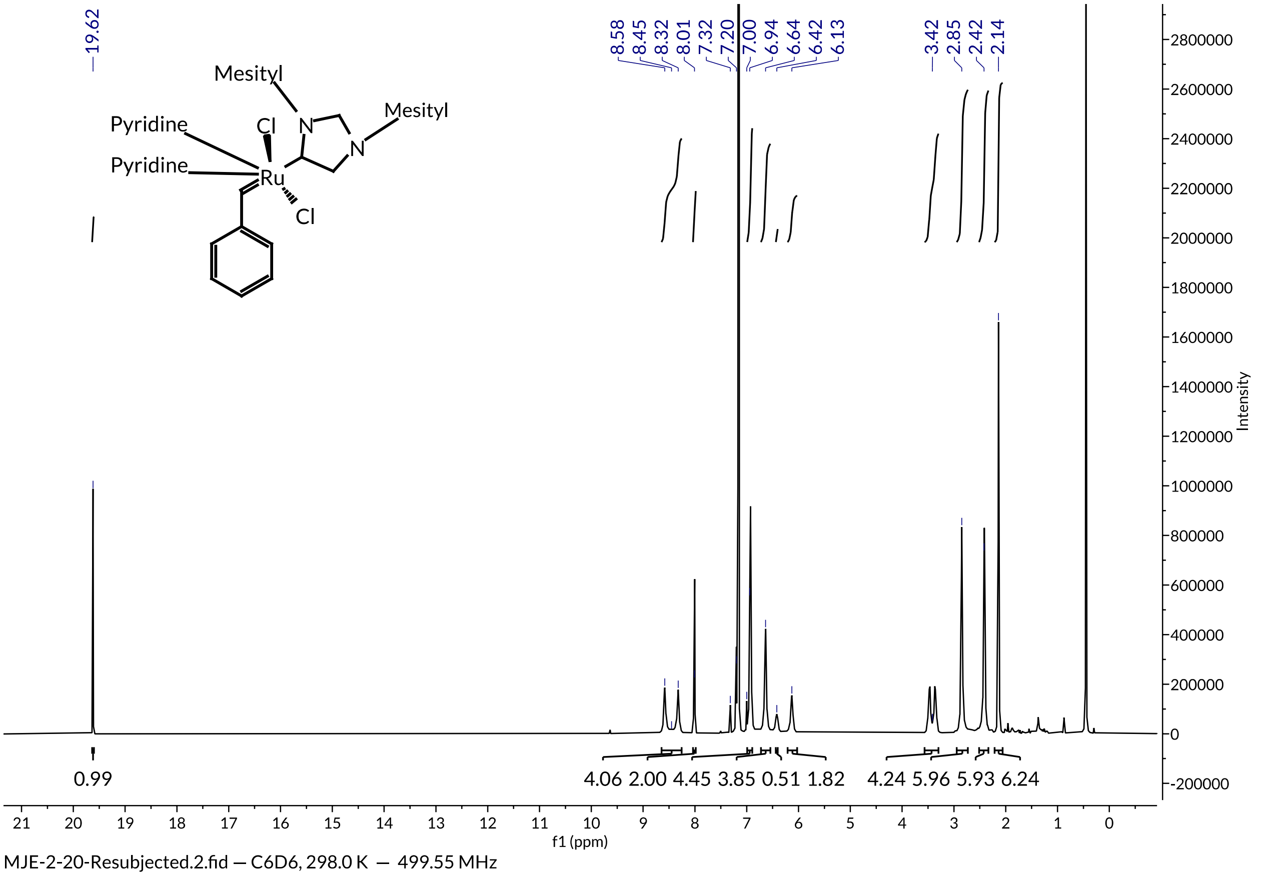

Figure S9: ^{1}H NMR (C_6D_6, 500 MHz) **of Grubbs' 3rd Generation (G3)**

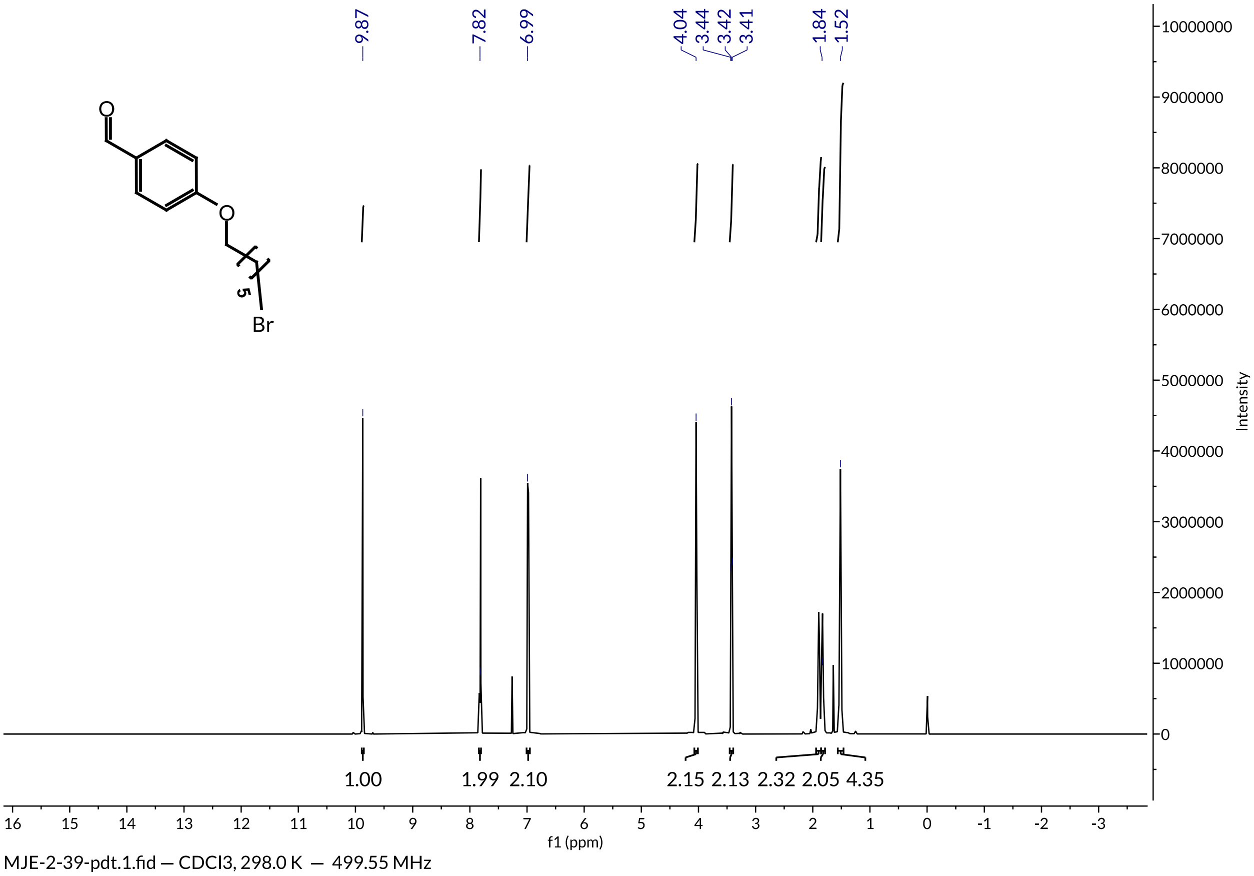

Figure S10: ^{1}H NMR ($CDCl_3$, 500 MHz) of 4-((6-bromohexyl)oxy)benzaldehyde (**S1**).

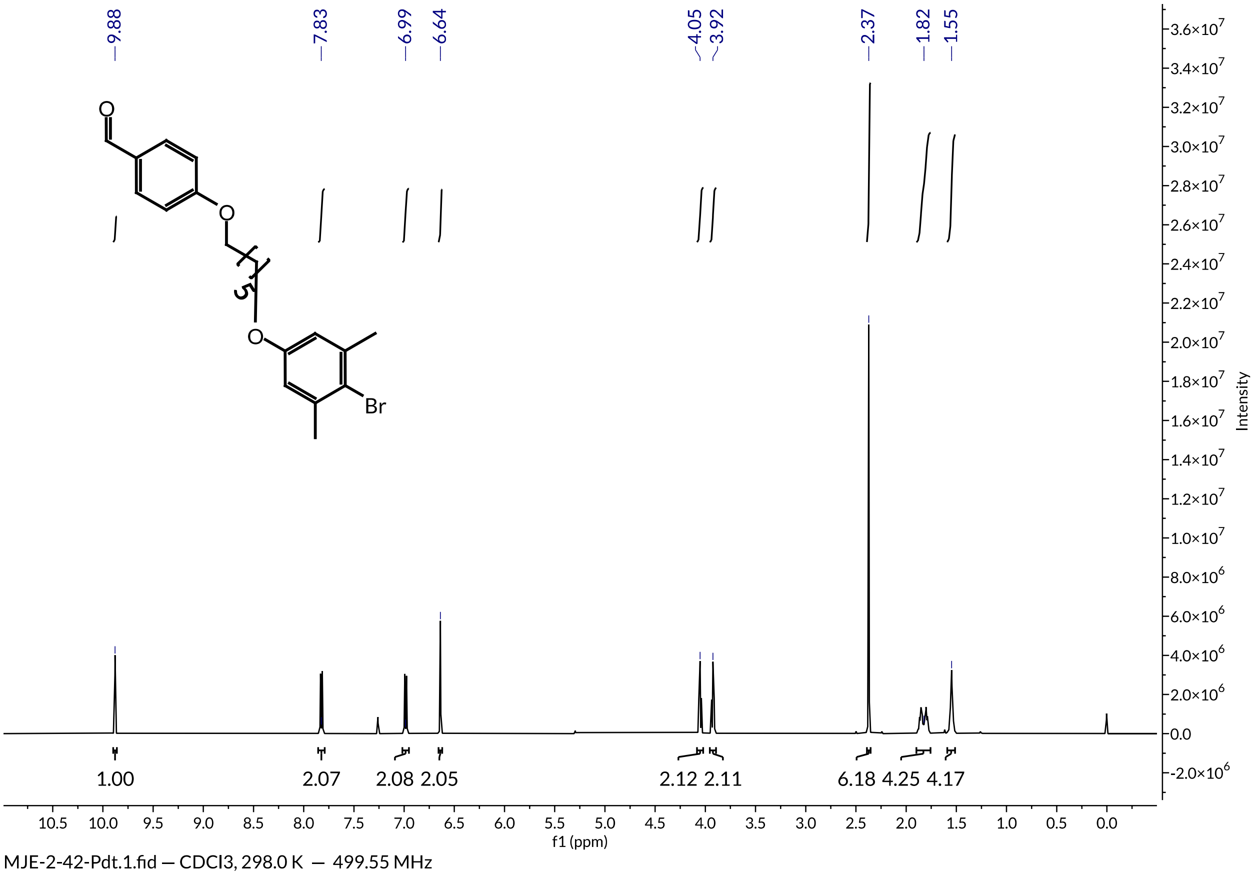

Figure S11: ^{1}H NMR ($CDCl_3$, 500 MHz) of 4-((6-(4-bromo-3,5-dimethylphenoxy)hexyl)oxy)benzaldehyde (**S2**).

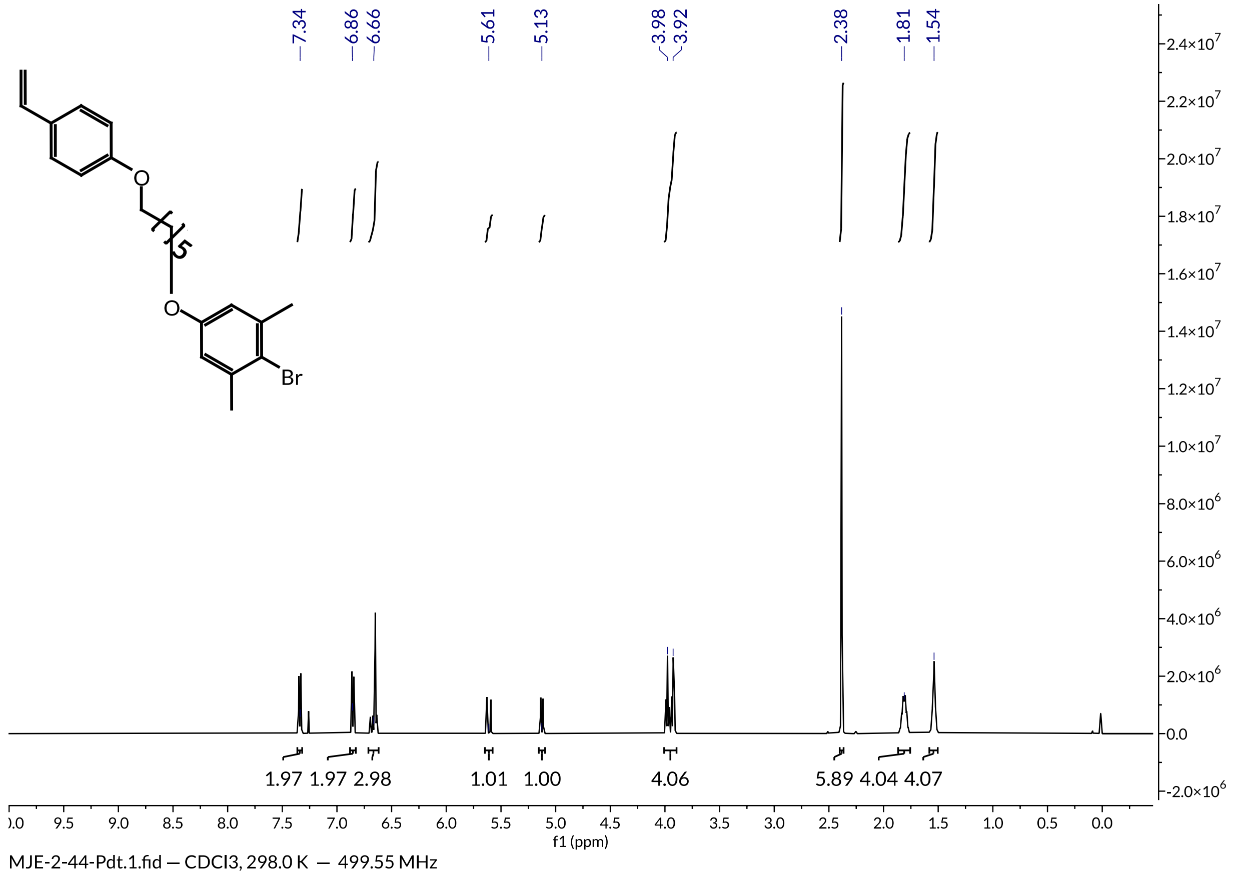

Figure S12: ^{1}H NMR ($CDCl_3$, 500 MHz) of 2-bromo-1,3-dimethyl-5-((6-(4-vinylphenoxy)hexyl)oxy)benzene (**S3**).

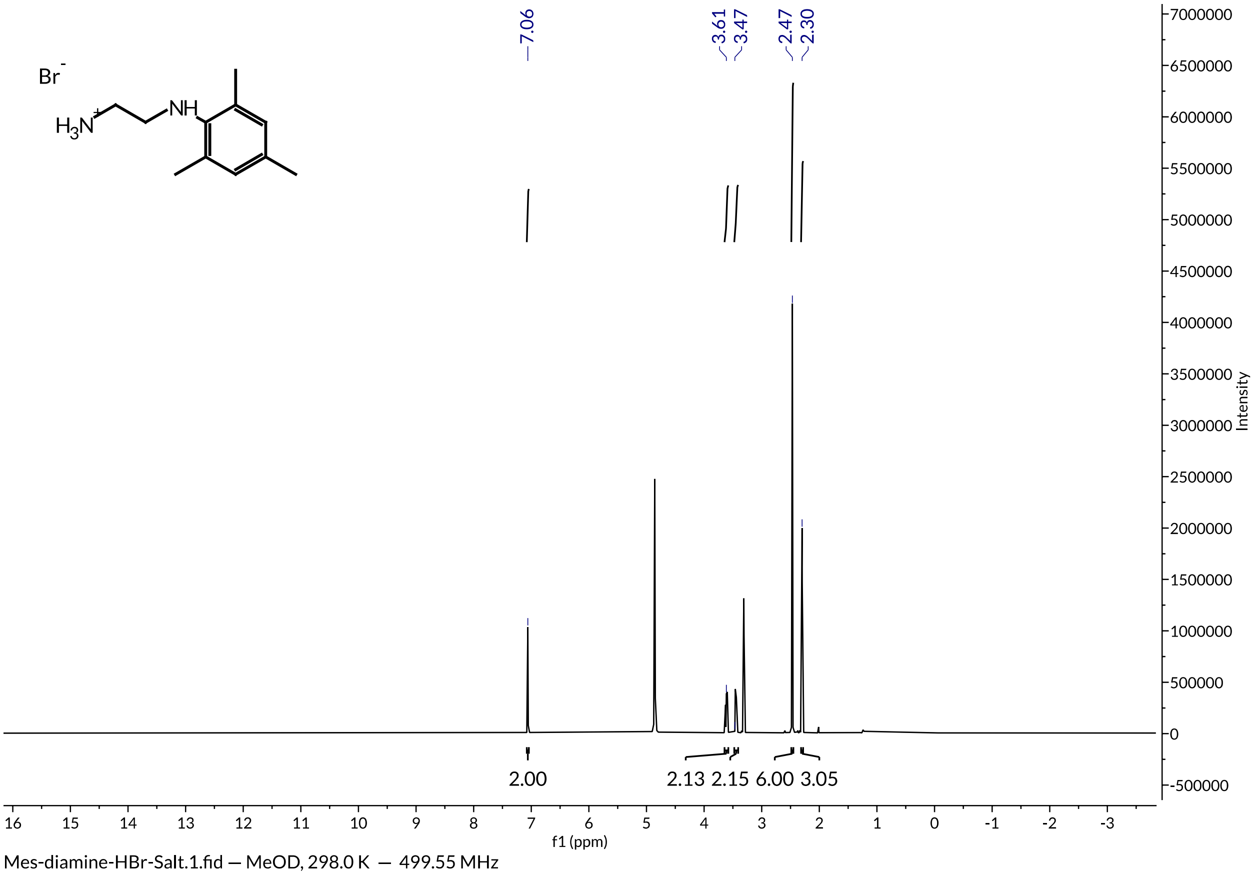

Figure S13: ^{1}H NMR (CD_3OD, 500 MHz) of *N*-mesitylethane-1,2-diamine•HBr salt **(Mes-diamine•HBr)**

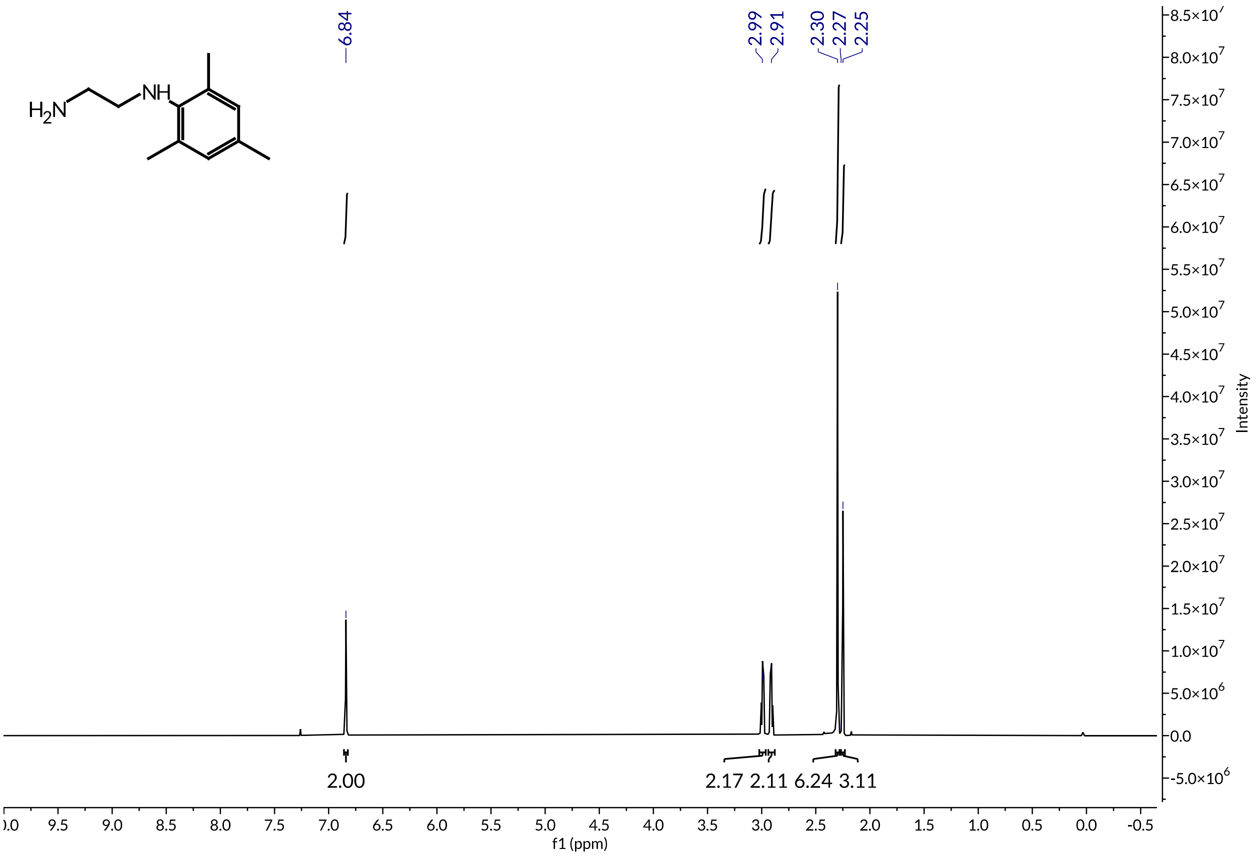

Figure S14: ^{1}H NMR ($CDCl_3$, 500 MHz) of *N*-mesitylethane-1,2-diamine (**Mes-diamine**)

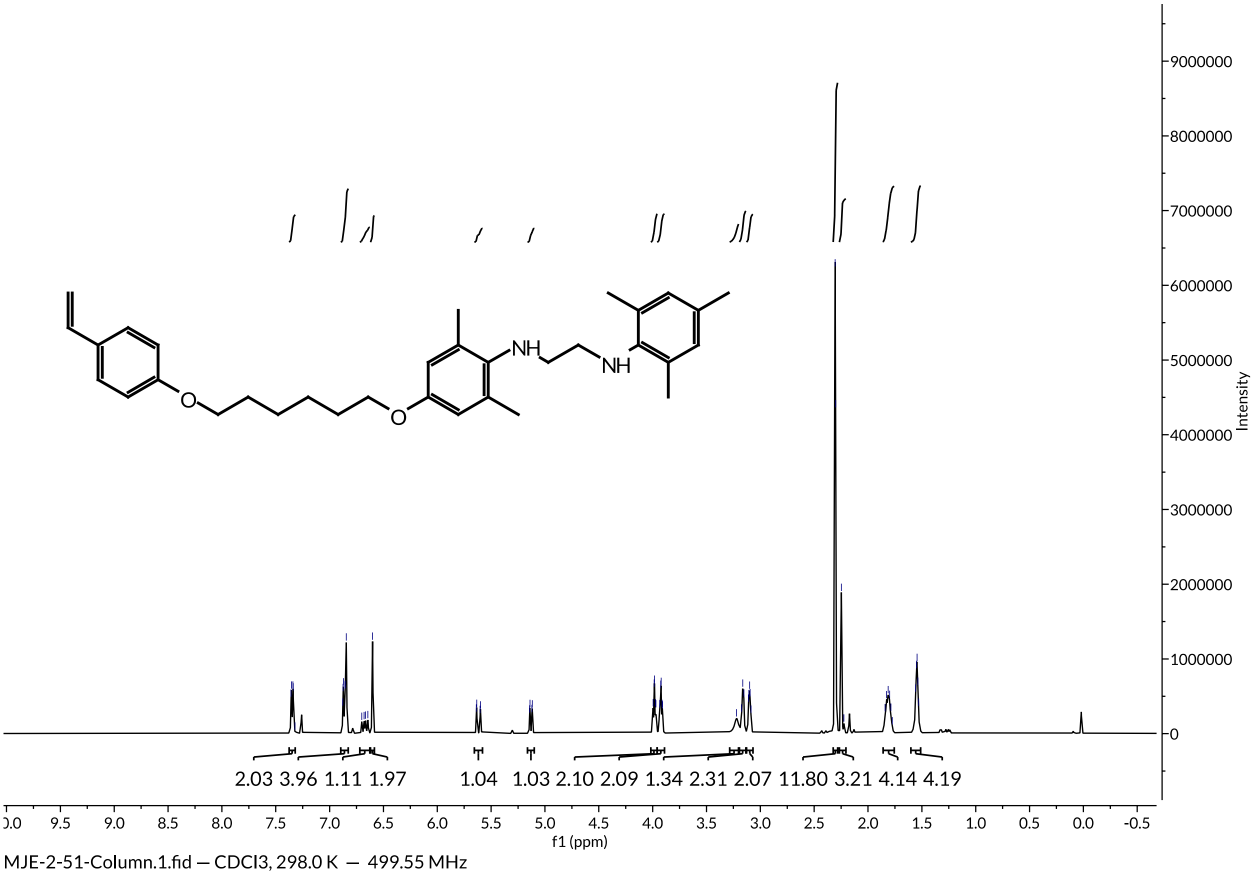

Figure S15: ^{1}H NMR ($CDCl_3$, 500 MHz) of *N*-(2,6-dimethyl-4-(4-(4-vinylphenoxy)butoxy)phenyl)-*N*-(3,4,5-trimethylphenyl)ethane-1,2-diamine (**S4**)

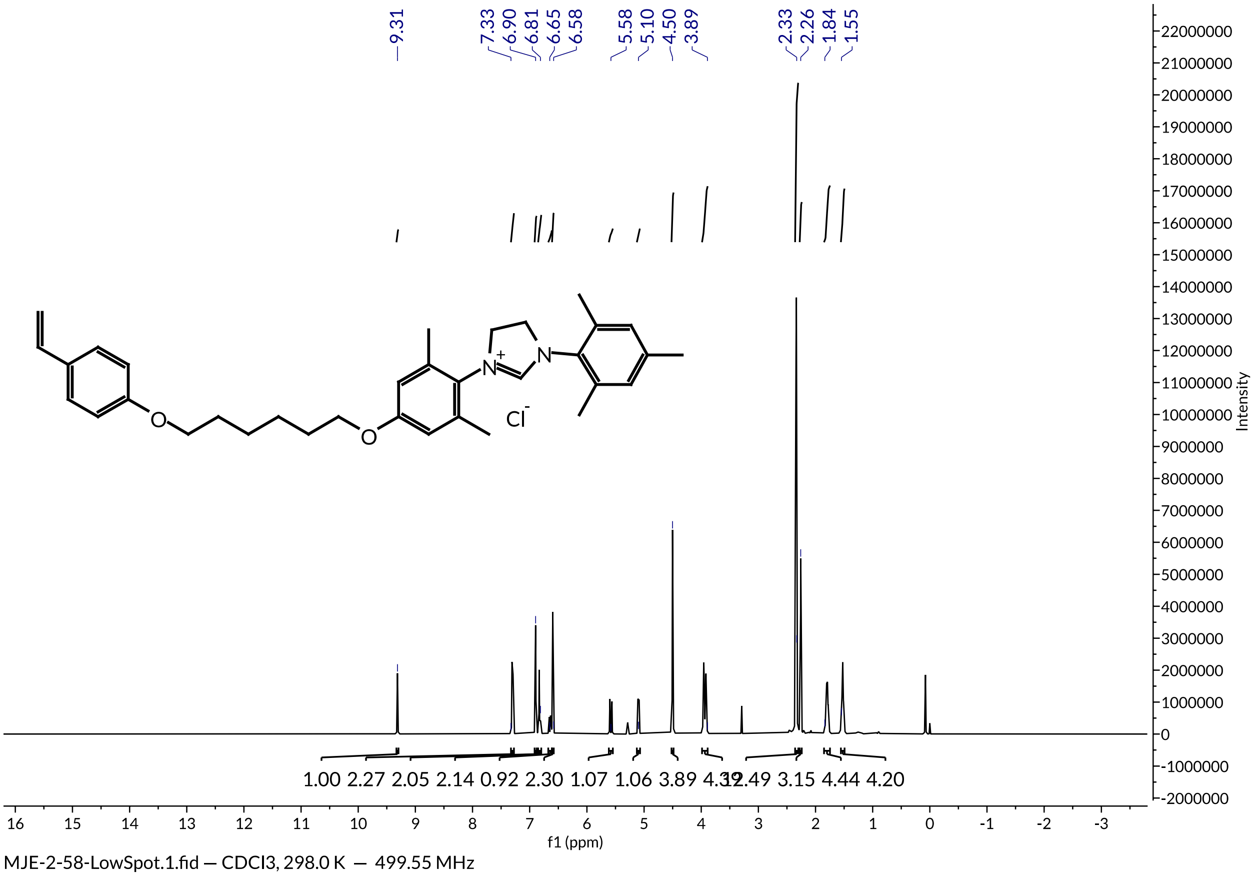

Figure S16: ^{1}H NMR ($CDCl_3$, 500 MHz) of 3-(2,6-dimethyl-4-(4-(4-vinylphenoxy)butoxy)phenyl)-1-mesityl-4,5-dihydro-imidazol-3-ium salt (**S5**).

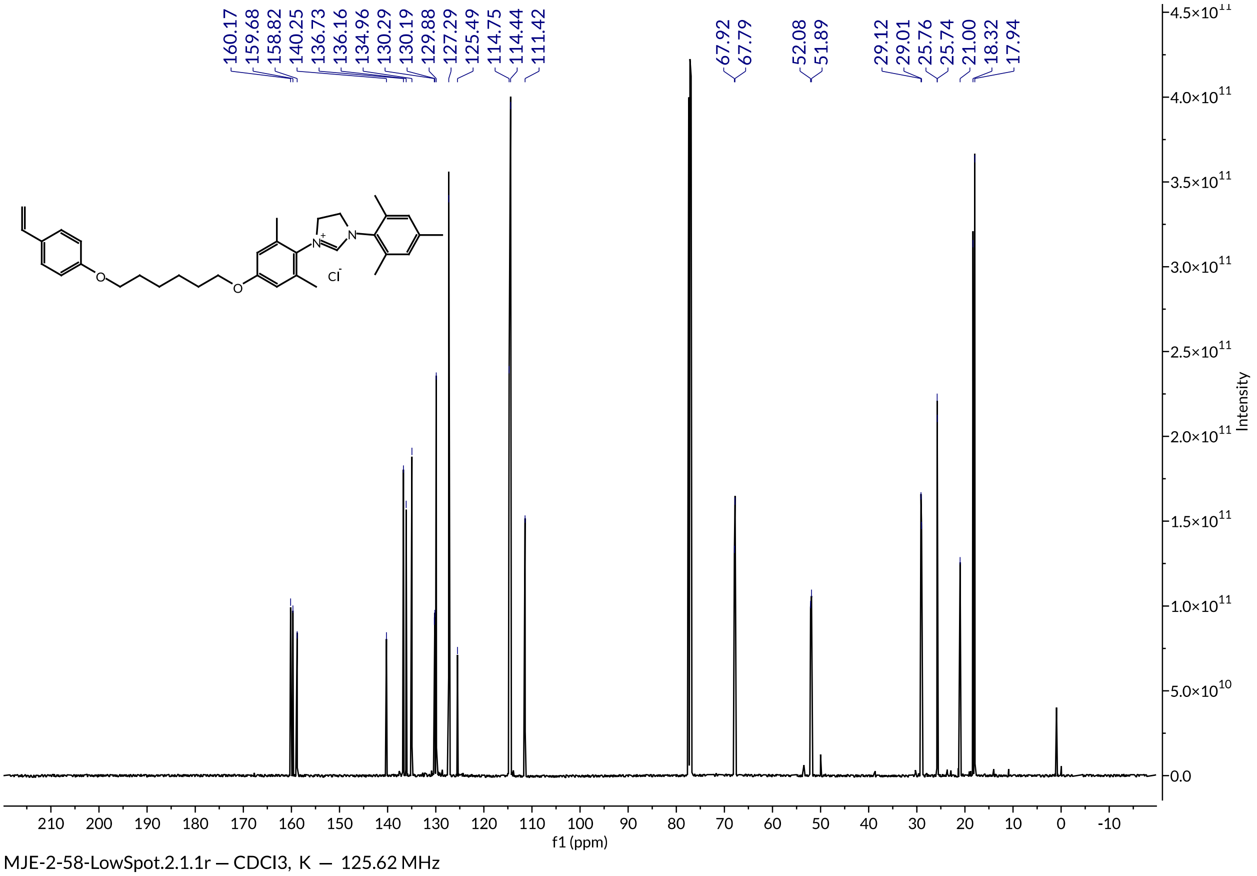

Figure S17: ^{13}C NMR ($CDCl_3$, 126 MHz) of 3-(2,6-dimethyl-4-(4-(4-vinylphenoxy)butoxy)phenyl)-1-mesityl-4,5-dihydro-imidazol-3-ium salt (**S5**).

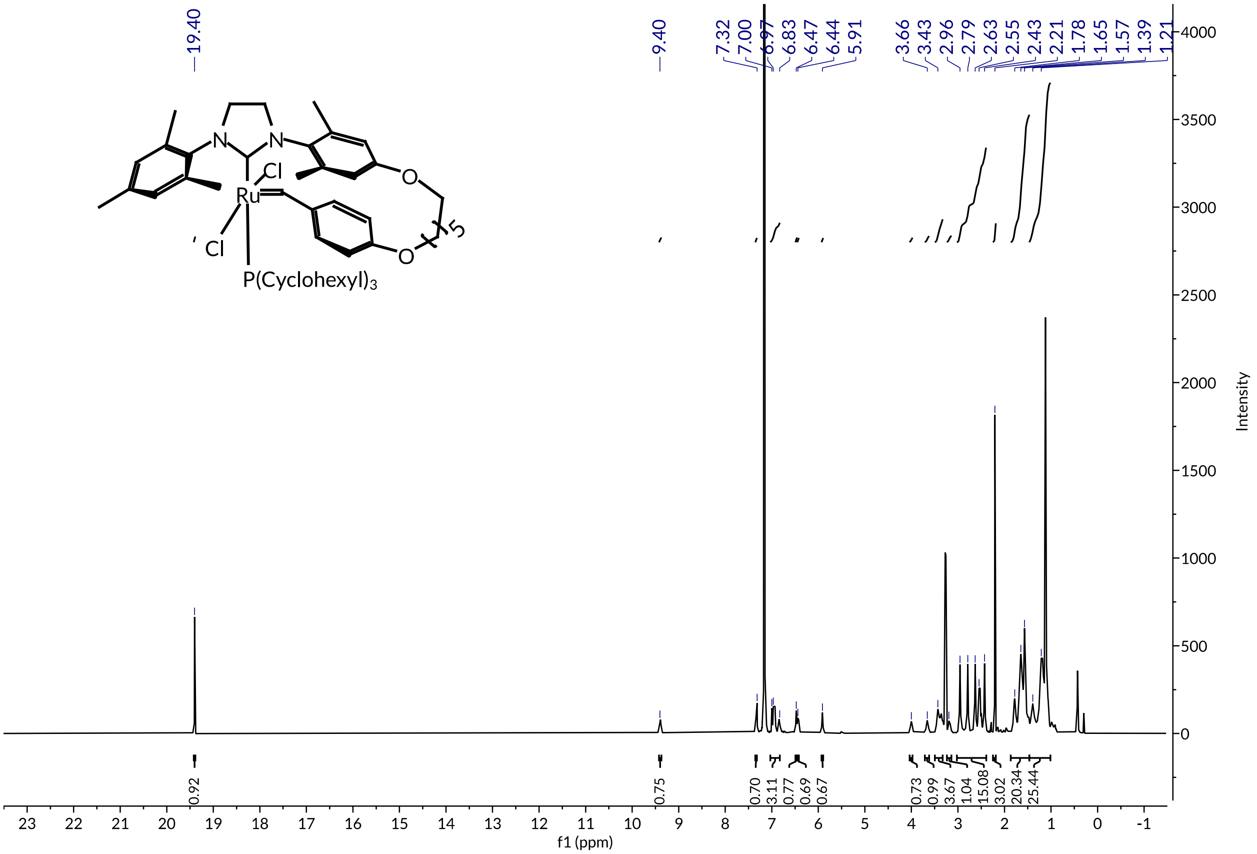

AL-1-161-gpcC 2.3.fid — C6D6, 298.1 K — 499.66 MHz

Figure S18: ^{1}H NMR (C_6D_6, 500 MHz) of **CB6** (purified va prep-GPC).

4: GPC-MALS-RI DATA:

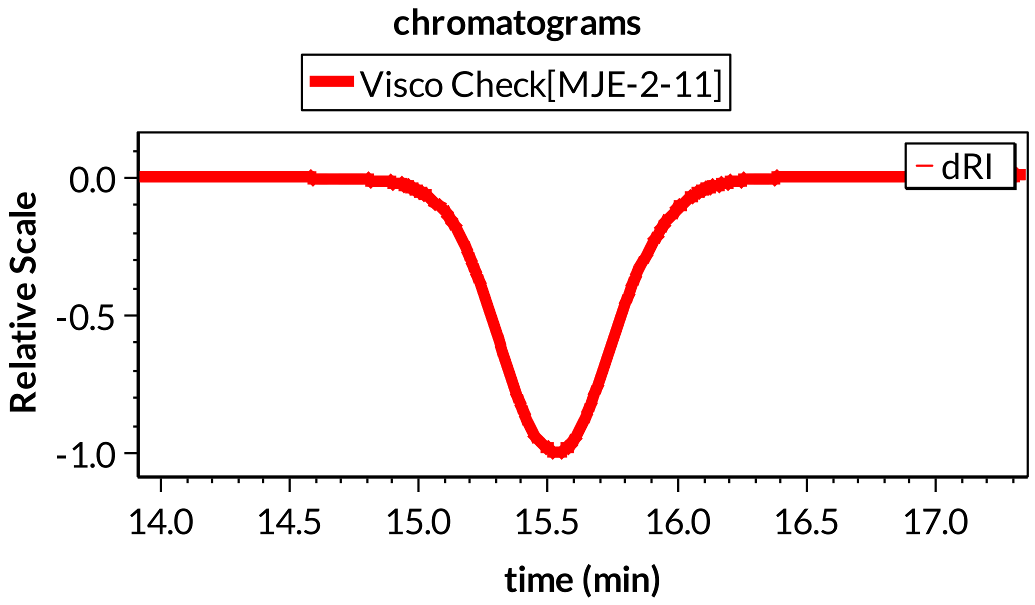

Figure S19: dRI trace of **PDMS-MM** with M_n = 7.0 kDa, M_w = 7.3 kDa, $Đ$ = 1.05, and dn/dc = –0.0232 (estimated via 100% mass recovery)

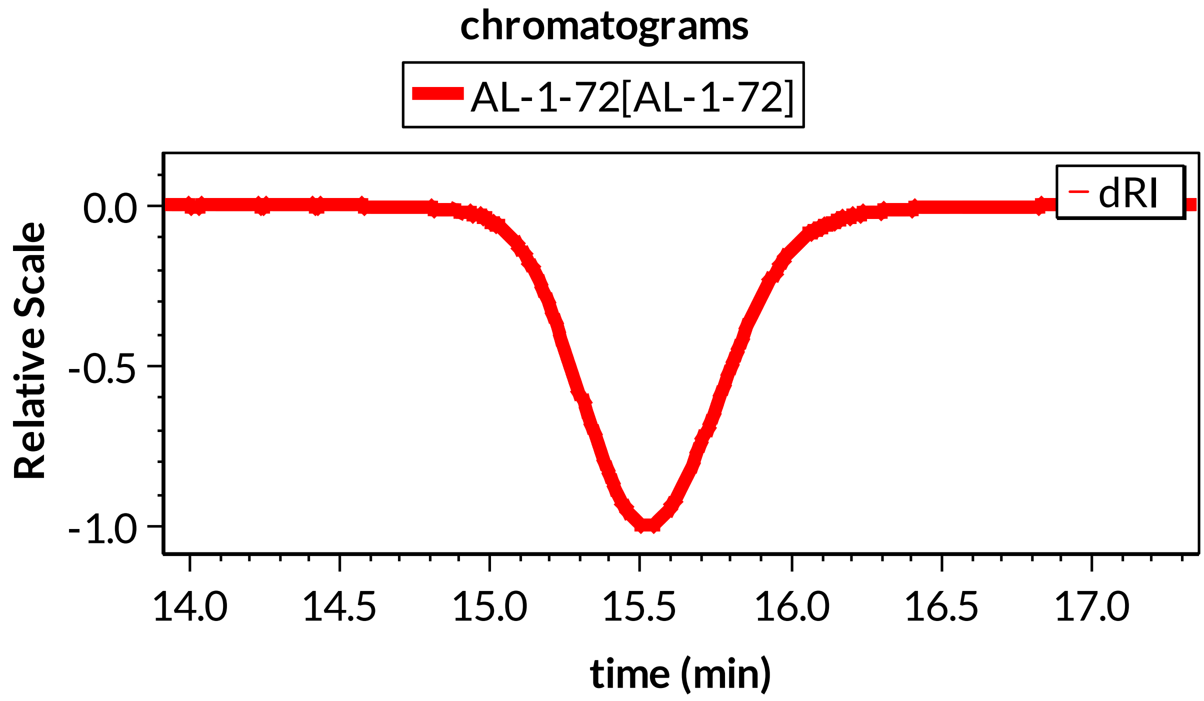

Figure S20: dRI trace of **PDMS-MM** with M_n = 6.3 kDa, M_w = 6.4 kDa, $Đ$ = 1.03, and dn/dc = –0.0258 (estimated via 100% mass recovery)

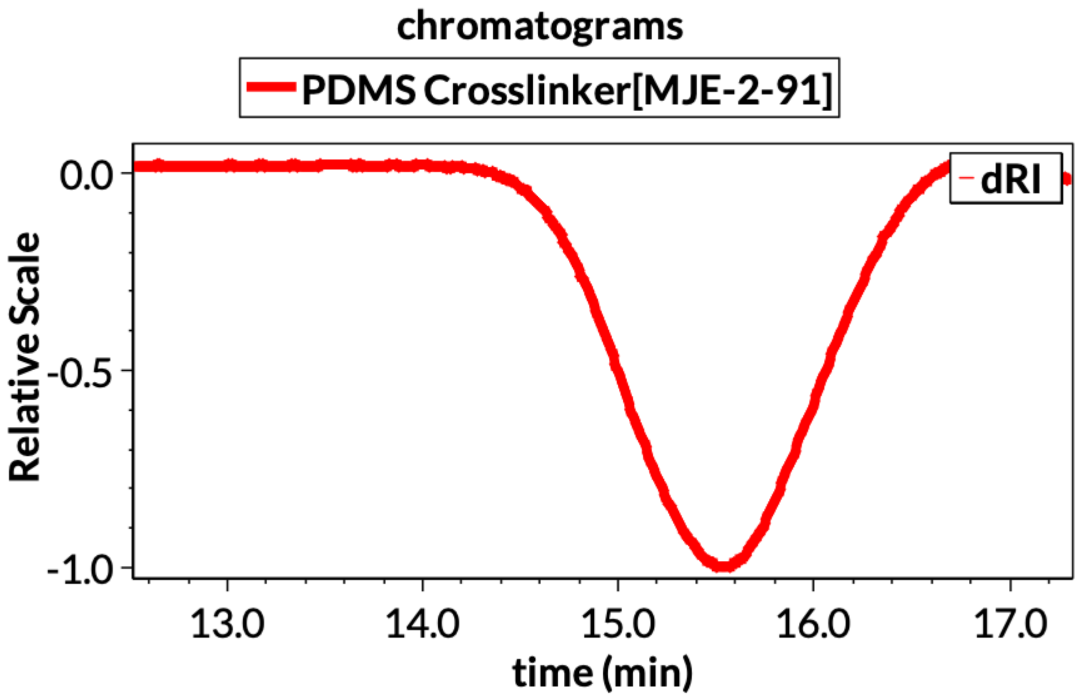

Figure S21: dRI trace of **BisBP-PDMS** with M_n = 22.3 kDa, M_w = 28.4 kDa, $Đ$ = 1.3, and dn/dc = –0.0135 (estimated via 100% mass recovery). Due to unreliable dn/dc estimations for this sample, the NMR M_n of 5.8 kDa was used for stoichiometric calculations (see **Figure S4** for NMR data).

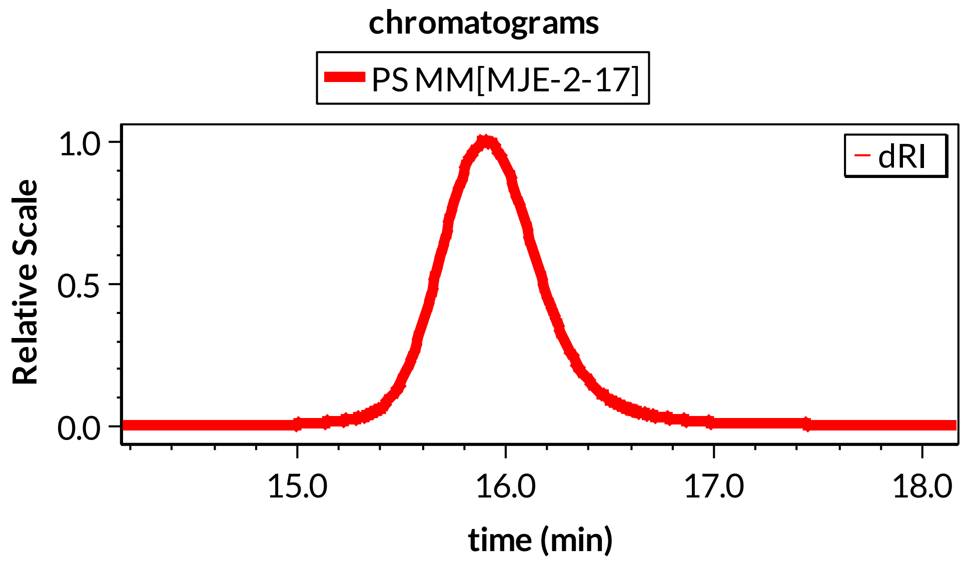

Figure S22: dRI trace of **PS-MM** with M_n = 4.6 kDa, M_w = 4.7 kDa, $Đ$ = 1.02, and dn/dc = 0.1361 (estimated via 100% mass recovery)

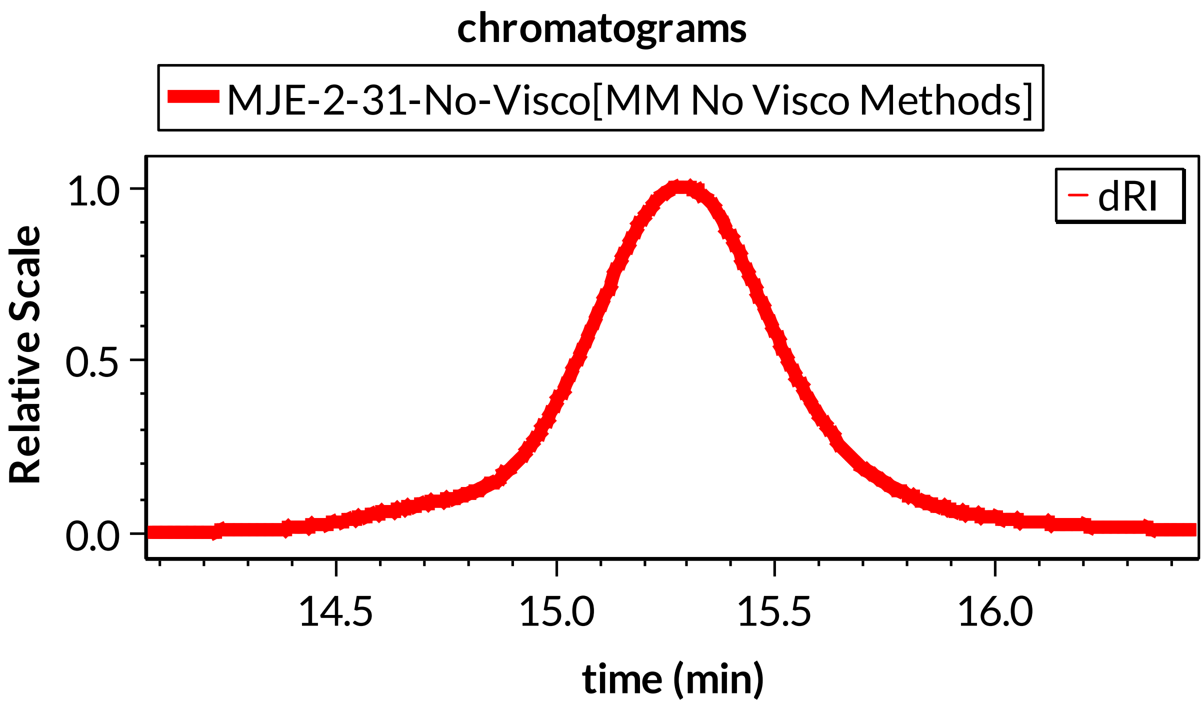

Figure S23: dRI trace of **PS-MM** with M_n = 8.3 kDa, M_w = 8.5 kDa, $Đ$ = 1.03, and dn/dc = 0.1517 (estimated via 100% mass recovery)

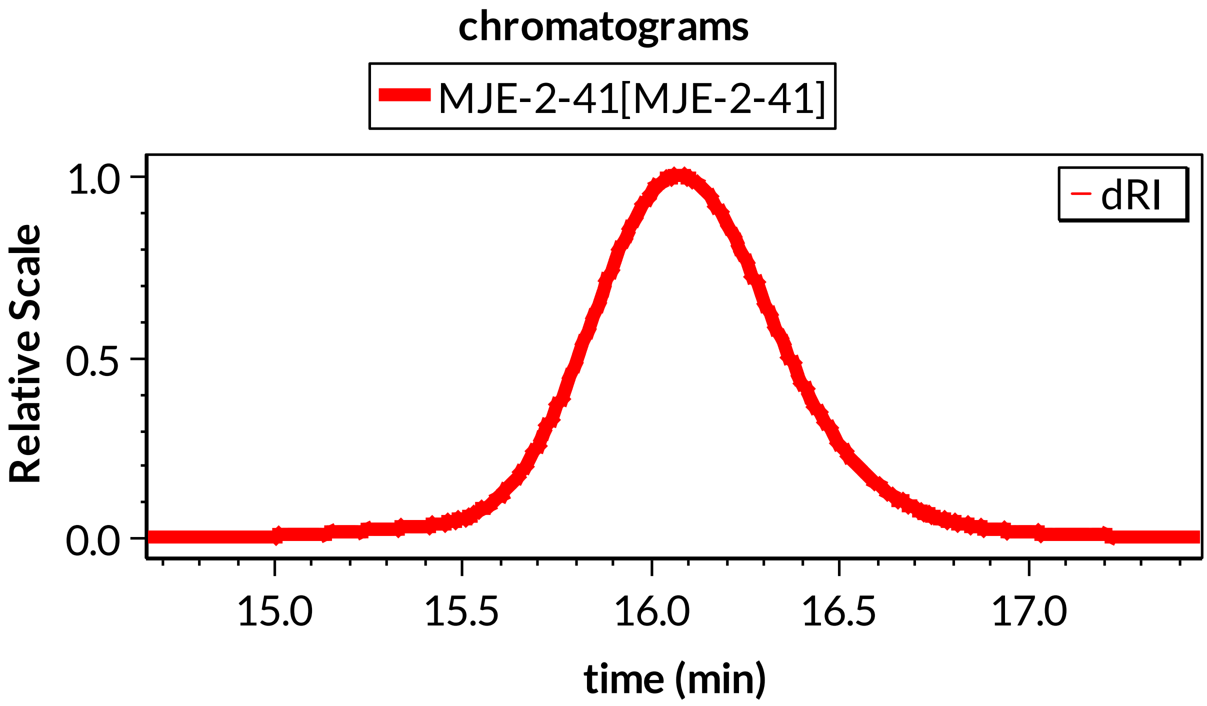

Figure S24: dRI trace of **PS-MM** with M_n = 4.4 kDa, M_w = 4.5 kDa, $Đ$ = 1.03, and dn/dc = 0.1314 (estimated via 100% mass recovery)

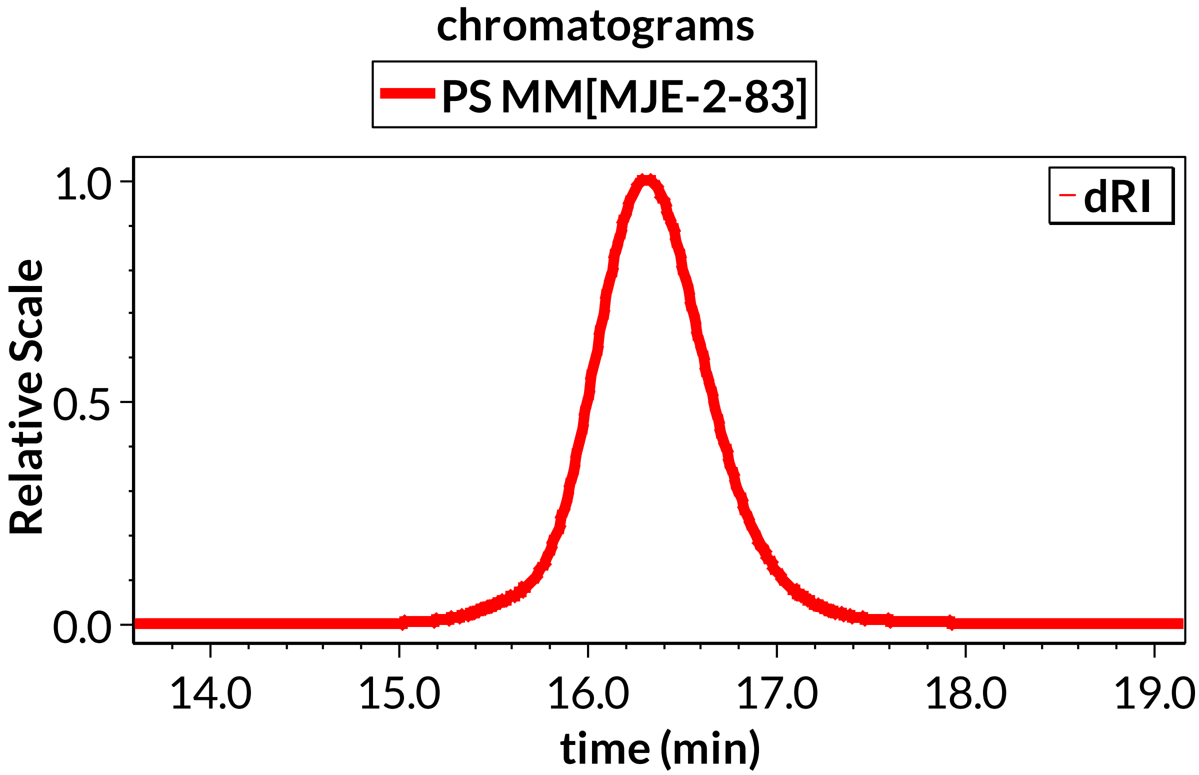

Figure S25: dRI trace of **PS-MM** with M_n = 4.7 kDa, M_w = 4.9 kDa, $Đ$ = 1.05, and dn/dc = 0.1649 (estimated via 100% mass recovery)

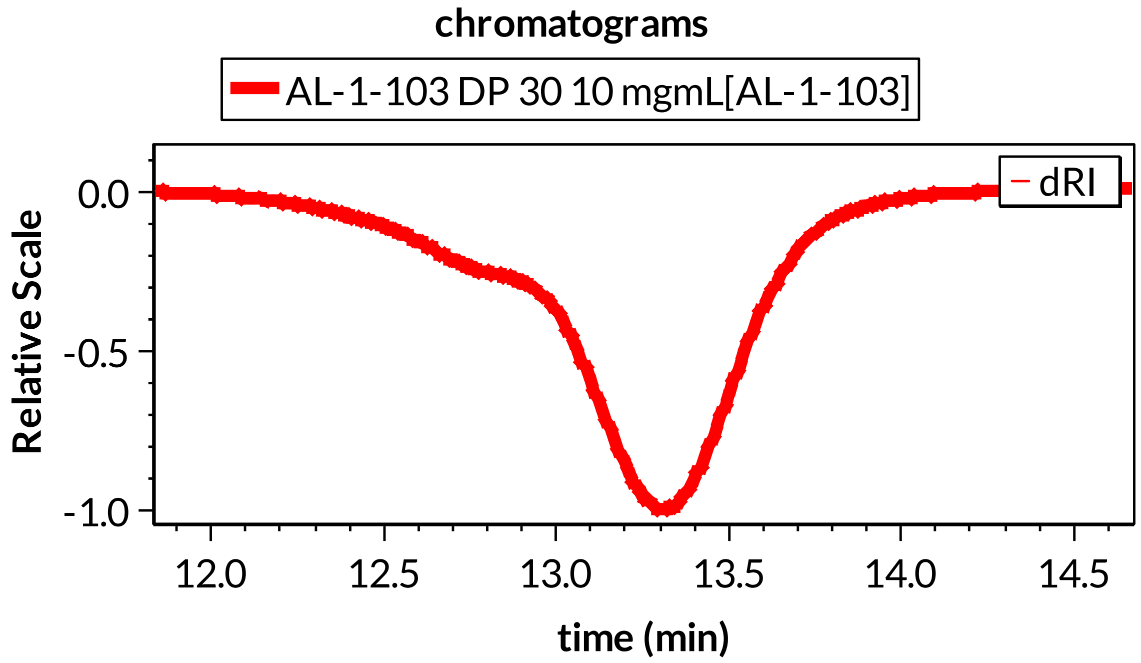

Figure S26: Representative dRI trace of linear **PDMS-BBP** with M_n = 350 kDa, M_w = 380 kDa, $Đ$ = 1.1, and dn/dc = –0.0177 (measured directly via batch injection). Target DP = 30 from **PDMS-MM** with M_n = 6.3 kDa.

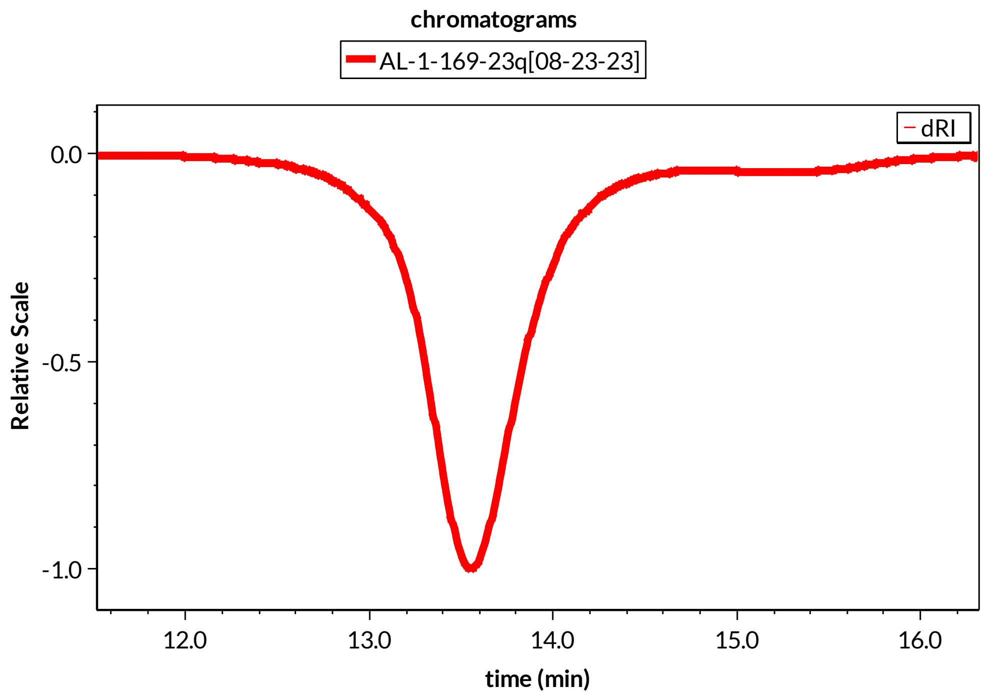

Figure S27: Representative dRI trace of cyclic **PDMS-BBP** with M_n = 260 kDa, M_w = 280 kDa, $Đ$ = 1.1, and dn/dc = –0.0115 (measured directly via batch injection). Target DP = 10 from **PDMS-MM** with M_n = 6.3 kDa.

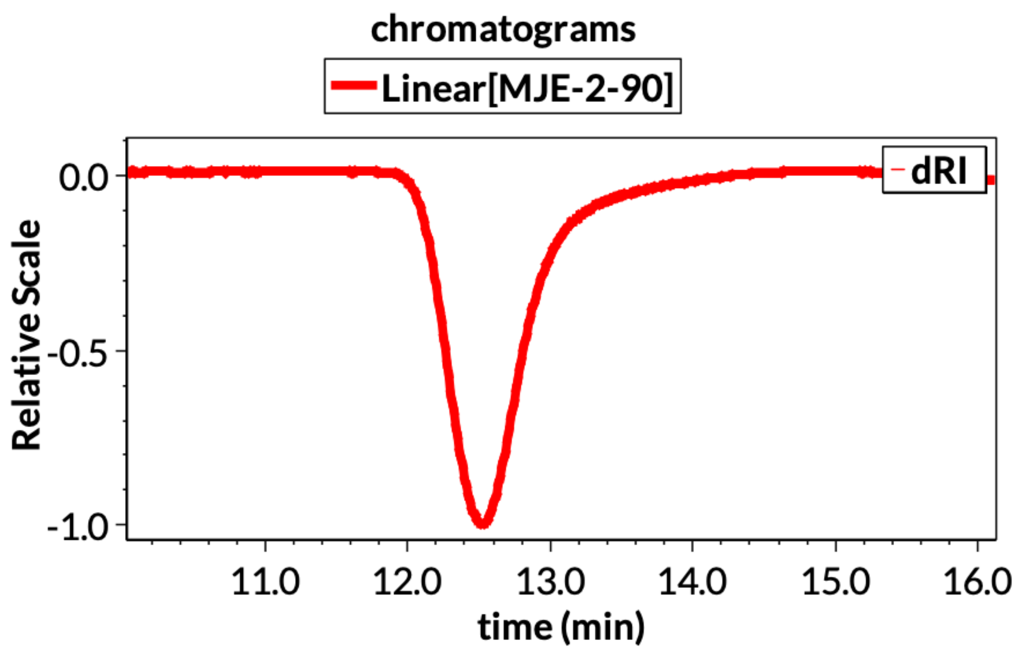

Figure S28: Representative dRI trace of linear **PDMS-BBP** with M_n = 1,730 kDa, M_w = 1,820 kDa, $Đ$ = 1.1, and dn/dc = –0.0177 (measured directly via batch injection). Target DP = 300 from **PDMS-MM** with M_n = 7.0 kDa. This sample was used to prepare cyclic polymer networks for compression testing.

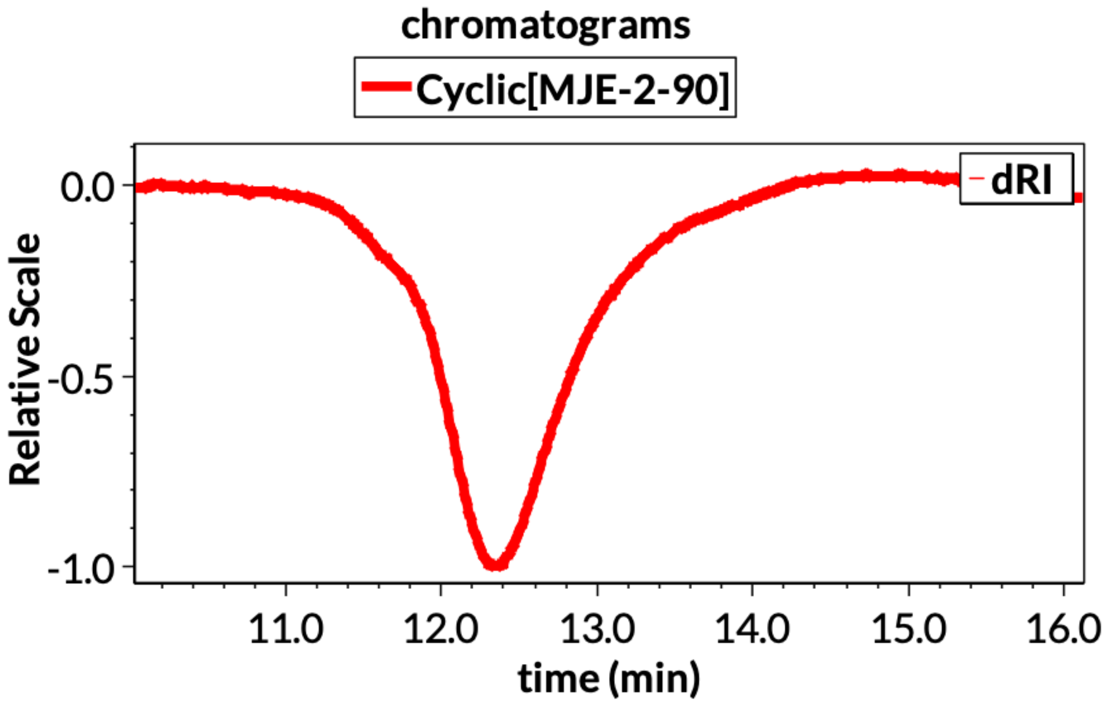

Figure S29: Representative dRI trace of cyclic **PDMS-BBP** with M_n = 3,490 kDa, M_w = 3,810 kDa, $Đ$ = 1.1, and *dn*/*dc* = –0.0115 (measured directly via batch injection). Target DP = 100 from **PDMS-MM** with M_n = 7.0 kDa. This sample was used to prepare cyclic polymer networks for compression testing.

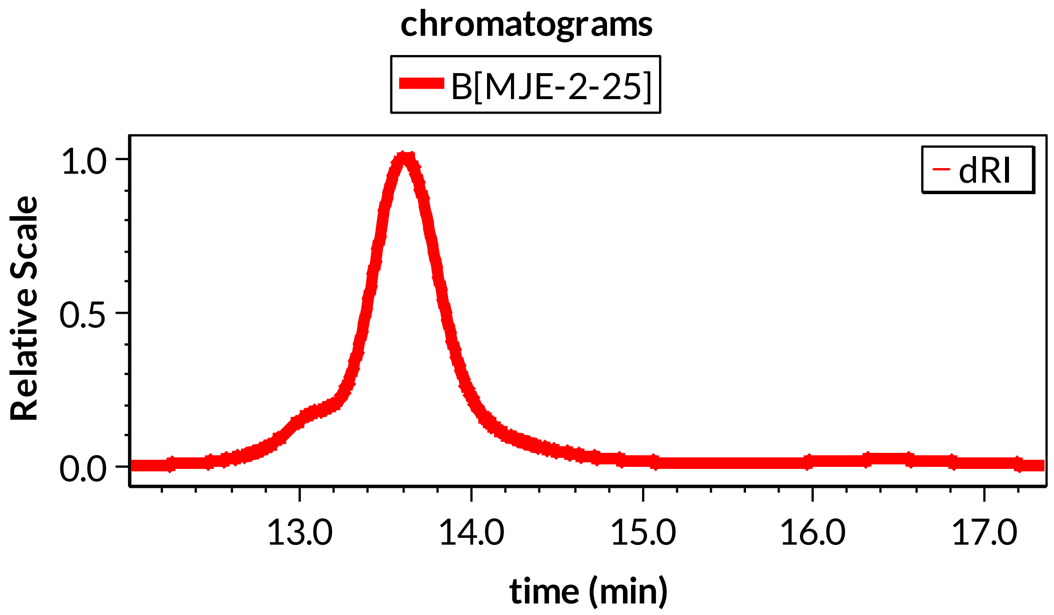

Figure S30: Representative dRI trace of linear **PS-BBP** with M_n = 100 kDa, M_w = 140 kDa, $Đ$ = 1.1, and *dn*/*dc* = 0.1706 (measured directly via batch injection). Target DP = 50 from **PS-MM** with M_n = 4.6 kDa.

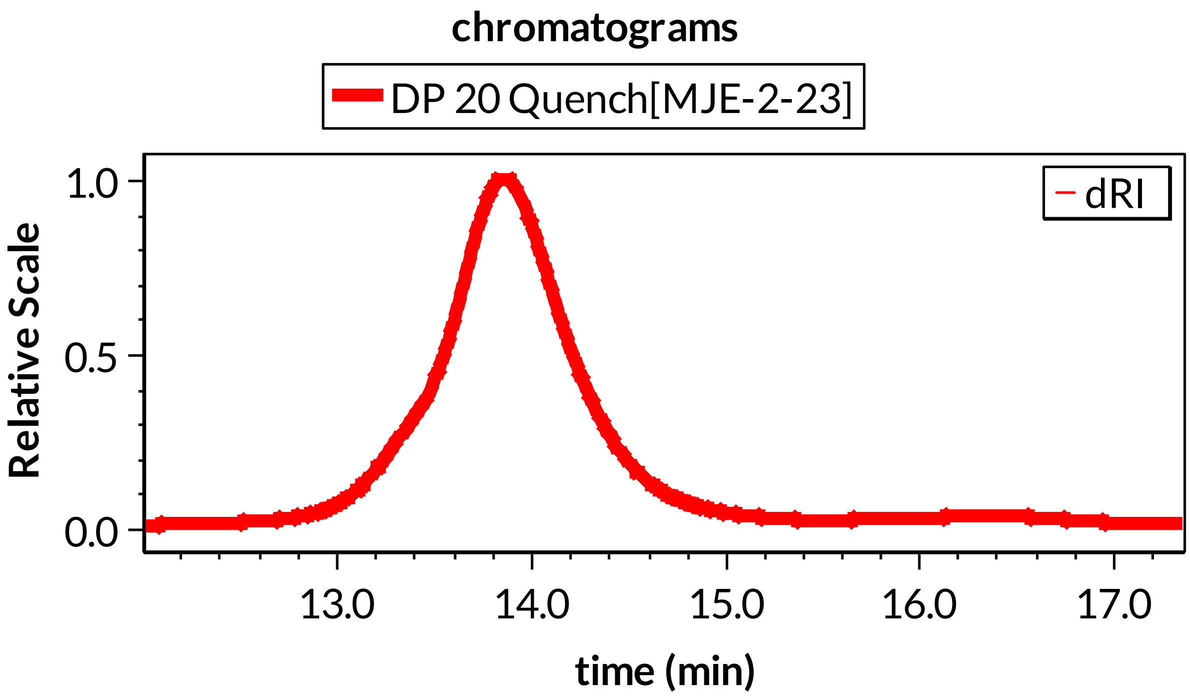

Figure S31: Representative dRI trace of cyclic **PS-BBP** with M_n = 130 kDa, M_w = 140 kDa, $Đ$ = 1.1, and dn/dc = 0.1119 (measured directly via batch injection). Target DP = 20 from **PS-MM** with M_n = 4.6 kDa.

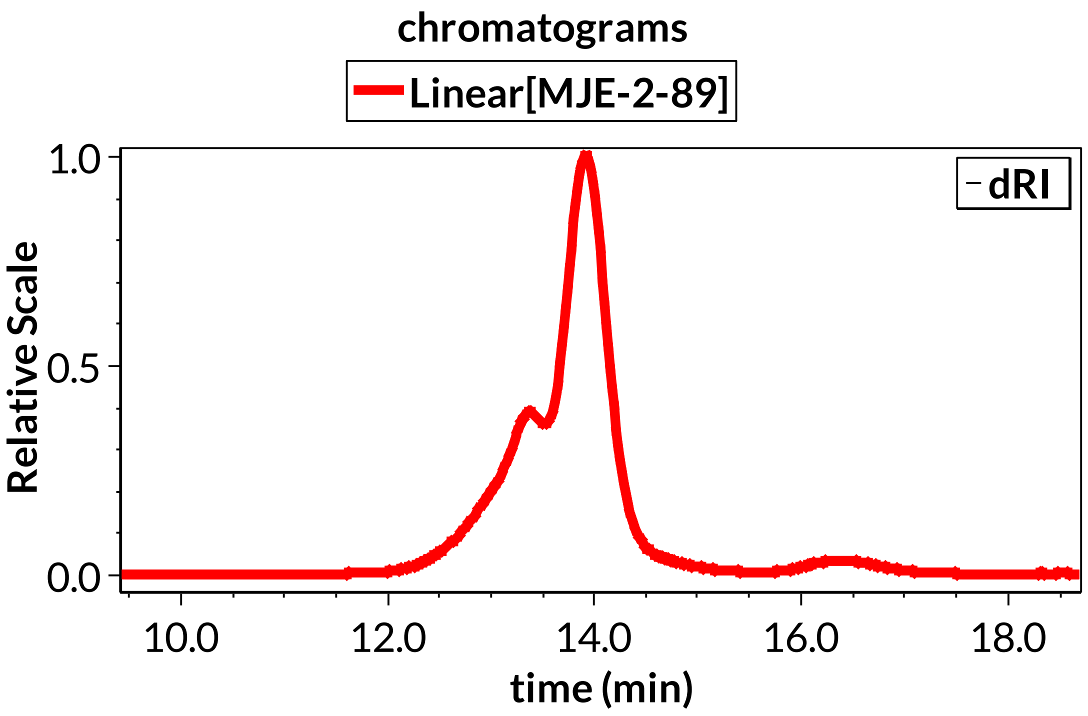

Figure S32: dRI trace of linear **PS-BBP** with M_n = 200 kDa, M_w = 260 kDa, $Đ$ = 1.3, and dn/dc = 0.1706 (measured directly via batch injection). Target DP = 71 from **PS-MM** with M_n = 4.7 kDa. ***Note: This polymer was used for thermal testing and ball-mill grinding kinetics experiments!***

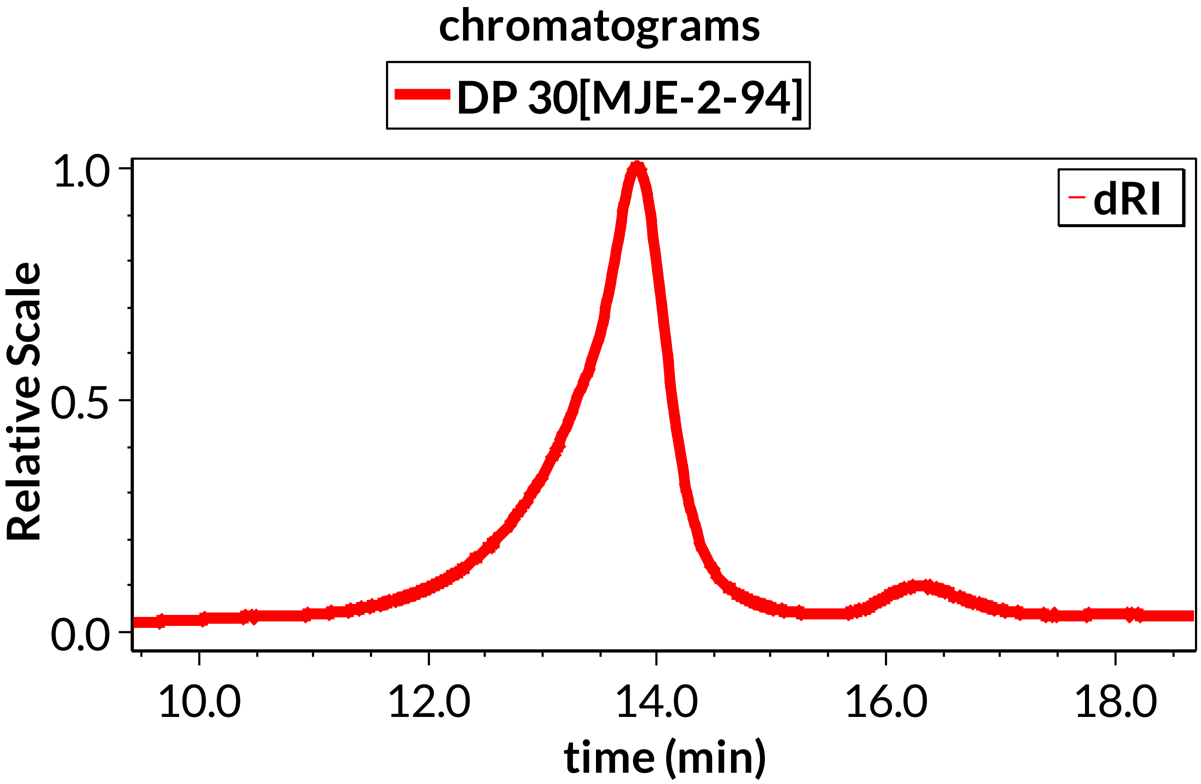

Figure S33: dRI trace of cyclic **PS-BBP** with M_n = 250 kDa, M_w = 330 kDa, $Đ$ = 1.3, and dn/dc = 0.1119 (measured directly via batch injection). Target DP = 30 from **PS-MM** with M_n = 4.7 kDa. ***Note: This polymer was used for thermal testing and ball-mill grinding kinetics experiments!***

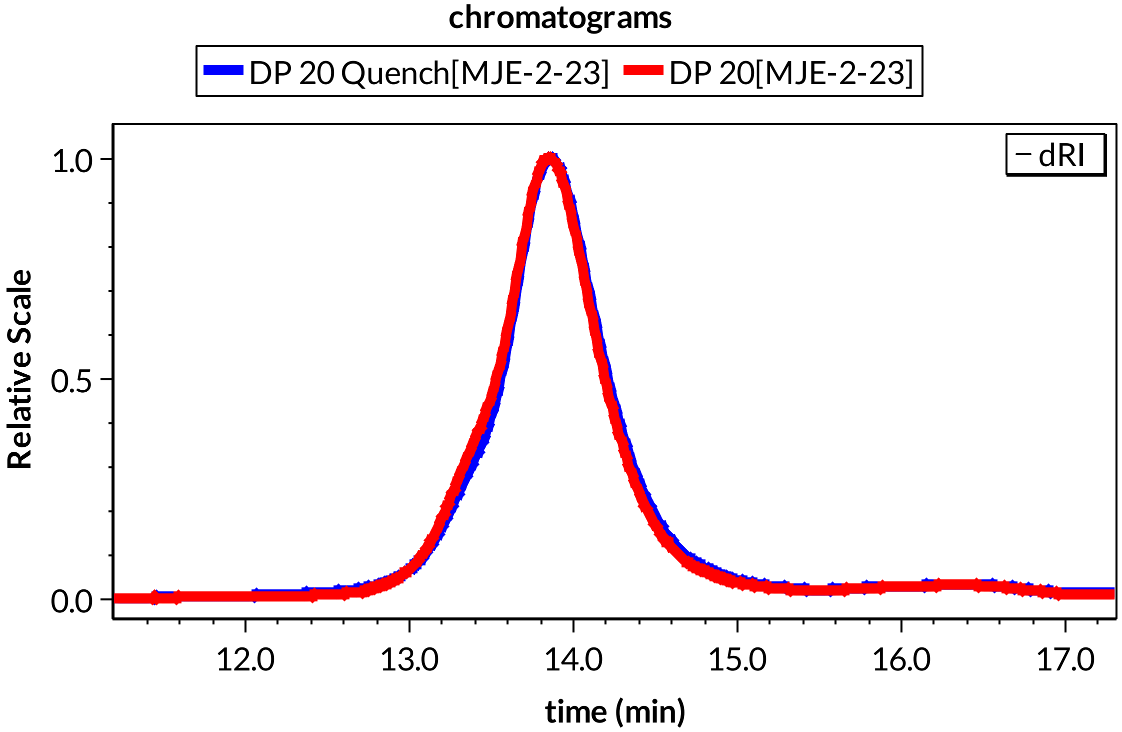

Figure S34: Representative dRI traces of quenched/unquenched cyclic **PS-BBP** samples. Target DP = 20 from **PS-MM** with M_n = 4.6 kDa.

Table S1: GPC-MALS data for quenched/unquenched cyclic PS samples.

**Sample**	**M_n (kDa)[a]:**	**M_w (kDa)[a]:**	**$Đ$[a]:**	**Retention Time (min):**
Quenched	130	140	1.1	13.84
Unquenched	120	140	1.2	13.85

[a] Determined by GPC-MALS.

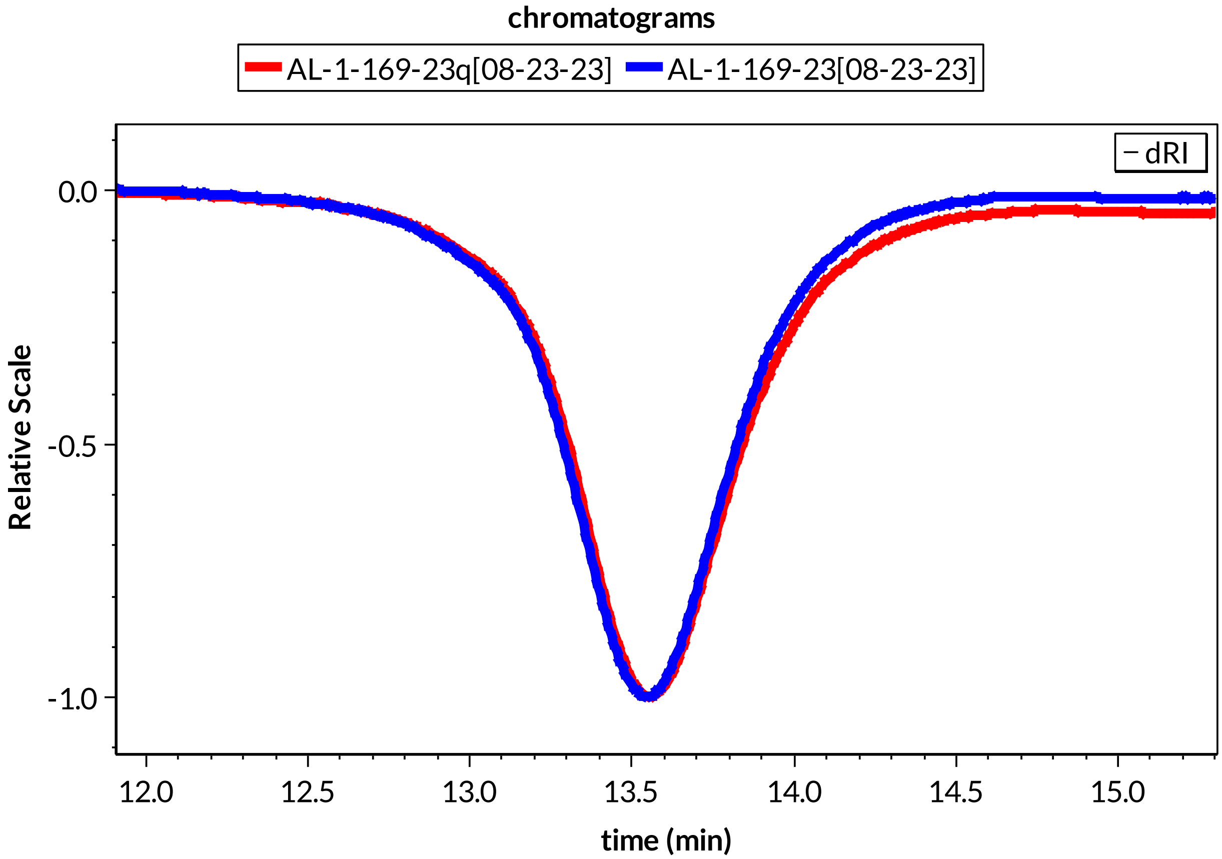

Figure S35: Representative dRI traces of quenched (red)/unquenched (blue) cyclic **PDMS-BBP** samples. Target DP = 10 from **PDMS-MM** with M_n = 6.3 kDa.

Table S2: GPC data for quenched/unquenched cyclic **PDMS-BBP** samples.

Sample	M_n (kDa)[a]:	M_w (kDa)[a]:	$Đ$[a]:	Retention Time (minutes):
Quenched	260	280	1.1	13.56
Unquenched	260	270	1.1	13.55

[a] Determined by GPC-MALS.

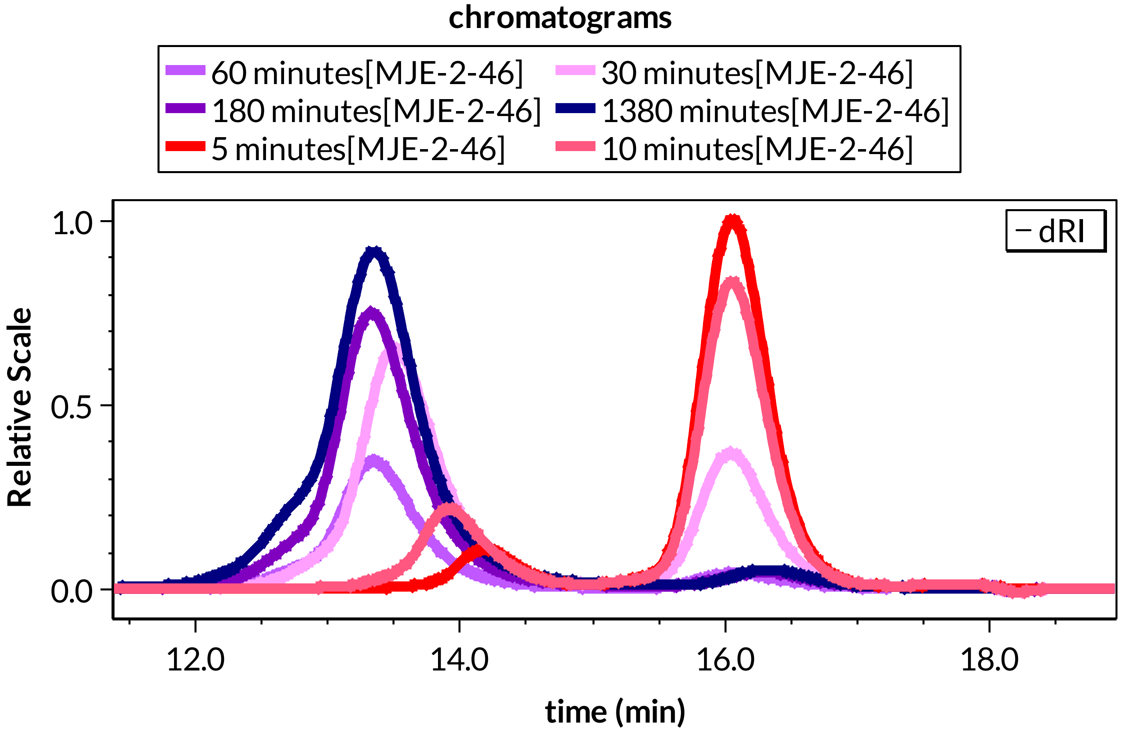

Figure S36: Time course study (5 min, 10 min, 30 min, 1 h, 3 h, 23 h) showing conversion of **PS-MM** with M_n = 4.4 kDa (peak at ca. 16 min) into cyclic BBP (reaction time = 23 h) with M_n = 320 kDa, M_w = 370 kDa, $Đ$ = 1.2, and dn/dc = 0.1119 (measured directly via batch injection). Target DP = 25.

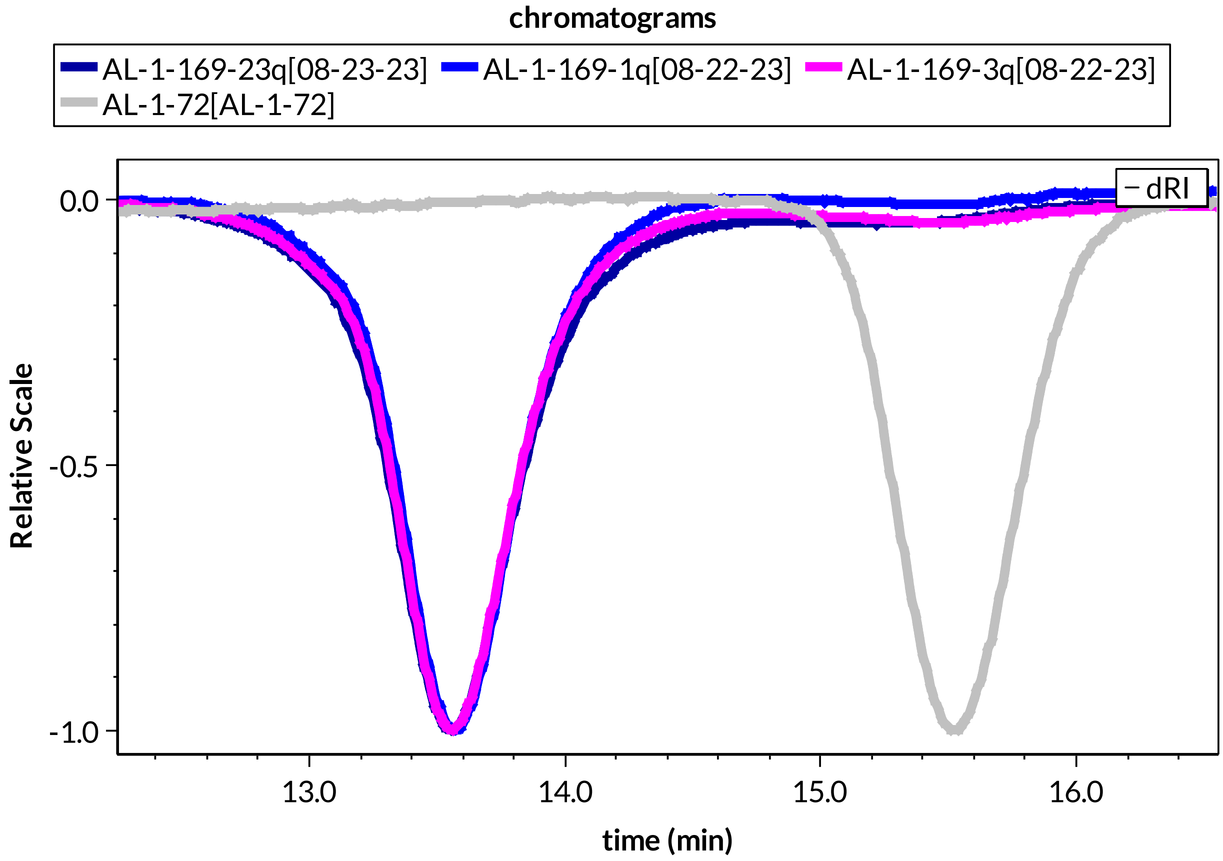

Figure S37: Time course study (1 h, 3 h, 23 h) showing conversion of **PDMS-MM** with M_n = 6.3 kDa (note: MM peak at 15.5 min plotted in gray as reference) into cyclic BBP (reaction time = 23 h) with M_n = 260 kDa, M_w = 280 kDa, $Đ$ = 1.1, and dn/dc = –0.0115 (measured directly via batch injection). Target DP = 10.

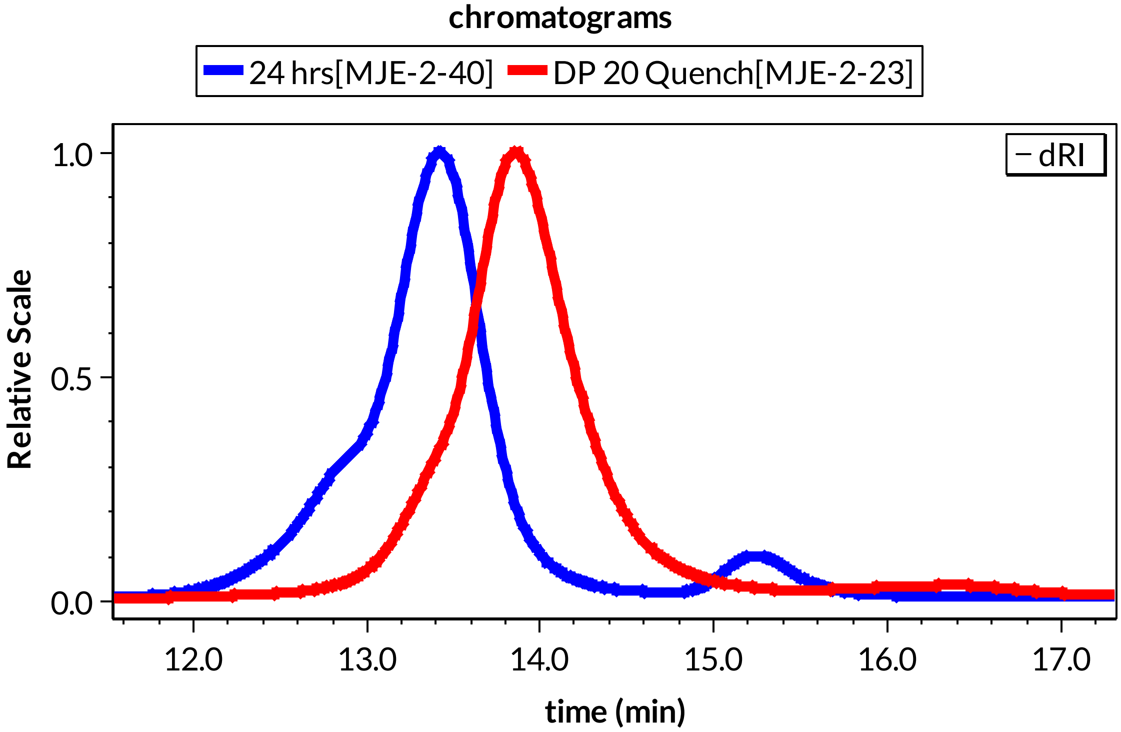

Figure S38: dRI traces of REMP with target DP = 20 for **PS-MM** with M_n = 4.6 kDa (red) and M_n = 8.3 kDa (blue). The blue signal at ca. 15.25 min is residual **PS-MM** with M_n = 8.3 kDa.

Table S3: GPC-MALS data comparing REMP performed on **PS-MM** of varying M_n

**Macromonomer M_n (kDa)[b]:**	**Theoretical DP[a]:**	**Experimental DP[b]:**	**Theoretical M_n (kDa) [a]:**	**Experimental M_n (kDa) [b]:**	**Experimental M_w (kDa)[b]:**	**$Đ$[b]:**
4.6	20	28	90	130	140	1.2
8.3	20	29	170	240	290	1.2

[a]Calculated by [MM]/[I], where [MM] is the molar amount of macromonomer and [I] is the molar amount of **pyr-CB6**.
[b]Determined by GPC-MALS.

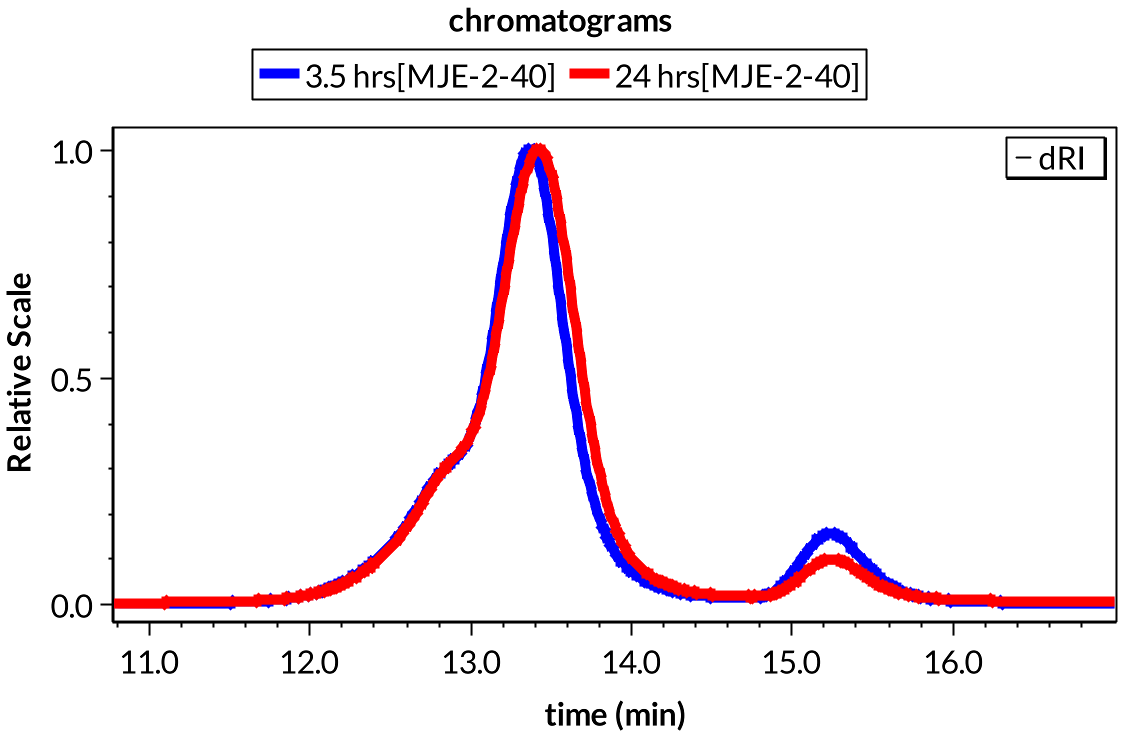

Figure S39: dRI traces of REMP with target DP = 20 for **PS-MM** with M_n = 8.3 kDa at 3.5 hours (blue) and 24 hours (red). The signal at ca. 15.25 minutes is residual **PS-MM** with M_n = 8.3 kDa. The final polymer has M_n = 240 kDa, M_w = 290 kDa, $Đ$ = 1.2, and *dn*/*dc* = 0.1119 (measured directly via batch injection).

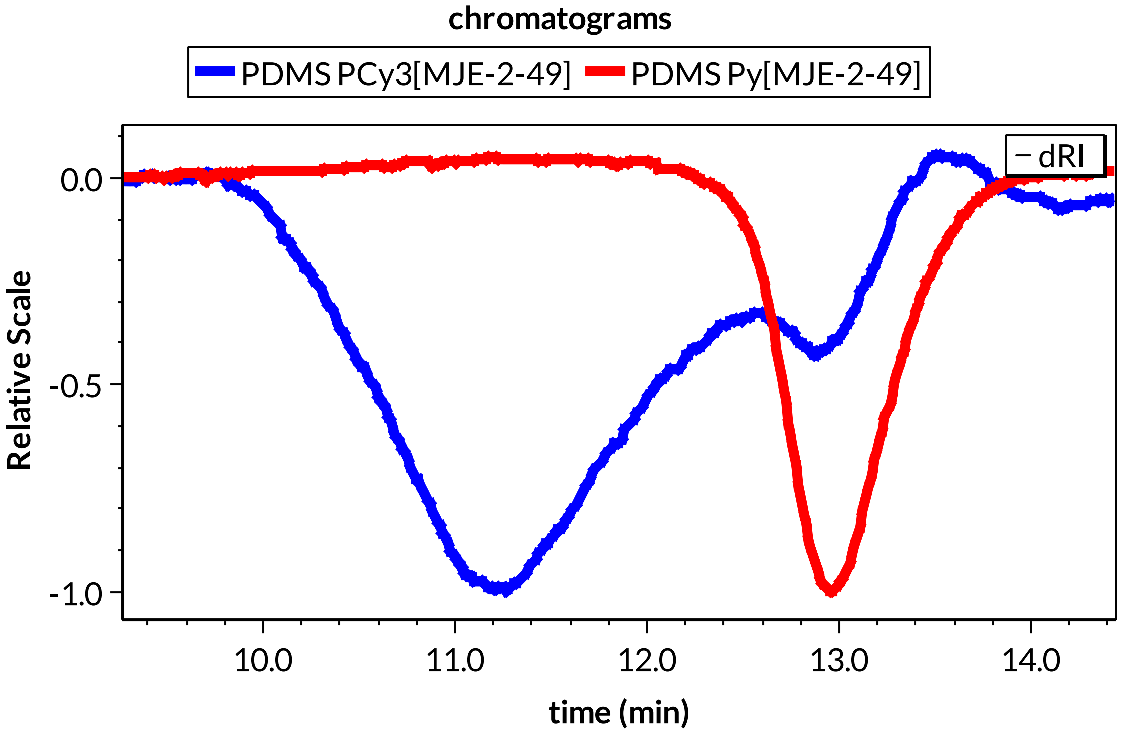

Figure S40: dRI traces of REMP with target DP = 20 for **PDMS-MM** with M_n = 7.0 kDa initiating with **pyr-CB6** (red) or **PCy$_3$-CB6** (blue). See **Table 1** in the main text for GPC characterization data. Attempts to REMP **PS-MM** (M_n = 4.4 kDa) in the absence of pyridine (i.e., using **PCy$_3$-CB6** initiator) produced polymers too viscous to filter for GPC analysis (Target DP = 20).

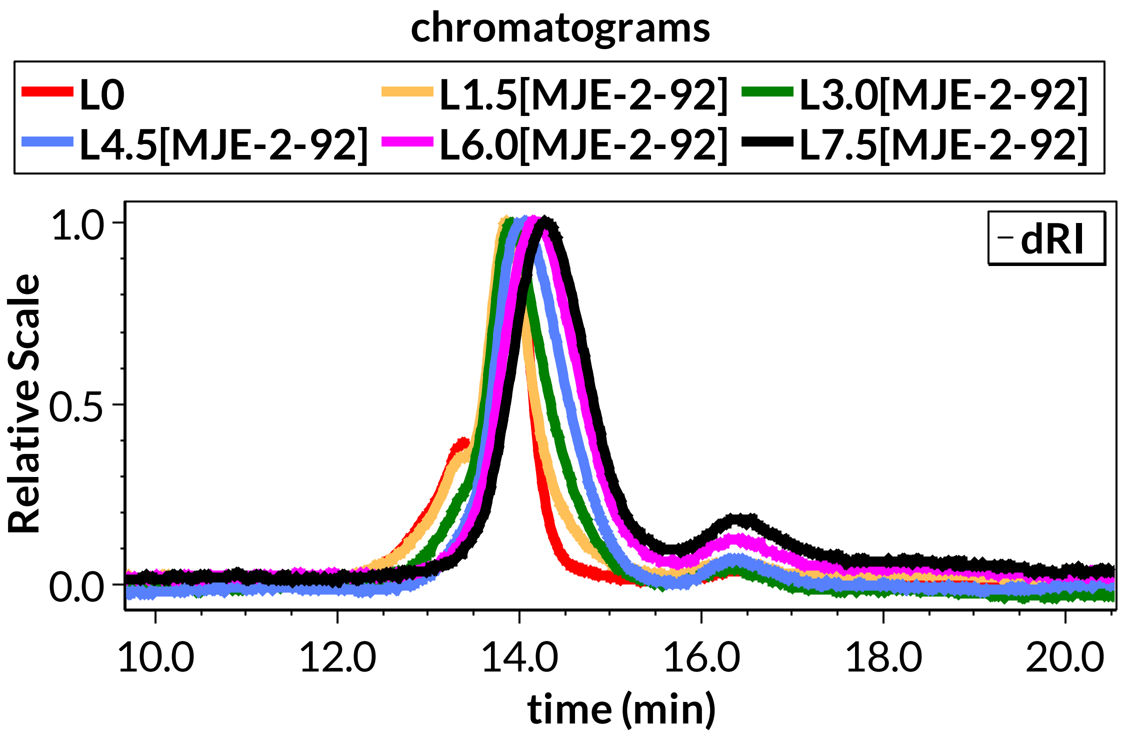

Figure S41: dRI traces of linear **PS-BBP** with increasing ball-mill grinding time. At t_0, the polymer had M_n = 200 kDa, M_w = 260 kDa, $Đ$ = 1.3, and *dn*/*dc* = 0.1706 (measured directly via batch injection).

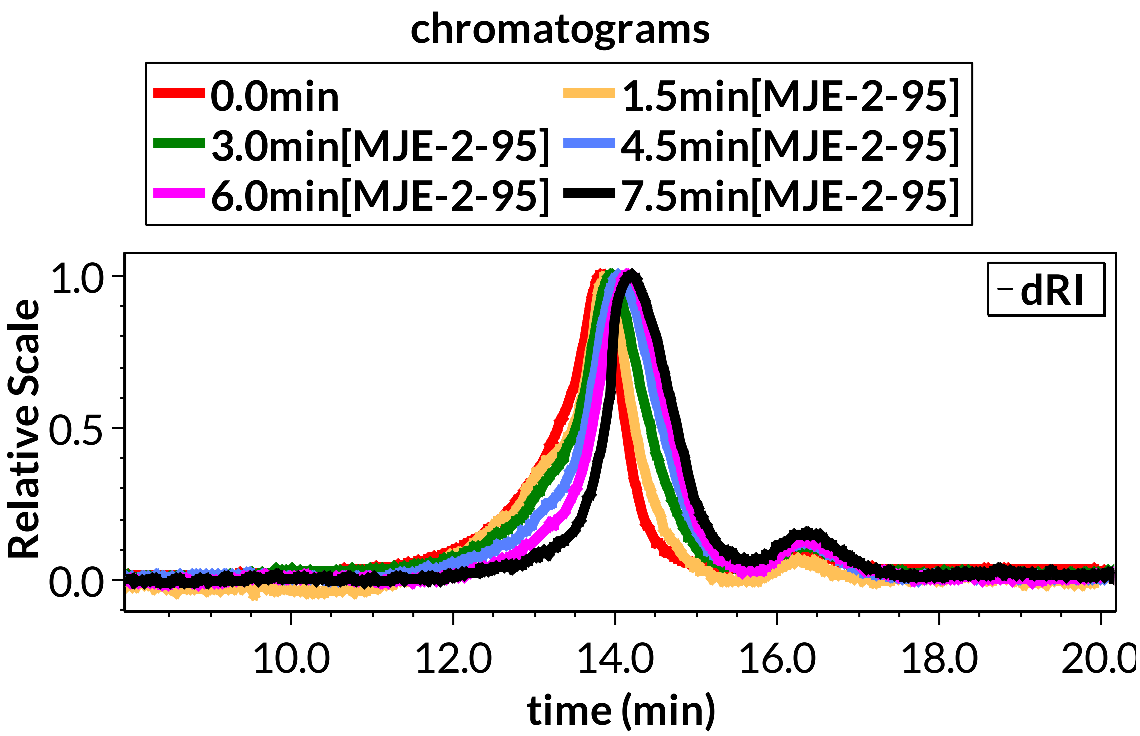

Figure S42: dRI traces of cyclic **PS-BBP** with increasing ball-mill grinding time. At t_0, the polymer had M_n = 250 kDa, M_w = 330 kDa, $Đ$ = 1.3, and *dn*/*dc* = 0.1119 (measured directly via batch injection)

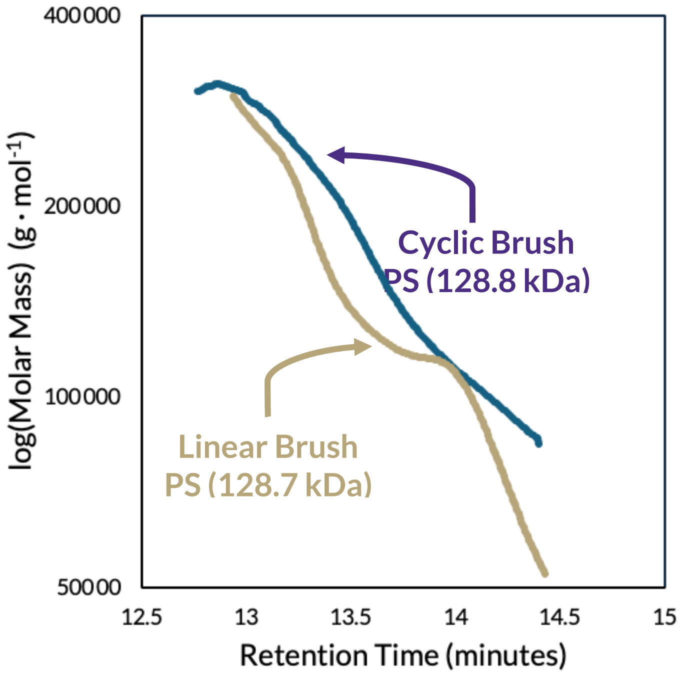

Figure S43: Molar mass vs. retention time plots for both cyclic and linear **PS-BBPs**. Both samples were prepared from **PS-MM** with M_n = 4.6 kDa.

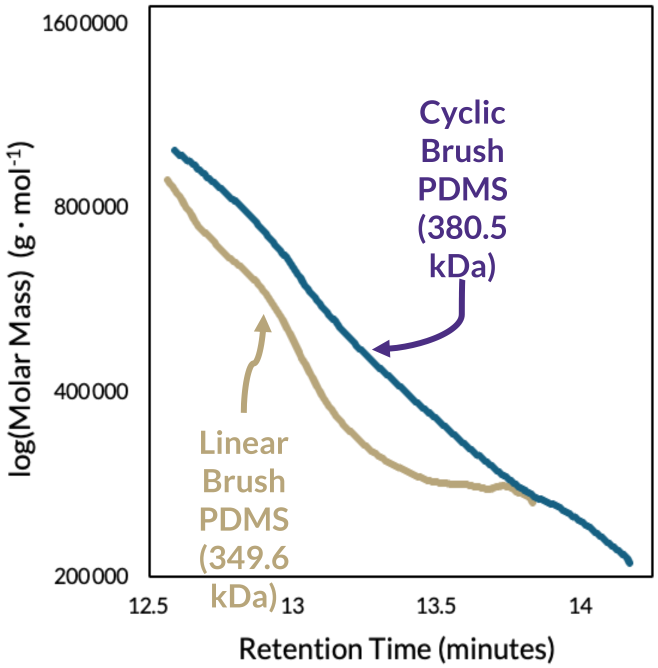

Figure S44: Molar mass vs. retention time plots for both cyclic and linear **PDMS-BBPs**. Both samples were prepared from **PDMS-MM** with M_n = 6.3 kDa.

5: GPC-MALS-IV DATA:

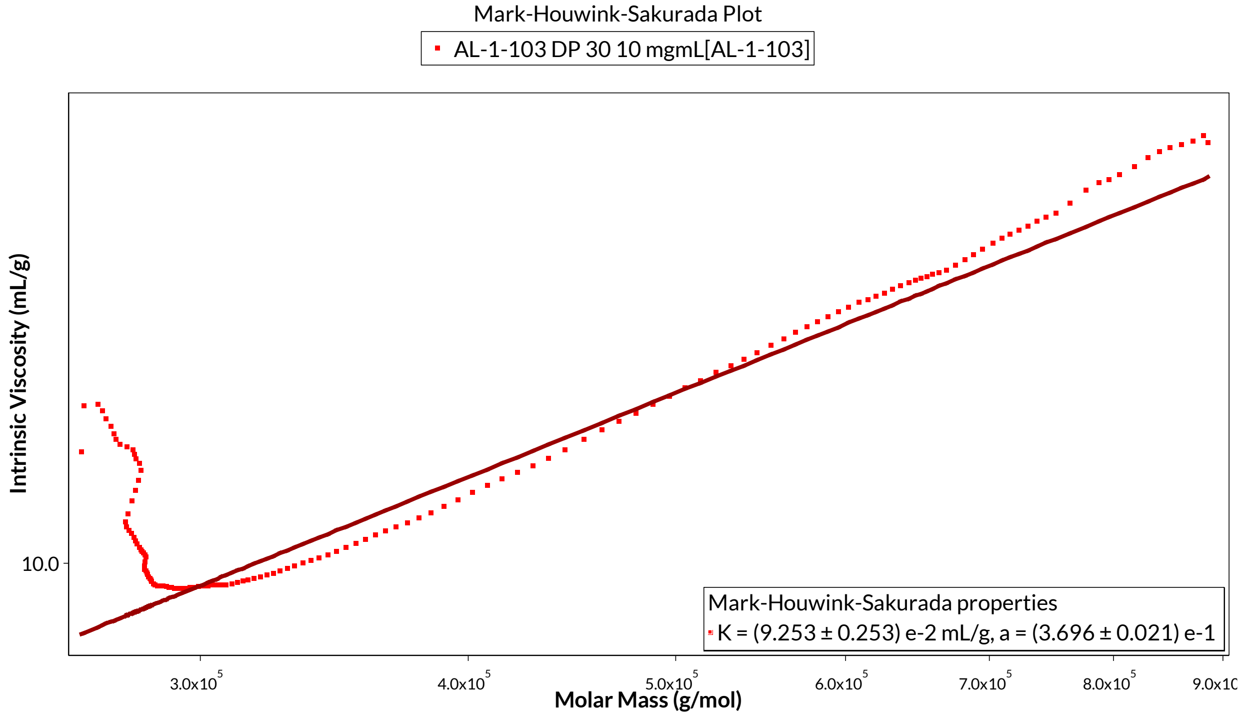

Figure S45: Representative MHS plot of linear **PDMS-BBP** with M_n = 350 kDa, M_w = 380 kDa, *Đ* = 1.1, and *dn*/*dc* = –0.0177 (measured directly via batch injection). Target DP = 30 from **PDMS-MM** with M_n = 6.3 kDa.

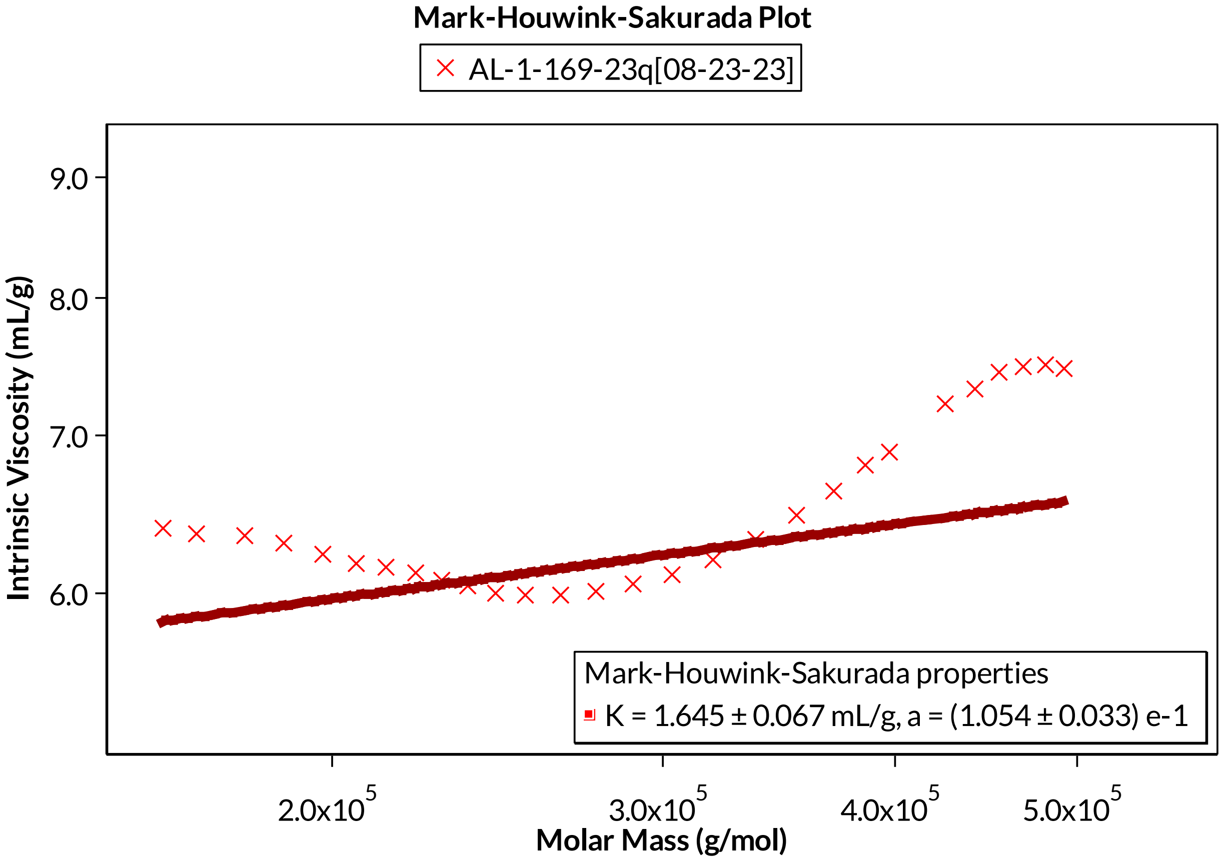

Figure S46: Representative MHS plot of cyclic **PDMS-BBP** with M_n = 260 kDa, M_w = 280 kDa, $Đ$ = 1.1, and dn/dc = –0.0115 (measured directly via batch injection). Target DP = 10 from **PDMS-MM** with M_n = 6.3 kDa.

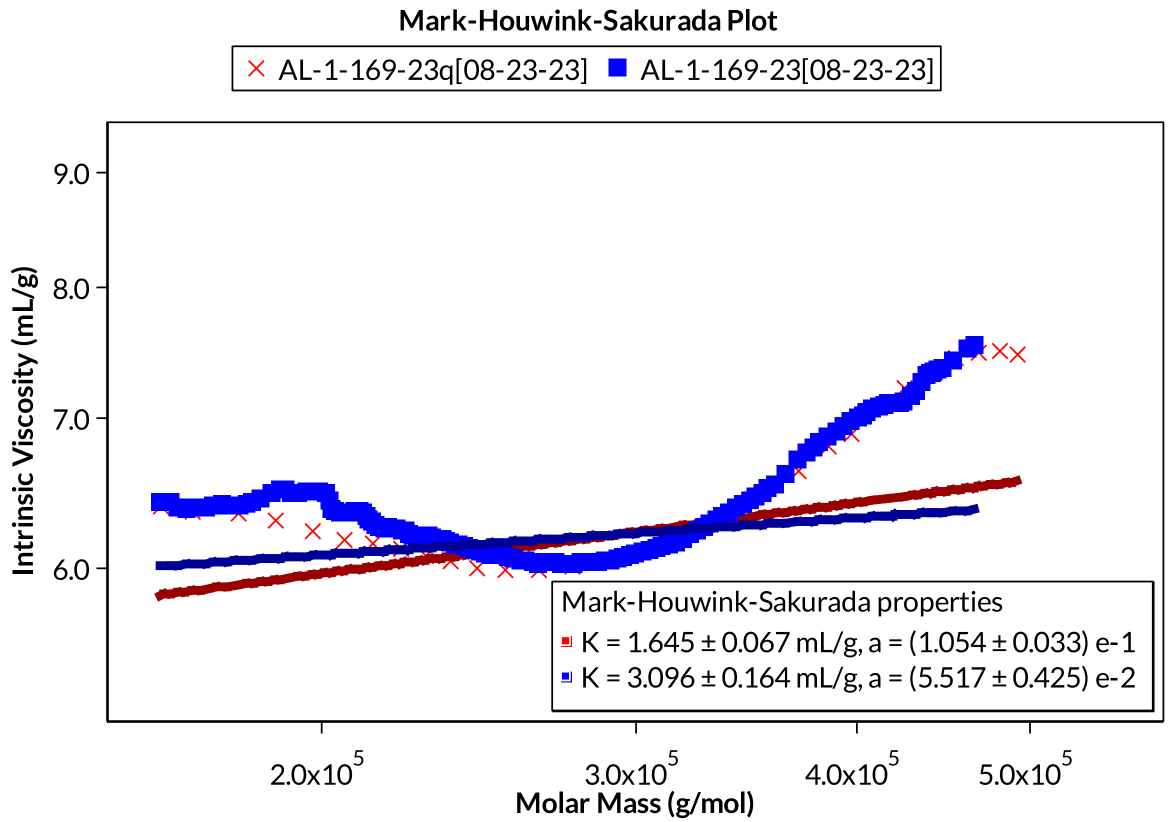

Figure S47: Representative MHS plot of quenched/unquenched cyclic **PDMS-BBP** samples. Target DP = 10 from **PDMS-MM** with M_n = 6.3 kDa. See **Table S2** for GPC data.

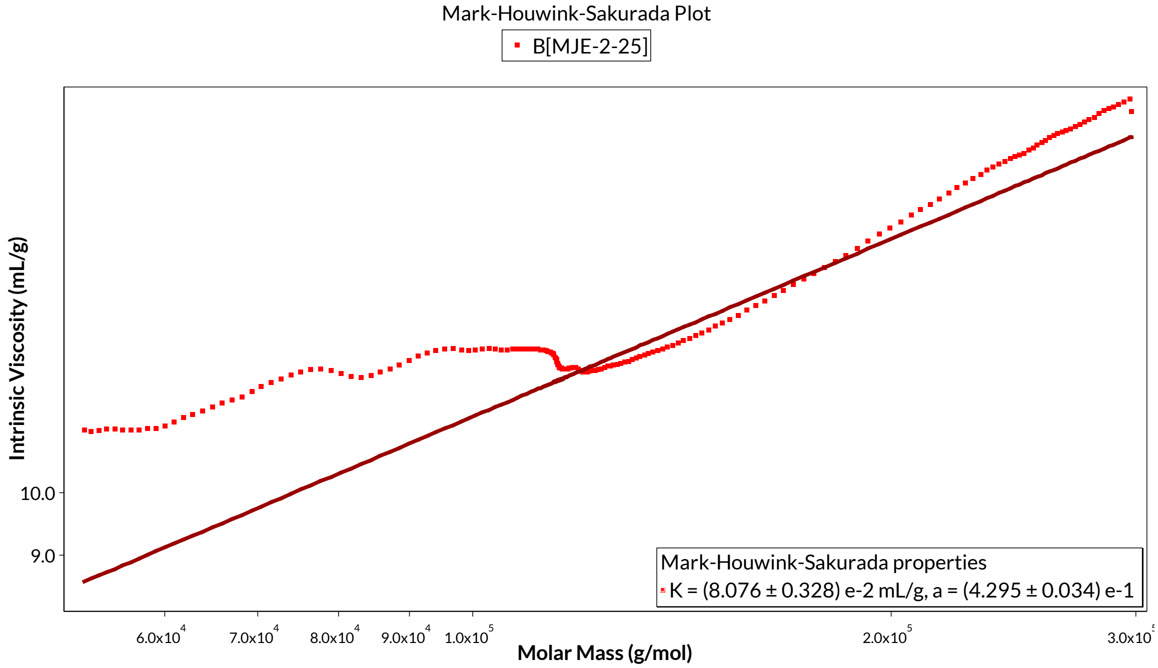

Figure S48: Representative MHS plot of linear **PS-BBP** with M_n = 130 kDa, M_w = 140 kDa, $Đ$ = 1.1, and dn/dc = 0.1706 (measured directly via batch injection). Target DP = 50 from **PS-MM** with M_n = 4.6 kDa.

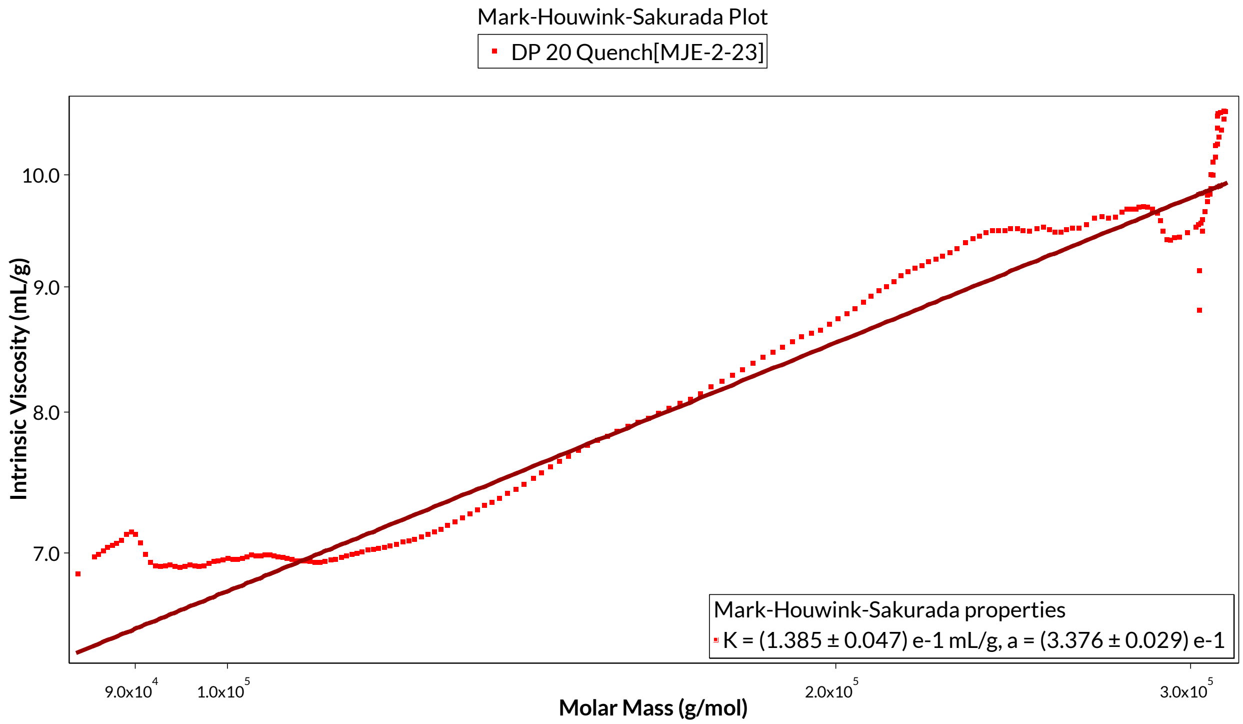

Figure S49: Representative MHS plot of cyclic **PS-BBP** with M_n = 130 kDa, M_w = 140 kDa, $Đ$ = 1.1, and dn/dc = 0.1119 (measured directly via batch injection). Target DP = 20 from **PS-MM** with M_n = 4.6 kDa.

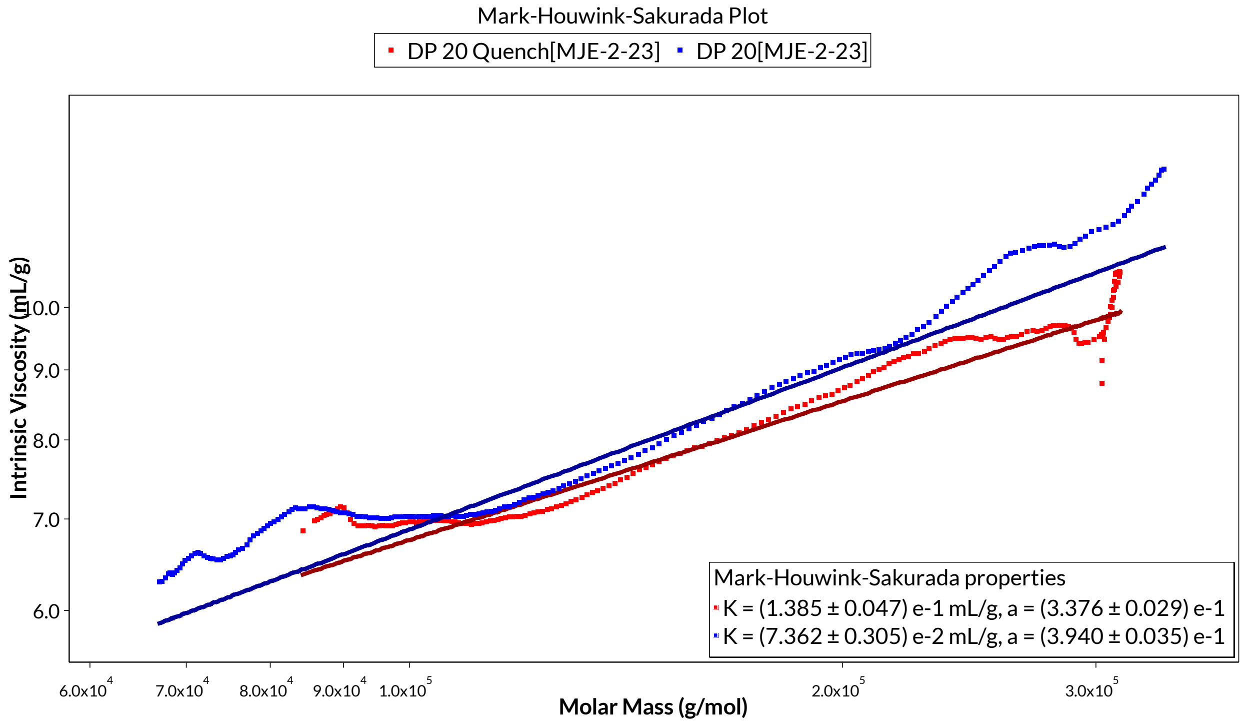

Figure S50: Representative MHS plot of quenched and unquenched cyclic **PS-BBP** samples. Target DP = 20 from **PS-MM** with M_n = 4.6 kDa. See **Table S1** for GPC data.

6: BATCH INJECTION PROCEDURES AND DATA:

Determination of Brush Polymer *dn/dc* Values via Batch Injection:
Bottlebrush polymers were prepared as described above. The **PS-MM** used had M_n = 4.6 kDa and the **PDMS-MM** used had M_n = 7.0 kDa. All polymerizations were run with target DP = 30. To obtain accurate concentrations, the samples were washed three times with pentane after the normal workup procedure, and the pentane was removed *in vacuo*. The samples were subsequently dried for a minimum of 36 h under high vacuum (<1 mmHg). Ethanol stabilized chloroform was used to prepare the solutions. The solvent was stirred overnight in a solvent jar with an aluminum foil covering, and holes were poked in the foil to ensure that the solvent was at equilibrium with dissolved air. Stock solutions were prepared gravimetrically, using the density of the solvent obtained from the lot information on the solvent bottle for maximum accuracy. Dilutions were performed by withdrawing solvent from the stock using glass microsyringes with Teflon plungers and diluting into volumetric flasks. The Optilab NEON dRI detector was purged prior to injecting each polymer by injecting 5 mL of the same solvent used to prepare samples into the detector via syringe pump at a flow rate of 0.2 mL/min. Polymer samples were injected sequentially in order of increasing concentration directly into the dRI detector using a syringe pump at a flow rate of 0.2 mL/min; typically, around 2 mL were required to obtain a stable reading. After all concentrations (between 4 and 6 samples) for each polymer sample were injected, the dRI detector was purged again by injecting an additional 5 mL of the same chloroform used to prepare the samples and re-zeroed. The data were processed and the *dn/dc* values were determined using Wyatt ASTRA software. The concentrations used for each polymer, as well as the corresponding dRI readings and calculated *dn/dc* values, are summarized in **Table S4**. The relevant dRI traces and calibration curves are reproduced in **Figures S50-S57**.

Table S4: Batch Injection dRI Data for Bottlebrush Polymers

Concentration (mg/mL)	Differential Refractive Index
Linear **PS-BBP**	
0.095	$1.512 * 10^{-5}$
0.190	$3.042 * 10^{-5}$
0.285	$5.256 * 10^{-5}$
0.381	$6.884 * 10^{-5}$
0.571	$1.113 * 10^{-4}$
0.952	$1.580 * 10^{-4}$
dn/dc = 0.1706 ± 0.0106 with R^2 = 0.9849	

Cyclic **PS-BBP**	
0.095	$5.994 * 10^{-6}$
0.189	$2.128 * 10^{-5}$
0.284	$3.541 * 10^{-5}$
0.378	$4.472 * 10^{-5}$
0.567	$7.028 * 10^{-5}$
0.945	$1.021 * 10^{-4}$
dn/dc = 0.1119 ± 0.0071 with R^2 = 0.9840	

Linear **PDMS-BBP**	
0.103	$-1.824 * 10^{-5}$
0.206	$-1.831 * 10^{-5}$
0.826	$-3.181 * 10^{-5}$
1.032	$-3.306 * 10^{-5}$
dn/dc = –0.0177 ± 0.0019 with R^2 = 0.9771	

Cyclic **PDMS-BBP**	
0.132	$-2.144 * 10^{-5}$
0.264	$-2.155 * 10^{-5}$
0.528	$-2.643 * 10^{-5}$
0.793	$-2.861 * 10^{-5}$
1.057	$-3.347 * 10^{-5}$
1.321	$-3.52 * 10^{-5}$
dn/dc = –0.0115± 0.0012 with R^2 = 0.9562	

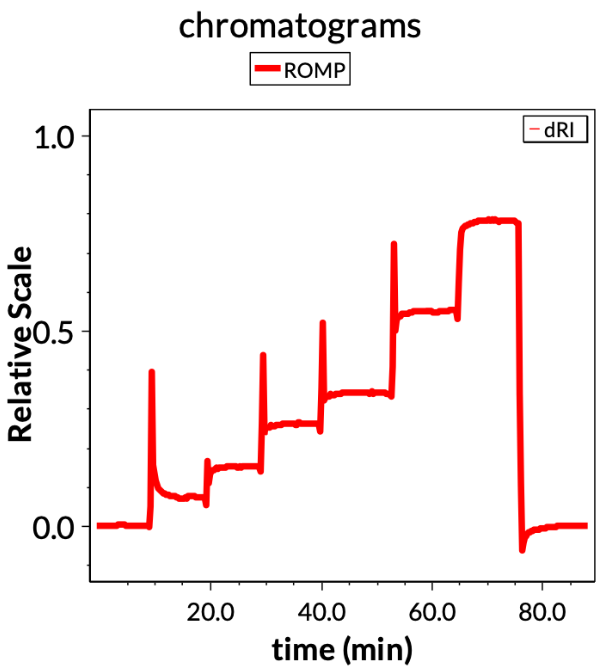

Figure S51: dRI traces for linear **PS-BBP** batch injections. Target DP = 30 from **PS-MM** with M_n = 4.6 kDa.

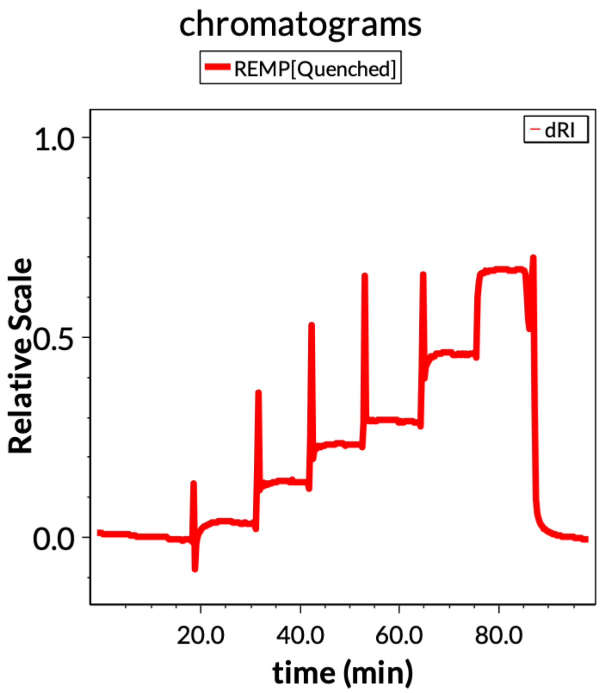

Figure S52: dRI traces for cyclic **PS-BBP** batch injections. Target DP = 30 from **PS-MM** with M_n = 4.6 kDa.

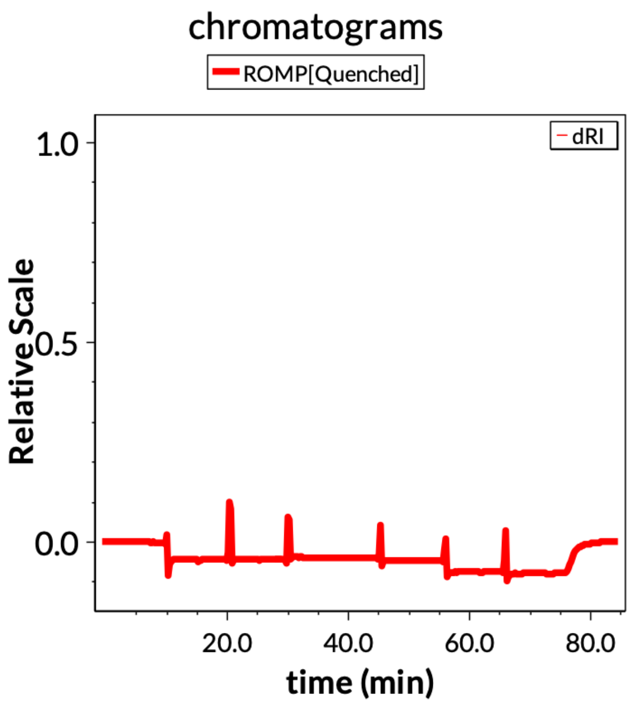

Figure S53: dRI traces for linear **PDMS-BBP** batch injections. Target DP = 30 from **PDMS-MM** with M_n = 7.0 kDa.

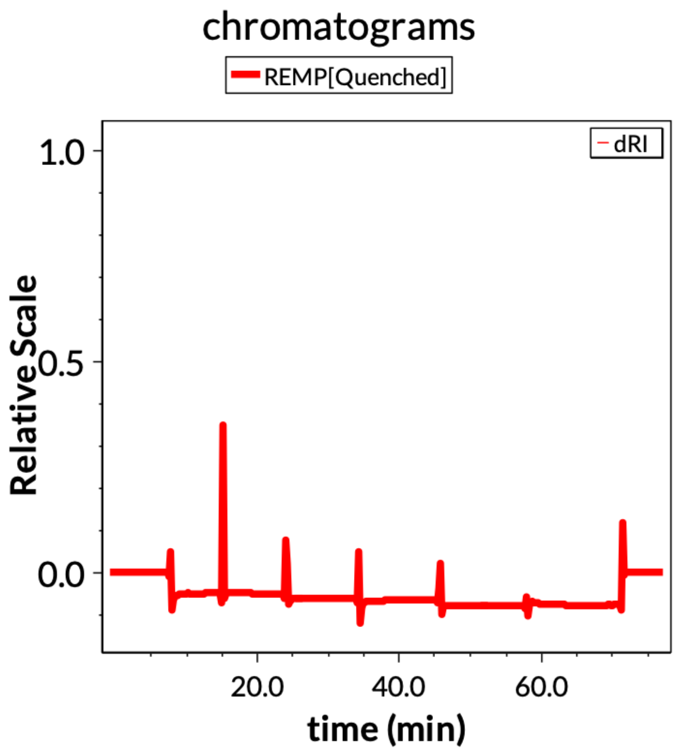

Figure S54: dRI traces for cyclic **PDMS-BBP** batch injections. Target DP = 30 from **PDMS-MM** with M_n = 7.0 kDa.

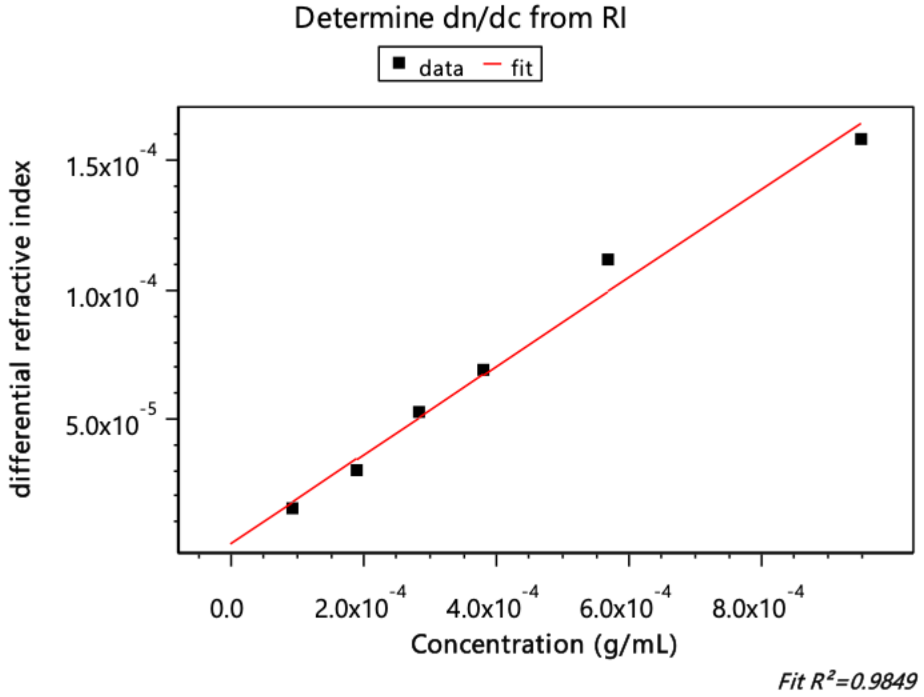

Figure S55: *dn*/*dc* calibration curve for linear **PS-BBP** via batch injection. Target DP = 30 from **PS-MM** with M_n = 4.6 kDa.

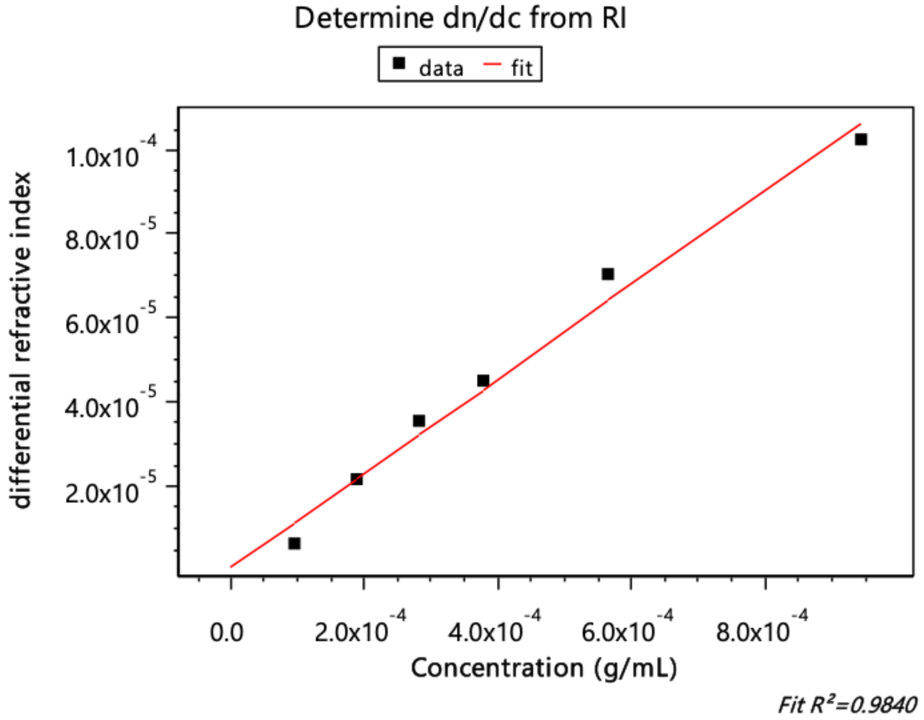

Figure S56: *dn*/*dc* calibration curve for cyclic **PS-BBP** via batch injection. Target DP = 30 from **PS-MM** with M_n = 4.6 kDa.

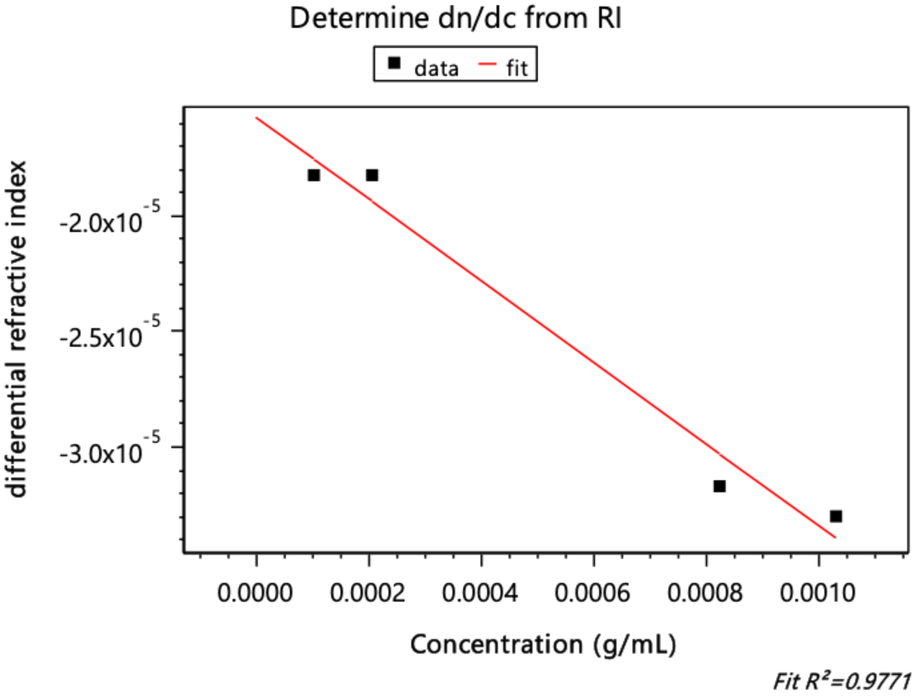

Figure S57: *dn*/*dc* calibration curve for linear **PDMS-BBP** via batch injection. Target DP = 30 from **PDMS-MM** with M_n = 7.0 kDa.

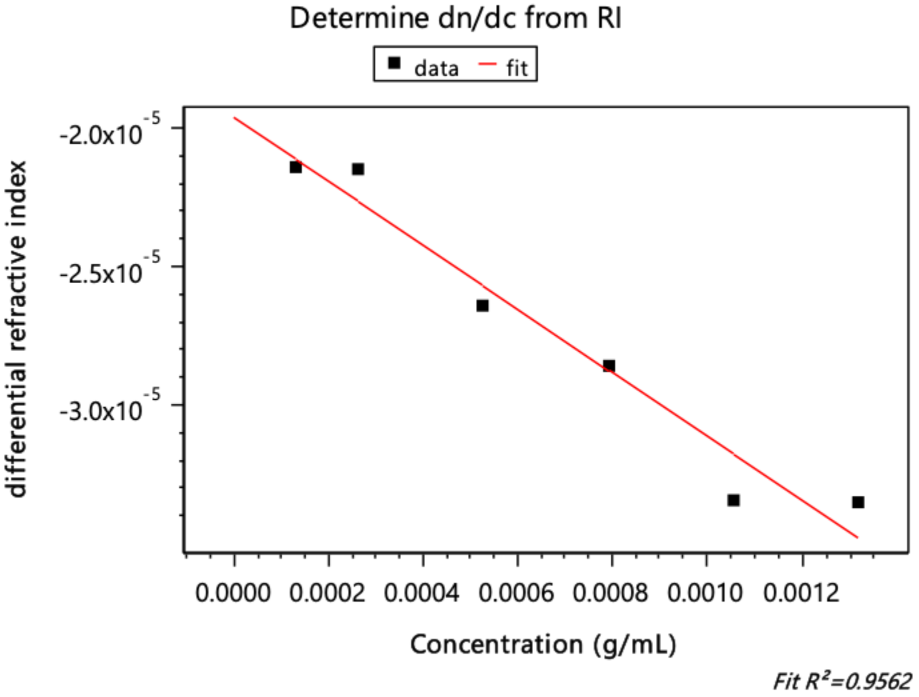

Figure S58: *dn*/*dc* calibration curve for cyclic **PDMS-BBP** via batch injection. Target DP = 30 from **PDMS-MM** with M_n = 7.0 kDa.

7: THERMAL CHARACTERIZATION:

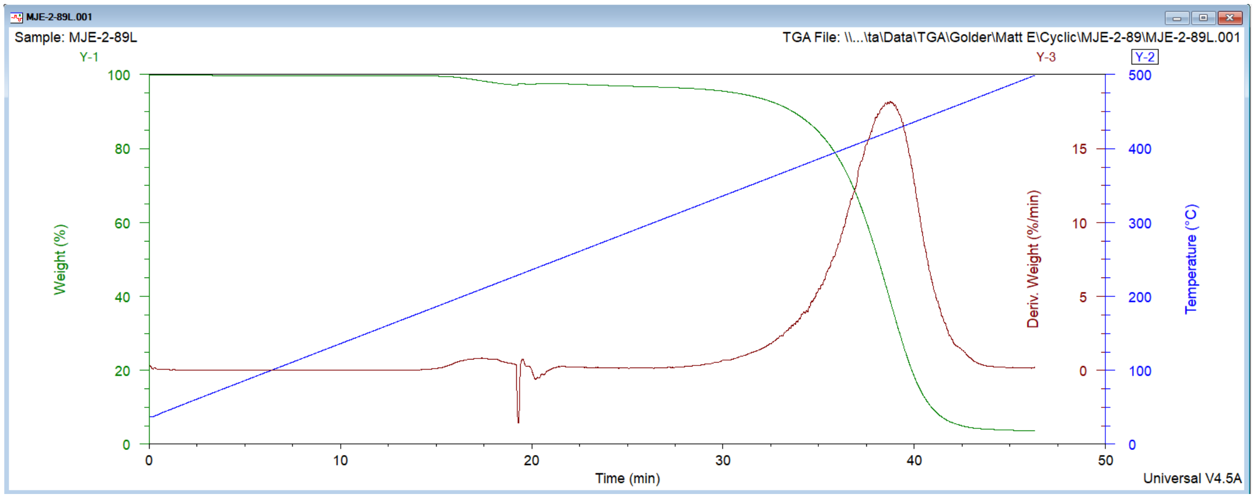

Figure S59: Thermogravimetric Analysis of linear **PS-BBP** (5.256 mg) with M_n = 200 kDa, M_w = 260 kDa, $Đ$ = 1.3, and dn/dc = 0.1706 (measured directly via batch injection). $\boldsymbol{T_{d(10\%)}}$ **= 374 °C**

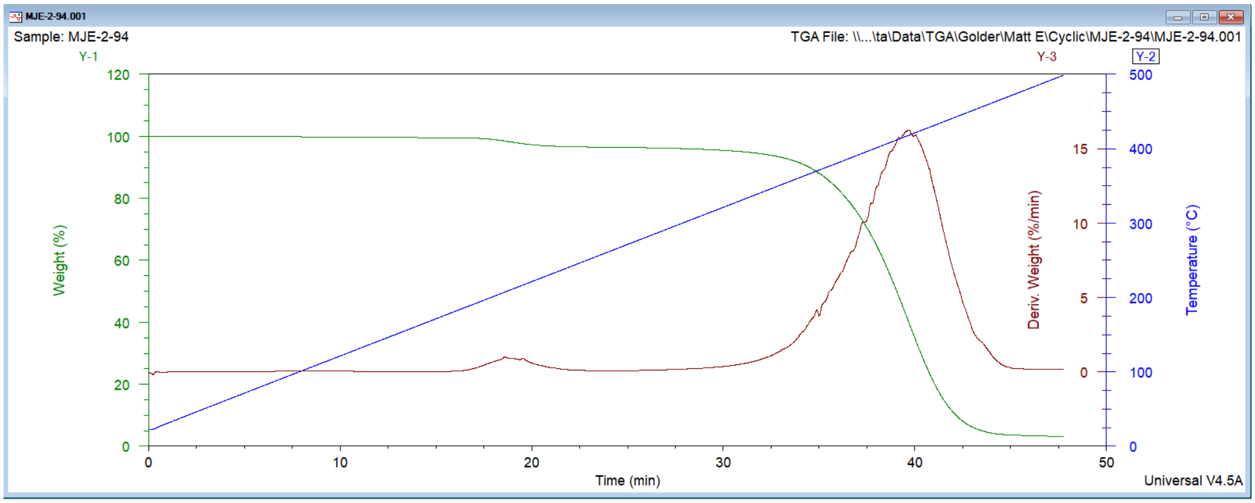

Figure S60: Thermogravimetric Analysis of cyclic **PS-BBP** (10.614 mg) with M_n = 250 kDa, M_w = 330 kDa, $Đ$ = 1.3, and dn/dc = 0.1119 (measured directly via batch injection). $\boldsymbol{T_{d(10\%)}}$ **= 368 °C**

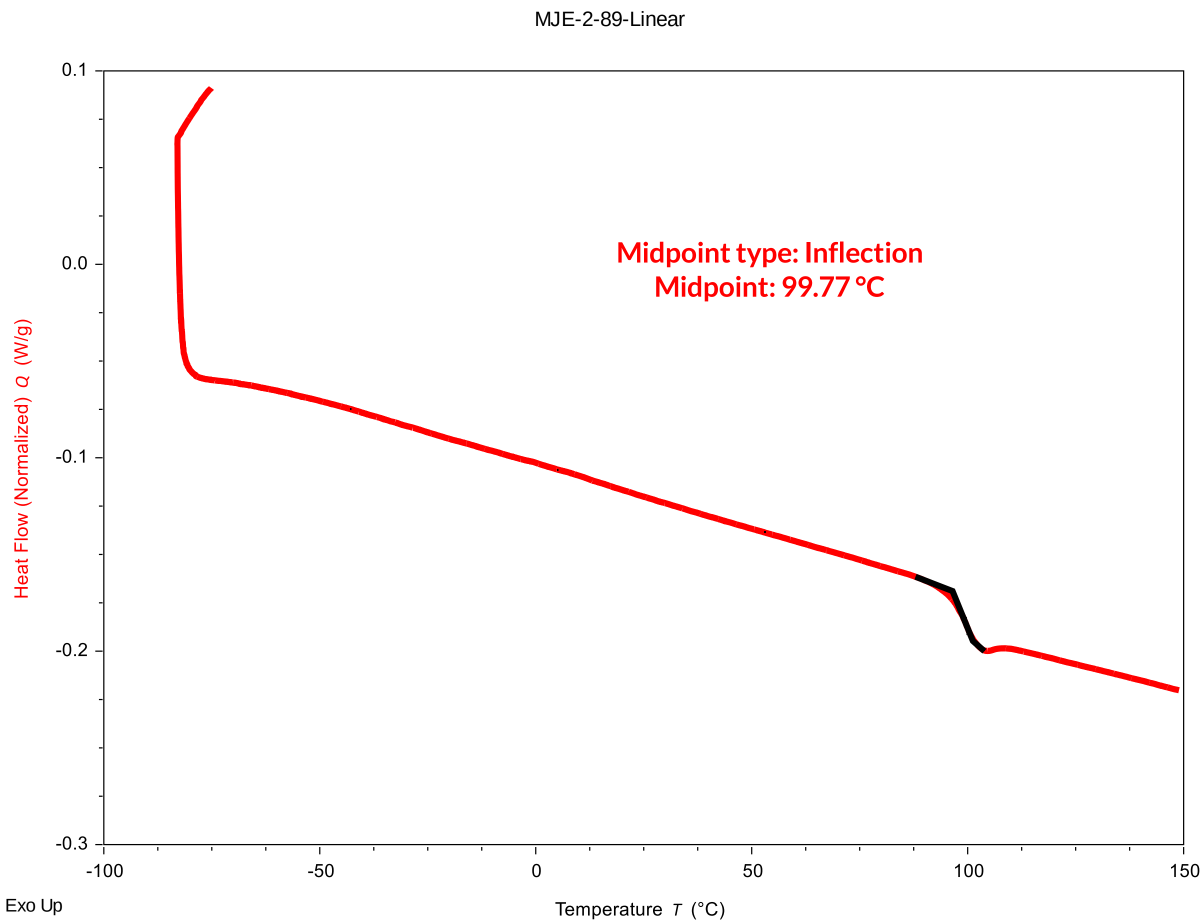

Figure S61: Differential Scanning Calorimetry of linear **PS-BBP** (7.3 mg) with M_n = 200 kDa, M_w = 260 kDa, $Đ$ = 1.3, and dn/dc = 0.1706 (measured directly via batch injection). Thermal features were measured on the second heating of a heat-cool-heat experiment, with a heating rate of 5 °C/min and a cooling rate of 15 °C/min. $\boldsymbol{T_g}$ **= 100 °C**

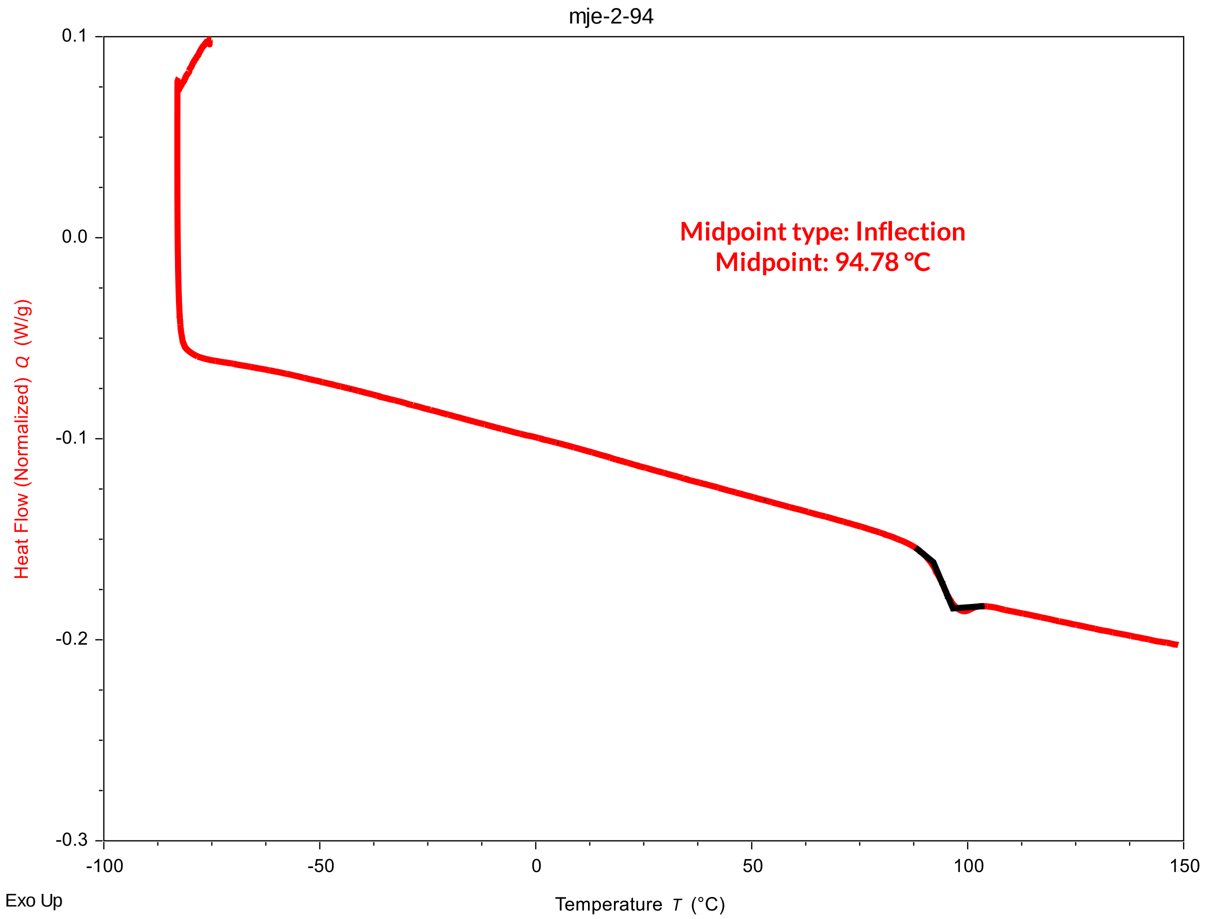

Figure S62: Differential Scanning Calorimetry of cyclic **PS-BBP** (12.7 mg) with M_n = 250 kDa, M_w = 330 kDa, *Đ* = 1.3, and *dn*/*dc* = 0.1119 (measured directly via batch injection). Thermal features were measured on the second heating of a heat-cool-heat experiment, with a heating rate of 5 °C/min and a cooling rate of 15 °C/min. $\boldsymbol{T_g}$ **= 95 °C**

8: BALL-MILL GRINDING KINETICS EXPERIMENTAL PROCEDURES AND DATA:

Experimental Protocol:

The rate constants for the mechanochemical degradation of cyclic and linear **PS-BBPs** were determined following a literature procedure[8,9]. Cyclic and linear polymers (15.2 mg each) were weighed into separate 5 mL stainless steel screw cap jars, and the jars were each charged with two 8 mm stainless steel balls. The polymers were milled at 20 Hz. After 1.5 minutes, one ball was removed from each jar and transferred to a 7 mL glass vial. The balls were replaced with clean, identical balls, and the polymers were milled at 20 Hz for a further 1.5 minutes before this process was repeated. The polymers were milled for a total of 7.5 minutes and a total of five 1.5-minute timepoints were withdrawn as described. At the end of the experiment, each ball was rinsed with 2 mL of chloroform, and the solutions were filtered through 0.2 μm syringe filters for GPC-MALS analysis.

GPC-MALS Analysis Protocol and M_n *determination:*

Since cyclic polymers degrade to form linear byproducts[10], the *dn/dc* values of polymer mixtures from degraded cyclic polymers will be intermediate between the cyclic polymer *dn/dc* and the linear polymer *dn/dc*. Since accounting for "true" *dn/dc* values of these mixtures would be prohibitively unwieldy, we estimated the molar masses of cyclic polymer degradation products by using the *dn/dc* of linear brush PS (0.1706) for all samples, excepting cyclic t_0, in which case the cyclic brush polymer *dn/dc* of 0.1119 was used (since no degradation to linear products had yet occurred). Notably, this estimation represents the most conservative approach in that it likely overestimates the *dn/dc* of cyclic polymer degradation products at early timepoints (as the "true" *dn/dc* will be a weighted average of the larger magnitude linear *dn/dc* and the smaller magnitude cyclic *dn/dc* that depends on the actual extent of polymer degradation). This, in turn, results in the underestimation of M_n in these early timepoints and therefore represents an overestimation of the rate of cyclic brush PS degradation.

Furthermore, an accurate description of the degradation products necessitates accounting for cleavage of the polymer brush arms from the polymer brush backbone, a process that has been previously observed in BMG experiments on polymer brushes[9]. This was achieved by integrating the main polymer peak and macromonomer peaks (normalized to sum to 1.0) and subtracting out the relative amount of residual macromonomer present in the relevant t_0 timepoints. As described in the literature[9], a weighted average of the parent peak $M_{n(parent)}$ and macromonomer peak $M_{n(macromonomer)}$ was used to estimate $M_{n(corrected)}$, according to the following equation:

$$M_{n(corrected)} = M_{n(parent)} * (1 - normalized\ area_{macromonomer\ peak}) + M_{n(macromonomer)} * normalized\ area_{macromonomer\ peak}$$

Determination of rate constants:

The degradation rate constats k_{deg} were determined by fitting our data to the following model, which was developed by Sato and Nalepo[8] and has been used to determine rate constants for the BMG degradation of bottlebrush polymers previously[9]:

$$\frac{1}{M_{n,t}} = \frac{k_{deg}}{M_{n,repeat\ unit}} t + \frac{1}{M_{n,0}}$$

Thus, plotting $1/M_{n(corrected)}$ against ball-milling time, performing a linear regression, and multiplying the slope of the line of best fit by the molar mass of the macromonomer delivers the value of the rate constant k_{deg}. The raw data obtained from these experiments are tabulated below; see the main text for plots with regression analysis.

Table S5: Linear PS-BBP BMG Degradation Data

Linear Brush PS		
**Milling Time (minutes):**	**$M_{n(corr)}$ (g/mole):**	**1/$M_{n(corr)}$ (mole/g):**
0.0	195,600	$5.11 * 10^{-6}$
1.5	125,512	$7.97 * 10^{-6}$
3.0	122,400	$8.17 * 10^{-6}$
4.5	96,560	$1.04 * 10^{-5}$
6.0	65,924	$1.52 * 10^{-5}$
7.5	51,140	$1.96 * 10^{-5}$
$y = (1.829 * 10^{-6})x + 4.198 *10^{-6}$ with $R^2 = 0.9218$		

Table S6: Cyclic PS-BBP BMG Degradation Data

Cyclic Brush PS		
**Milling Time (minutes):**	**$M_{n(corr)}$ (g/mole):**	**1/$M_{n(corr)}$ (mole/g):**
0.0	249,900	$4.00 * 10^{-6}$
1.5	169,600	$5.90 * 10^{-6}$
3.0	116,627	$8.57 * 10^{-6}$
4.5	95,850	$1.05 * 10^{-5}$
6.0	83,671	$1.20 * 10^{-5}$
7.5	66,879	$1.50 * 10^{-5}$
$y = (1.424 * 10^{-6})x + 3.960 *10^{-6}$ with $R^2 = 0.9930$		

Multiplying the slope for each trendline by $M_{n(macromonomer)}$ = 4,679 g/mole gives the following values for the rate constants of degradation:

$$k_{deg\ (linear)} = 8.56 * 10^{-3}\ mins^{-1}$$
$$k_{deg\ (cyclic)} = 6.66 * 10^{-3}\ mins^{-1}$$
$$\frac{k_{(deg,cyclic)}}{k_{(deg,linear)}} = 0.78$$

9: PREPARATION OF PDMS NETWORKS AND COMPRESSION TESTING

Preparation of resins:
Cyclic PDMS Resin: 283.2 mg (81 nanomoles) of cyclic **PDMS-BBP** were weighed into a 2 mL vial, and 236.3 (40 μmole) of **BisBP-PDMS** were weighed into a separate vial. The **PDMS-BBP** was dissolved in the minimum amount of DCM (~0.1 mL) and added to the **BisBP-PDMS** vial. The **PDMS-BBP** vial was rinsed 3 times with 0.25 mL DCM to make the transfer quantitative. The combined materials were thoroughly mixed by shaking the vial, then the volume was reduced with a gentle stream of nitrogen. The resin was dried further under high vacuum for 12 hours to remove the last of the solvent, and the resin was used within 24 hours of preparation.

Linear PDMS Resin: 276.5 mg (160 nanomoles) of linear **PDMS-BBP** were weighed into a 2 mL vial, and 230.8 (40 μmole) of **BisBP-PDMS** were weighed into a separate vial. The **PDMS-BBP** was dissolved in the minimum amount of DCM (~0.1 mL) and added to the **bisBP-PDMS** vial. The **PDMS-BBP** vial was rinsed 3 times with 0.25 mL DCM to make the transfer quantitative. The combined materials were thoroughly mixed by shaking the vial, then the volume was reduced with a gentle stream of nitrogen. The resin was dried further under high vacuum for 12 hours to remove the last of the solvent, and the resin was used within 24 hours of preparation.

Curing of resins:
UV Curing mold was constructed by binding two transparent fluorinated ethylene propylene film-coated glass slides separated by a (3.2 mm thick) spacer. Each resin was transferred into the mold using a pipette. The molds were irradiated with 365 nm UV light (Mightex, WheeLED, 200 mW/cm^2) for 2 hours (one hour on each side) to induce crosslinking. The networks were then removed from the mold and cut into compression cylinders using a 3 mm biopsy punch (Royaltek).

Compression testing:
Uniaxial compression testing was performed using a Universal Test Machine (Test Resources, 100-25-12) with a 43N load cell. Data was collected using a Newton Test Machine Controller. Exact dimensions of each specimen were measured with digital calipers prior to sample loading. All tests were conducted at room temperature (22 °C) using a crosshead rate of 1 mm/min until specimen failure or 90% strain. At least 3 specimens of each formulation were tested. The compressive modulus, strength, compressibility, and toughness were determined from the resultant stress-strain curve.

Table S7: Materials parameters from compression testing of PDMS-BBP polymer networks. Values are Reported as mean ± standard deviation (*±% standard deviation in parentheses*)

Sample Topology	Compressive Strength (kPa)	Strain at break (mm/mm)	Toughness (kJ/m^3)	Young's Modulus (kPa)	Swelling Ratio (Ethyl Acetate)[a]
Linear	1,896.54 ± 241.105<br>(*±13%*)	0.69 ± 0.01<br>(*±1.5%*)	289.408 ± 28.215<br>(*±10%*)	721.35 ± 42.64<br>(*±5.9%*)	148.30% ± 7.82%<br>(*±5.3%*)
Cyclic	3,337.50 ± 470.387<br>(*±14%*)	0.75 ± 0.02<br>(*±2.2%*)	454.594 ± 27.290<br>(*±6.1%*)	761.70 ± 116.8<br>(*±15%*)	191.22% ± 6.06%<br>(*±3.2%*)

[a]Swelling ratio is calculated via the following expression: $\text{Swelling Ratio} = \frac{mass_{equilibrium} - mass_{initial}}{mass_{initial}} * 100\%$

Results of Statistical Analyses:

Group Statistics

	VAR00002	N	Mean	Std. Deviation	Std. Error Mean
VAR00001	1.00	3	1896.5400	241.10547	139.20231
	2.00	3	3337.4033	470.38723	271.57819

Independent Samples Test

		Levene's Test for Equality of Variances		t-test for Equality of Means							
						Significance		Mean Difference	Std. Error Difference	95% Confidence Interval of the Difference	
		F	Sig.	t	df	One-Sided p	Two-Sided p			Lower	Upper
VAR00001	Equal variances assumed	1.334	.312	-4.721	4	.005	.009	-1440.86333	305.17536	-2288.16596	-593.56071
	Equal variances not assumed			-4.721	2.983	.009	.018	-1440.86333	305.17536	-2415.19708	-466.52959

Figure S63: Comparison of means via Independent Samples *t*-Test for linear and cyclic **PDMS-BBP** networks for the parameter *Compressive Strength*

T-Test

Group Statistics

	VAR00002	N	Mean	Std. Deviation	Std. Error Mean
VAR00005	1.00	3	.6853	.01088	.00628
	2.00	3	.7470	.01631	.00942

Independent Samples Test

		Levene's Test for Equality of Variances		t-test for Equality of Means							
						Significance		Mean Difference	Std. Error Difference	95% Confidence Interval of the Difference	
		F	Sig.	t	df	One-Sided p	Two-Sided p			Lower	Upper
VAR00005	Equal variances assumed	1.195	.336	-5.447	4	.003	.006	-.06165	.01132	-.09308	-.03023
	Equal variances not assumed			-5.447	3.485	.004	.008	-.06165	.01132	-.09500	-.02830

Figure S64: Comparison of means via Independent Samples *t*-Test for linear and cyclic **PDMS-BBP** networks for the parameter *Strain at Break*

Group Statistics

	VAR00002	N	Mean	Std. Deviation	Std. Error Mean
VAR00004	1.00	3	289.4077	28.21539	16.29016
	2.00	3	454.5937	27.28991	15.75584

Independent Samples Test

		Levene's Test for Equality of Variances		t-test for Equality of Means							
						Significance		Mean Difference	Std. Error Difference	95% Confidence Interval of the Difference	
		F	Sig.	t	df	One-Sided p	Two-Sided p			Lower	Upper
VAR00004	Equal variances assumed	.002	.969	-7.289	4	<.001	.002	-165.18600	22.66309	-228.10884	-102.26316
	Equal variances not assumed			-7.289	3.996	<.001	.002	-165.18600	22.66309	-228.13643	-102.23557

Figure S65: Comparison of means via Independent Samples *t*-Test for linear and cyclic **PDMS-BBP** networks for the parameter *Toughness*

T-Test

Group Statistics

	VAR00002	N	Mean	Std. Deviation	Std. Error Mean
VAR00006	1.00	3	721.3500	42.64363	24.62031
	2.00	3	761.6980	116.77165	67.41814

Independent Samples Test

		Levene's Test for Equality of Variances		t-test for Equality of Means							
						Significance		Mean Difference	Std. Error Difference	95% Confidence Interval of the Difference	
		F	Sig.	t	df	One-Sided p	Two-Sided p			Lower	Upper
VAR00006	Equal variances assumed	5.516	.079	-.562	4	.302	.604	-40.34803	71.77302	-239.62187	158.92581
	Equal variances not assumed			-.562	2.524	.310	.620	-40.34803	71.77302	-295.16563	214.46956

Figure S66: Comparison of means via Independent Samples *t*-Test for linear and cyclic **PDMS-BBP** networks for the parameter *Young's Modulus*

Group Statistics

	VAR00010	N	Mean	Std. Deviation	Std. Error Mean
VAR00011	1.00	4	191.2150	6.05692	3.02846
	2.00	4	148.2975	7.82207	3.91104

Independent Samples Test

		Levene's Test for Equality of Variances		t-test for Equality of Means							
						Significance		Mean Difference	Std. Error Difference	95% Confidence Interval of the Difference	
		F	Sig.	t	df	One-Sided p	Two-Sided p			Lower	Upper
VAR00011	Equal variances assumed	.280	.616	8.676	6	<.001	<.001	42.91750	4.94649	30.81387	55.02113
	Equal variances not assumed			8.676	5.646	<.001	<.001	42.91750	4.94649	30.62764	55.20736

Figure S67: Comparison of means via Independent Samples *t*-Test for linear and cyclic **PDMS-BBP** networks for the parameter *Swelling Ratio*

10: REFERENCES:

1 C. M. Bates, A. B. Chang, N. Momčilović, S. C. Jones and R. H. Grubbs, *Macromolecules*, 2015, **48**, 4967–4973.

2 A. L. Liberman-Martin, A. B. Chang, C. K. Chu, R. H. Siddique, B. Lee and R. H. Grubbs, *ACS Macro Lett.*, 2021, **10**, 1480–1486.

3 S. C. Radzinski, J. C. Foster, R. C. Jr. Chapleski, D. Troya and J. B. Matson, *J. Am. Chem. Soc.*, 2016, **138**, 6998–7004.

4 S. J. Scannelli, M. Alaboalirat, D. Troya and J. B. Matson, *Macromolecules*, 2023, **56**, 3838–3847.

5 S. Mukherjee, R. Xie, V. G. Reynolds, T. Uchiyama, A. E. Levi, E. Valois, H. Wang, M. L. Chabinyc and C. M. Bates, *Macromolecules*, 2020, **53**, 1090–1097.

6 M. S. Sanford, J. A. Love and R. H. Grubbs, *Organometallics*, 2001, **20**, 5314–5318.

7 S. P. Roche, M.-L. Teyssot and A. Gautier, *Tetrahedron Lett.*, 2010, **51**, 1265–1268.

8 T. Sato and D. E. Nalepa, *J. Appl. Polym. Sci.*, 1978, **22**, 865–867.

9 J. Noh, G. I. Peterson and T.-L. Choi, *Angew. Chem. Int. Ed.*, 2021, **60**, 18651–18659.

10 J. Noh, M. B. Koo, J. Jung, G. I. Peterson, K. T. Kim and T.-L. Choi, *J. Am. Chem. Soc.*, 2023, **145**, 18432–18438.
